# Comparative Effectiveness of AI-Assisted Telerehabilitation, Telerehabilitation, In-Person Care, and Usual Care for Chronic Nonspecific Low Back Pain: Bayesian Network Meta-Analysis

**DOI:** 10.2196/85410

**Published:** 2026-07-03

**Authors:** Peng Gu, Yuan Yan, Hao Tang, Yanqing Jia, Yonghao Wen, Zheng Zhang, Xiyan Zhao, Zhiwei Jia, Tianlin Wen

**Affiliations:** 1 Department of Orthopedics, Dongzhimen Hospital, Beijing University of Chinese Medicine Beijing China; 2 College of Clinical Medicine Qinghai University Xining, Qinghai China; 3 School of Medicine South China University of Technology Gangzhou China; 4 Department of Endocrinology, Guang’anmen Hospital, China Academy of Chinese Medical Sciences Beijing China; 5 Chongqing Traditional Chinese Medicine Hospital Chongqing China

**Keywords:** artificial intelligence, Bayesian network meta-analysis, chronic nonspecific low back pain, in-person rehabilitation, telerehabilitation, usual care

## Abstract

**Background:**

Guided exercise is central to rehabilitation for chronic nonspecific low back pain. Telerehabilitation enables remote delivery of guided exercise, but its effectiveness vs other rehabilitation modalities remains uncertain.

**Objective:**

This review systematically assessed the comparative efficacy of telerehabilitation, in-person rehabilitation (IPR), and usual care (UC) for improving pain, disability, kinesiophobia, and health-related quality of life in patients with chronic nonspecific low back pain. Telerehabilitation combined with artificial intelligence (TLRH-AI) was evaluated as an exploratory intervention because available evidence was limited.

**Methods:**

Following PRISMA (Preferred Reporting Items for Systematic Reviews and Meta-Analyses) 2020 guidelines, we searched randomized controlled trials in PubMed, Cochrane Library, Web of Science, and Embase from inception to April 30, 2026. A Bayesian network meta-analysis was conducted using R (version 4.4.1). Interventions were ranked using surface under the cumulative ranking curve (SUCRA) values. Evidence certainty was assessed using the GRADE (Grading of Recommendations Assessment, Development, and Evaluation) framework. Findings were interpreted considering heterogeneity, risk of bias, inconsistency, and estimated prediction intervals.

**Results:**

Among 2491 records, 20 randomized controlled trials involving 1854 participants were included. For pain intensity, IPR showed the greatest benefit at 4 weeks (low-certainty evidence), telerehabilitation at 8 weeks (moderate-certainty evidence), and telerehabilitation ranked highest at 12 weeks (SUCRA 87.2%; moderate-certainty evidence). For the Oswestry Disability Index–based disability, IPR ranked highest at 4 weeks (SUCRA 98.2%; low-certainty evidence) and 12 weeks (SUCRA 86.7%; low-certainty evidence), whereas telerehabilitation ranked highest at 8 weeks (SUCRA 90.4%; high-certainty evidence). For the Roland-Morris Disability Questionnaire–based disability, IPR was among the more effective interventions (SUCRA 67.3%; low-certainty evidence). For kinesiophobia, IPR ranked highest (SUCRA 99%; low-certainty evidence). For health-related quality of life, telerehabilitation significantly improved the physical component summary score (mean difference 6.05, 95% credible interval [CrI] 2.89-9.22; moderate-certainty evidence), whereas IPR showed a nonsignificant trend toward an improved mental component summary score (mean difference 2.79, 95% CrI −1.61 to 7.17; low-certainty evidence). Evidence for TLRH-AI remained limited and descriptive, suggesting possible short-term benefits with low to very low certainty. No significant small-study effects or global inconsistency were detected, although potentially important local inconsistency was observed in the 4-week Oswestry Disability Index comparison between UC and IPR.

**Conclusions:**

This review uniquely compared telerehabilitation, IPR, UC, and exploratory TLRH-AI within a Bayesian network meta-analysis. Unlike previous reviews focused mainly on telerehabilitation vs conventional care, it provides a comparative hierarchy across delivery models, follow-up windows, and outcomes while incorporating evidence certainty and heterogeneity. The findings support individualized rehabilitation selection. In practice, telerehabilitation may offer a scalable option for longer-term pain relief and physical function improvement, whereas IPR may remain important for supervised functional recovery and psychological support. TLRH-AI remains exploratory and should not guide clinical decision-making until adequately powered trials are available.

**Trial Registration:**

PROSPERO CRD420251146712; https://www.crd.york.ac.uk/PROSPERO/view/CRD420251146712

## Introduction

Chronic nonspecific low back pain (CNSLBP) refers to persistent low back pain lasting ≥12 weeks without an identifiable specific cause, such as tumor, infection, fracture, inflammatory spinal disease, or nerve root compression. It accounts for approximately 90% of all low back pain cases [1]. Its clinical presentation is complex and is often characterized by recurrent episodes of exacerbation and remission, which are associated with varying degrees of disability and reduced quality of life. Consequently, CNSLBP substantially affects patients’ social participation and work capacity [2]. Epidemiological studies indicate that low back pain is the leading cause of years lived with disability globally. According to the Global Burden of Disease Study, approximately 619 million people worldwide had low back pain in 2020, and this number is projected to increase to 843 million by 2050. The disease burden is particularly pronounced among aging populations and individuals engaged in labor-intensive occupations [3]. CNSLBP not only causes long-term health impairment but also leads to excessive health care resource use and substantial productivity loss, making it a major public health challenge worldwide.

Current international guidelines consistently emphasize nonpharmacological and noninvasive interventions for the management of CNSLBP. The World Health Organization’s 2023 guideline on the nonsurgical management of chronic primary low back pain recommends education, self-management, and exercise therapy as first-line measures [4]. Among these interventions, supervised exercise therapy is considered a core and evidence-based strategy [5,6]. This approach involves the development of individualized exercise prescriptions by rehabilitation physicians or physical therapists and is implemented under professional supervision. It typically includes core stability, aerobic, strength, and flexibility training. Supervised exercise therapy improves treatment adherence and ensures correct movement execution through continuous professional guidance. In addition, it may reduce kinesiophobia and psychosocial stress, thereby enhancing physical function and quality of life and helping to interrupt the pain–disuse–functional decline vicious cycle [7,8].

In clinical practice, conventional exercise-based rehabilitation guidance for CNSLBP is mainly delivered through usual care (UC) and in-person rehabilitation (IPR). UC typically comprises basic health education and nonsystematic exercise advice; its effectiveness largely depends on patients’ adherence, comprehension, and self-management capacity [9]. By contrast, IPR is characterized by structured face-to-face training with real-time supervision and movement correction. However, its implementation is constrained by therapist availability, facility requirements, scheduling demands, and transportation barriers. Long-term adherence also relies heavily on patient engagement, and access may be limited in remote or resource-constrained settings, posing ongoing challenges for broad dissemination and sustained delivery [10].

Telerehabilitation, an emerging model that integrates digital health and rehabilitation medicine, has gradually been adopted in the rehabilitation management of CNSLBP in recent years. Relying on internet-based platforms, mobile apps, or wearable devices, telerehabilitation can provide patients with personalized exercise prescriptions, rehabilitation guidance, real-time feedback, and follow-up monitoring. By overcoming limitations related to time and location, telerehabilitation may substantially improve the accessibility of rehabilitation services [11]. Based on this model, telerehabilitation combined with artificial intelligence (TLRH-AI) incorporates motion recognition and machine learning–based personalization into telerehabilitation systems. It can automatically evaluate movement performance in real time, provide immediate feedback, and dynamically tailor training intensity and progression to individual patients [12,13].

Compared with IPR, telerehabilitation has demonstrated unique advantages in situations involving limited health care resources, inconvenient transportation, irregular patient schedules, and public health emergencies such as the COVID-19 pandemic [14]. Existing evidence suggests that, in selected patient populations with musculoskeletal disorders, such as osteoarthritis and mixed chronic musculoskeletal pain, telerehabilitation can achieve outcomes comparable to those of IPR in terms of pain relief, functional improvement, and self-management capacity, as reported in systematic reviews and meta-analyses [15]. However, this comparability has mainly been reported in relatively stable clinical contexts. IPR still provides individualized guidance and remains essential for the assessment and management of patients with complex conditions. In particular, IPR is especially important for patients presenting with red flags requiring medical evaluation, those needing hands-on manual therapy or direct physical assessment, those with severe disability, impaired mental status, or loss of effective communication ability, and those with a history of major surgery, such as abdominal resection or spinal instrumentation, or physical limitations due to cardiovascular or cerebrovascular comorbidities.

Beyond clinical effectiveness, ethical and equity considerations are also critical for telerehabilitation and artificial intelligence (AI)–enhanced telerehabilitation. Disparities in socioeconomic status, geographic location, and racial or ethnic background can influence access to and uptake of telerehabilitation, while ethical concerns related to privacy, security, and autonomy are often insufficiently addressed. Evidence suggests that key domains, such as patient autonomy, privacy protection, and the management of adverse events, remain inadequately handled in current practice [16]. With accelerating population aging, older adults and other vulnerable groups face additional barriers related to digital access and digital literacy. Consequently, utilization and engagement may vary by age, sex, race or ethnicity, region, and socioeconomic position [17]. Therefore, ethical and equity considerations should be integrated into the design, implementation, and evaluation of telerehabilitation interventions.

However, the results of related clinical trials remain heterogeneous, and systematic comparisons across different rehabilitation models are lacking. At present, high-quality evidence clearly defining the relative roles of TLRH-AI, telerehabilitation, IPR, and UC in the management of CNSLBP remains insufficient. Therefore, this study aimed to conduct a Bayesian network meta-analysis (NMA) to integrate evidence from existing randomized controlled trials (RCTs) and compare the efficacy of TLRH-AI, telerehabilitation, IPR, and UC in improving pain, disability, kinesiophobia, and health-related quality of life. The findings are expected to provide more reliable evidence for clinical decision-making and public health policy development.

## Methods

### Protocol and Registration

This study was conducted in accordance with the PRISMA-NMA (Preferred Reporting Items for Systematic Reviews and Meta-Analyses extension for Network Meta-Analyses), PRISMA (Preferred Reporting Items for Systematic Reviews and Meta-Analyses), and PRISMA-S (Preferred Reporting Items for Systematic Reviews and Meta-Analyses – Search extension) 2020 guidelines [18-20]. A completed PRISMA 2020 27-item checklist is provided in Tables S1-S3 in Multimedia Appendix 1. The study protocol was registered with PROSPERO under registration number CRD420251146712.

### Search Strategy

Two independent reviewers applied the same predefined search strategy across 4 electronic databases, without restrictions on language or publication date: PubMed, the Cochrane Library, Web of Science, and Embase. The search strategy incorporated both Medical Subject Headings (MeSH) and free-text terms, with “telerehabilitation” and “nonspecific low back pain” serving as the key concepts. The search was reported in accordance with PRISMA-S to ensure that each component was fully described and reproducible [20]. Any discrepancies between the reviewers were resolved through discussion and consensus involving a third reviewer. In addition, manual searches of the reference lists of relevant meta-analyses and review articles were conducted to identify additional potentially eligible studies. The initial search was conducted on September 30, 2025, following iterative refinement of the search strategy. An updated search was performed on April 30, 2026, to capture the most recent literature (Table S4 in Multimedia Appendix 1).

### Selection Criteria and Data Extraction

The inclusion and exclusion criteria were established according to the Population, Intervention, Comparator, Outcome, and Study Design framework. We included the following definitions: telerehabilitation involves participants performing the prescribed exercises by watching training videos only, without any real-time AI guidance or feedback. TLRH-AI incorporates AI-enabled posture recognition, real-time movement correction, or instant scoring feedback. Specifically, during human–computer interaction, the AI identifies 30 body key points to detect movement accuracy and errors, provide real-time guidance, and automatically evaluate task completion (eg, graded as qualified, good, excellent, or outstanding). IPR: structured, face-to-face rehabilitation involving therapist-supervised exercise sessions with real-time guidance and correction. UC: basic health education and nonsystematic exercise advice without structured exercise programs or continuous professional supervision. In the final network, we treated telerehabilitation and TLRH-AI as 2 separate treatment nodes. We classified an intervention as telerehabilitation (non-AI) when rehabilitation exercises were delivered remotely (eg, video conferencing, apps with prerecorded exercise videos, or web-based programs) without AI-based functions. We classified an intervention as TLRH-AI only when the study explicitly reported AI features, such as motion recognition, automated feedback, or algorithm-based personalization/adaptation of the exercise program.

The follow-up time points at 4, 8, and 12 weeks were not selected in a results-driven manner; rather, they were prespecified based on the following criteria: (1) these time points were the most frequently reported follow-up assessments among the included studies, thereby maximizing comparability; (2) they correspond to commonly used and relatively consistent evaluation windows for rehabilitation effects in chronic low back pain research, namely short-term (≤4 weeks), short-to-midterm (8 weeks), and midterm (12 weeks); (3) to avoid double-counting highly correlated repeated measurements within the same study and artificially inflating its weight, we prespecified that only 1 assessment time point per study would be included within each time window, selecting the follow-up closest to the target window for analysis. A detailed description of the selection process is provided in Table S5 in Multimedia Appendix 1.

Two independent reviewers performed the study selection and data extraction. A third reviewer consolidated the results, and any discrepancies were resolved through discussion until consensus was reached. The following data were extracted into a standardized Microsoft Excel sheet: title, first author, publication year, study design, sample size, telerehabilitation technology used, intervention duration, follow-up period, and all reported clinical outcomes. Outcomes of primary interest were pain intensity, disability, kinesiophobia, and health-related quality of life.

### Risk of Bias Assessment

The methodological quality and risk of bias (RoB) of the included RCTs were assessed independently by 2 reviewers using the Cochrane Risk of Bias tool (2.0), as implemented in Review Manager (version 5.4.1) [21]. The evaluation covered the following domains: random sequence generation, allocation concealment, blinding of participants and personnel, blinding of outcome assessment, incomplete outcome data, selective reporting, and other potential sources of bias. Disagreements between reviewers were resolved through discussion or by consultation with a third reviewer when necessary. The overall RoB for each study was categorized as “low,” “some concerns,” or “high”. The RoB assessment was performed at the study level (not per outcome/per time point). We also corrected the figure caption to indicate that the RoB summary/graph refers to RCTs, not outcomes. The results of the assessment are presented graphically in the study.

### Data Analysis

Statistical analyses were performed within a Bayesian framework using R software (version 4.4.1) interfaced with JAGS (version 4.3.1). We used the default vague priors in *gemtc*: treatment effects followed Normal (0, [15 × om.scale]^2^), and heterogeneity was specified as τ ~ Uniform (0, om.scale) (τ² implied). In *gemtc*, om.scale is an internally determined scale parameter based on the observed variability and measurement scale of the outcome, and is used to set weakly informative prior ranges that are appropriate for the analyzed data. Markov Chain Monte Carlo (MCMC) sampling was used to jointly estimate direct and indirect treatment comparisons. A random-effects NMA model was constructed using the *gemtc* package, and the MCMC sampling was executed via the *rjags* package. The model was run with 4 chains, each with an initial adaptation phase of 50,000 iterations (discarded as burn-in), followed by 200,000 simulation iterations thinned by a factor of 10 to reduce autocorrelation [22]. We used the standardized mean difference (SMD) for pain intensity because the included trials measured pain using different scales, mainly the Visual Analogue Scale (VAS) and the Numerical Rating Scale) NRS, rather than a single uniform instrument. Using the SMD allows us to standardize effects across different pain scales and combine them within the NMA. Effect sizes were expressed as SMDs with 95% credible intervals (CrIs) for pain intensity, and as mean differences with 95% CIs for other continuous outcomes. Statistical heterogeneity was described using the *I*^2^ statistic for both pairwise and NMAs, with values greater than 50% considered suggestive of substantial heterogeneity. However, *I*^2^ was not used as the sole basis for interpreting heterogeneity because it does not directly quantify the absolute variation of true treatment effects across different populations or clinical settings [23]. Therefore, 95% prediction intervals (PIs) were additionally estimated for key network estimates where applicable to aid interpretation of the expected distribution of true effects in future comparable settings.

The 95% CrI was interpreted as the uncertainty around the average network treatment effect, whereas the 95% PI was interpreted as the expected range of true effects that may be observed in a future comparable clinical setting. SEs were derived from the reported 95% CrIs using the formula SE = (upper limit − lower limit) / 3.92. Estimated 95% PIs were then calculated as effect estimate ± 1.96 × √(SE^2^ + τ^2^), where τ represents the assumed between-study SD on the scale of each outcome. Because posterior estimates of τ were not consistently available for all network comparisons, τ values were specified according to the outcome scale, follow-up window, and evidence structure. These PIs were therefore interpreted as estimated predictive ranges rather than formal model-based posterior PIs [24]. When the average effect favored an intervention, but the corresponding PI was wide or crossed the null value, the finding was interpreted cautiously as potentially variable across future clinical settings. For outcomes where lower scores indicate improvement (pain, disability, and kinesiophobia), effect estimates were oriented so that positive values indicate improvement. Consistency between direct and indirect evidence was evaluated using the node-splitting approach; results were visualized using forest plots. Treatment rankings were inferred using the surface under the cumulative ranking curve (SUCRA) values, derived from the posterior probability matrix of each intervention being the most effective. Rank probability plots were generated to illustrate the likelihood of each treatment being ranked at each possible position. Network graphs and funnel plots were produced using Stata (version 16.0; StataCorp LLC). Small-study effects and funnel plot asymmetry were assessed using funnel plots and the Egger test. Funnel plot asymmetry was not interpreted as publication bias alone, because asymmetry may arise from several sources, including between-study heterogeneity, methodological differences, selective outcome reporting, chance, and other small-study effects [25].

### Assessment of Certainty of Evidence

The certainty of evidence for all estimated outcomes was assessed using the GRADE (Grading of Recommendations Assessment, Development, and Evaluation) framework for NMA [26]. This approach evaluates the certainty of the evidence for each pairwise comparison within the network separately, considering both direct and indirect evidence, and then rates the certainty of the NMA effect estimates. The assessment of certainty in effect estimates for direct comparison evidence includes the RoB, inconsistency, indirectness, imprecision, and other considerations. The assessment was performed independently by 2 reviewers, with any disagreements resolved by consensus or by consulting a third reviewer. The certainty was judged as high, moderate, low, or very low. The specific meanings are presented in Table S6 in Multimedia Appendix 1.

## Results

### Search Results and Study Characteristics

**Figure 1:**
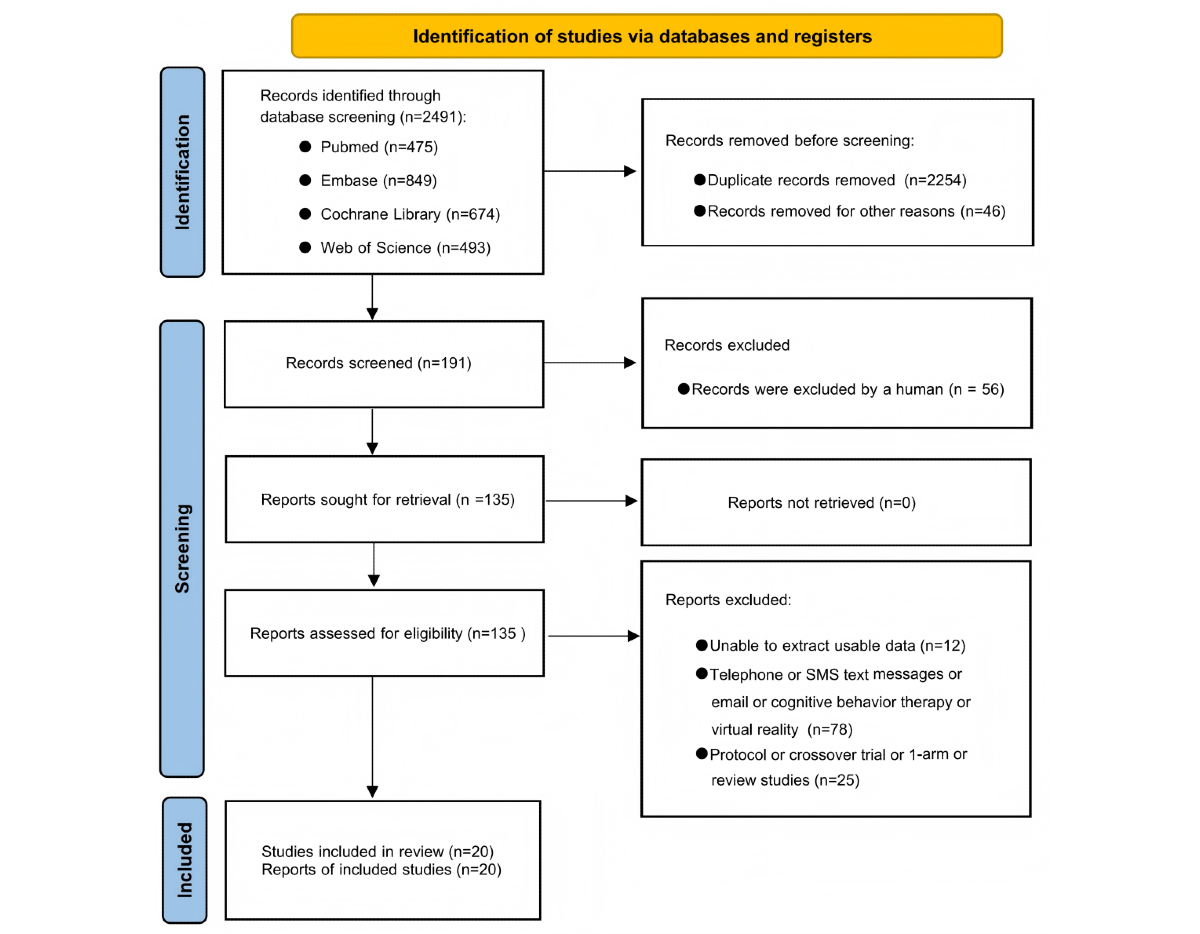
PRISMA (Preferred Reporting Items for Systematic Reviews and Meta-Analyses) flowchart for literature screening.

A total of 2491 articles were initially retrieved. Among them, 2254 duplicate records were excluded, and 46 articles were excluded during the preliminary screening. The remaining 2491 articles underwent full-text review. Finally, 171 studies did not meet the inclusion criteria and were excluded, leaving 20 RCTs [27-46] with a total of 1854 patients. The included interventions were TLRH-AI, telerehabilitation, IPR, and UC. Study numbers and total sample size in each network comparison are provided in Table S7 in Multimedia Appendix 1. We excluded interventions based on phone or text support, nonexercise virtual reality, online self-management or cognitive behavioral programs without specified exercise content, online consultations without guided exercise, and wearable sensors or applications used only for tracking without exercise guidance. The distribution of major clinical and methodological characteristics across intervention nodes was considered broadly comparable, supporting the plausibility of the transitivity assumption (Table S8 in Multimedia Appendix 1). The estimated 95% PIs for the key network are shown in Table S9 in Multimedia Appendix 1. The PRISMA flow diagram of the literature search is presented in Figure 1.

Telerehabilitation technologies primarily include smartphone apps (providing instructional videos or AI-assisted guidance), video conferencing, and exercise tutorials based on computer or web platforms. The intervention period ranged from 4 to 12 weeks. The control group typically received traditional outpatient physiotherapy or paper-based conventional physiotherapy. Pain intensity was measured using the VAS or the NRS; disability or functional status was assessed using the Oswestry Disability Index (ODI) or the Roland-Morris Disability Questionnaire (RMDQ); fear of movement was evaluated using the Tampa Scale for Kinesiophobia (TSK); and health-related quality of life was measured with the 12-item Short-Form Health Survey (SF-12), including the physical component summary (PCS) and the mental component summary (MCS). Table 1 lists the basic characteristics of the included studies.

**Table 1:** The characteristics of the included studies. Among the 20 included studies, 19 were 2-arm trials and 1 was a 3-arm trial; no single-arm studies were identified.

Study; year; design	Sample size	Telerehabilitation technology	Telerehabilitation intervention	Control group	Follow-up period
Xiao et al [27]; 2025; RCT^a^	Telerehabilitation: n=18 Control group: n=16	Smartphone and tablet app add-in AI^b^-assisted guidance	4 weeks, 3 times per week, 30‐45 minutes per session, core, flexibility, Mackenzie, breathing exercises, and education	Watching a training video, the same exercises, and education	4 weeks
Karaduman et al^c^ [28]; 2024; RCT	Telerehabilitation: n=22 Control group 1: n=22 Control group 2: n=22	Video conference	4 weeks, 3 times per week, and 20-30 minutes per session Core exercise and education	Control group 1: exercises at the hospital; Control group 2: conventional physiotherapy	4 weeks
Park et al [29]; 2023; RCT	Telerehabilitation: n=50 Control group: n=50	“Dr AI” platform AI-assisted guidance	4 weeks, 3 times per week, 30 minutes per session, exercise, and education	Physiotherapy clinic	4 weeks
Almhdawi et al [30]; 2020; RCT	Telerehabilitation: n=21 Control group: n=20	Smartphone app Instructional videos	6 weeks, exercise, 4 phone notifications, and education	Conventional physiotherapy and education	6 weeks
Villatoro-Luque et al [31]; 2023; RCT	Telerehabilitation: n=36 Control group: n=35	Computer platform Instructional videos	8 weeks, 2 times per week, and 6 exercises per session	Physiotherapy clinic	8 weeks
Shi et al [32]; 2024; RCT	Telerehabilitation: n=27 Control group: n=27	Smartphone app Instructional videos	8 weeks, 3 times per week, and 30 minutes for exercises, and education	Physiotherapy clinic	8 weeks
Villatoro-Luque et al [33]; 2025; RCT	Telerehabilitation: n=34 Control group: n=34	Videoconference (WhatsApp)	8 weeks, 2 times per week, 30 minutes for exercises, and education	Physiotherapy clinic	8 weeks
Ozden et al [34]; 2022; RCT	Telerehabilitation: n=25 Control group: n=25	Web-based platform Video exercise	8 weeks, 1 time per day, core, flexibility, Mackenzie exercises, and education	Paper-based conventional physiotherapy and education	8 weeks
Feng et al [35]; 2025; RCT	Telerehabilitation: n=39 Control group: n=39	Smartphone app Instructional videos	8 weeks, 3 times per week, 40 minutes for exercises, and education	Paper-based conventional physiotherapy and education	8 weeks
Fatoye et al [36]; 2020; RCT	Telerehabilitation: n=21 Control group: N=26	Smartphone app Instructional videos	8 weeks, 3 times per week, and Mackenzie exercises	Clinic-based McKenzie therapy	8 weeks
Mbada et al [37]; 2019; RCT	Telerehabilitation: n=21 Control group: n=26	Smartphone app Instructional videos	8 weeks, 3 times per week, and Mackenzie exercises	Clinic-based McKenzie therapy	8 weeks
Zadro et al [38]; 2019; RCT	Telerehabilitation: n=30 Control group: n=30	Video game Wii Fit U software	8 weeks, 3 times per week, 60 minutes per session, flexibility, strength, aerobic training, and education	Conventional physiotherapy and education	8 weeks
Lara-Palomo et al [39]; 2022; RCT	Telerehabilitation: n=39 Control group: n=35	An internet-based system Using the McKenzie Method Videos	8 weeks, 3 times per week, and McKenzie exercises	Self-treatment McKenzie approach for home	8 weeks
Toelle et al [40]; 2019; RCT	Telerehabilitation: n=42 Control group: n=42	Smartphone and tablet app Instructional videos	12 weeks, 4 times per week, physical exercise, and education	Physiotherapy clinic	12 weeks
Shebib et al [41]; 2019; RCT	Telerehabilitation: n=113 Control group: n=64	Tablet app Instructional videos	12 weeks, 3 times per week, physical exercise, and education	Conventional physiotherapy and education	12 weeks
Koppenaal et al [42]; 2022; RCT	Telerehabilitation: n=102 Control group: n=102	Smartphone app Instructional videos	12-week tailored exercises	Face-to-face physiotherapy and education	12 weeks
Geraghty et al [43]; 2018; RCT	Telerehabilitation: n=27 Control group: n=27	“SupportBack” system	6 weeks, 1 time per week, exercises or walking, and education	Conventional physiotherapy and education	12 weeks
Özden et al [44]; 2024; RCT	Telerehabilitation: n=22 Control group: n=22	“PhysioAnalyst” app	8 weeks, 2 times per week, stretching, strengthening, and core stabilization exercises	Conventional physiotherapy	12 weeks
Santos et al [45]; 2024; RCT	Telerehabilitation: n=20 Control group: n=20	“PAT-Back” program	8 weeks, 3-5 times per week, pain education (20 minutes), and supervised exercise therapy (60 minutes)	Conventional physiotherapy and education	12 weeks
Sandal et al [46]; 2021; RCT	Telerehabilitation: n=232 Control group: n=229	“SELFBACK” support system	8 weeks, physical activity, strength and flexibility exercises, and education	Conventional physiotherapy and education	9 months

^a^RCT: randomized controlled trial.

^b^AI: artificial intelligence.

^c^This study was a 3-arm trial.

### Quality Assessment

**Figure 2:**
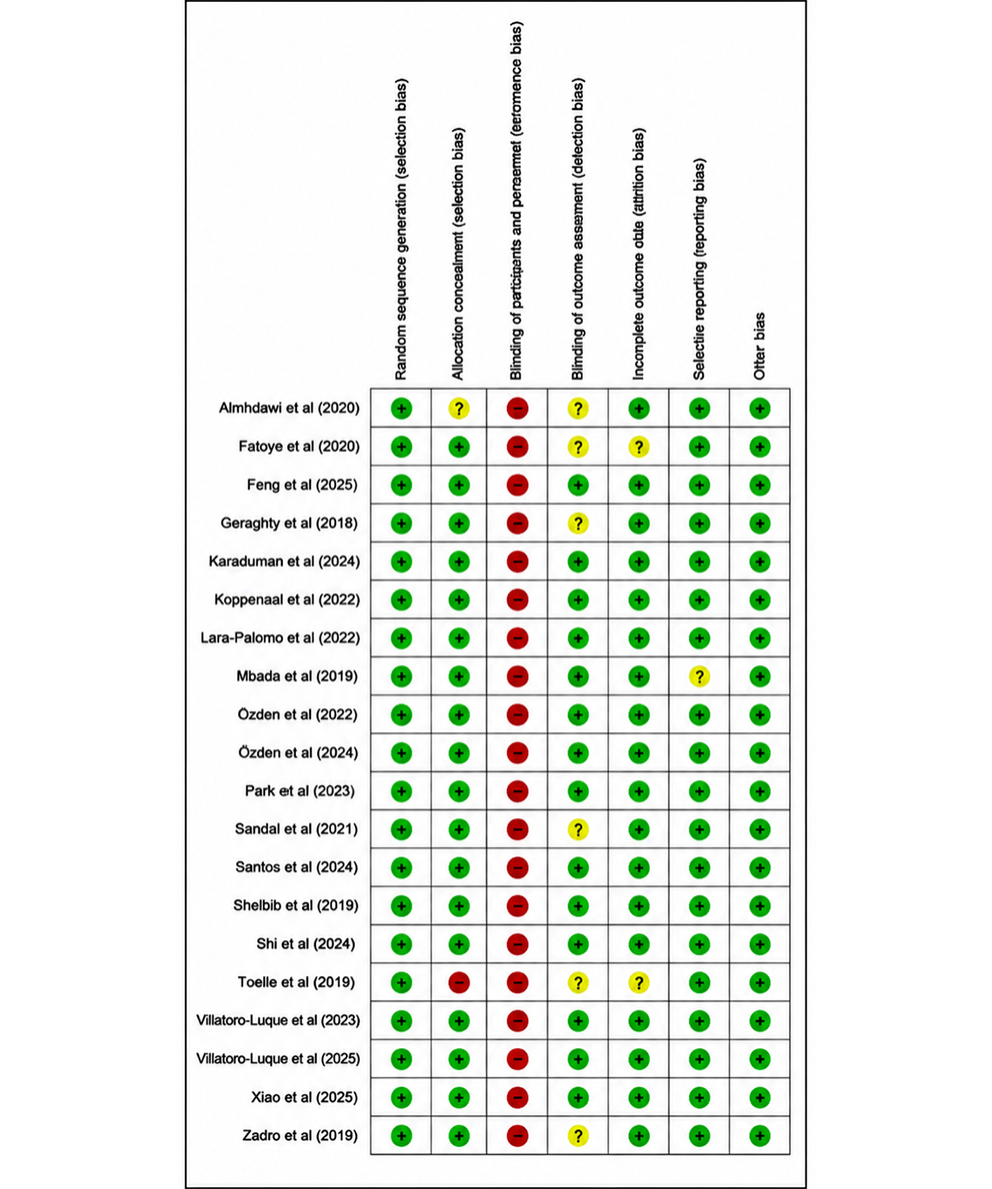
Risk-of-bias summary and graph showing the authors’ judgments for each risk-of-bias domain across randomized controlled trials. The risk-of-bias assessment refers to studies rather than outcomes.

The overall methodological quality of the included studies is illustrated in Figure 2 and Figure S1 in Multimedia Appendix 1. Most studies described random sequence generation and allocation concealment methods adequately, demonstrating a low risk of selection bias. Regarding blinding of participants and personnel, all studies were at high risk. This was due to the nature of the interventions: telerehabilitation, such as video guidance, AI-assisted training, or app-based guidance, differed significantly in implementation from IPR or UC, making blinding of participants and therapists practically infeasible.

In terms of blinding of outcome assessment, some studies explicitly reported the use of blinding for outcome assessment; for example, data were collected by researchers unaware of group allocation, and these studies were rated as low risk. Studies that did not report assessor blinding were rated as having an unclear risk. For incomplete outcome data and selective reporting, most studies had low dropout rates and reported prespecified outcome measures, thus being rated as low risk.

### Results of Bayesian NMA

#### Pain Intensity

**Figure 3:**
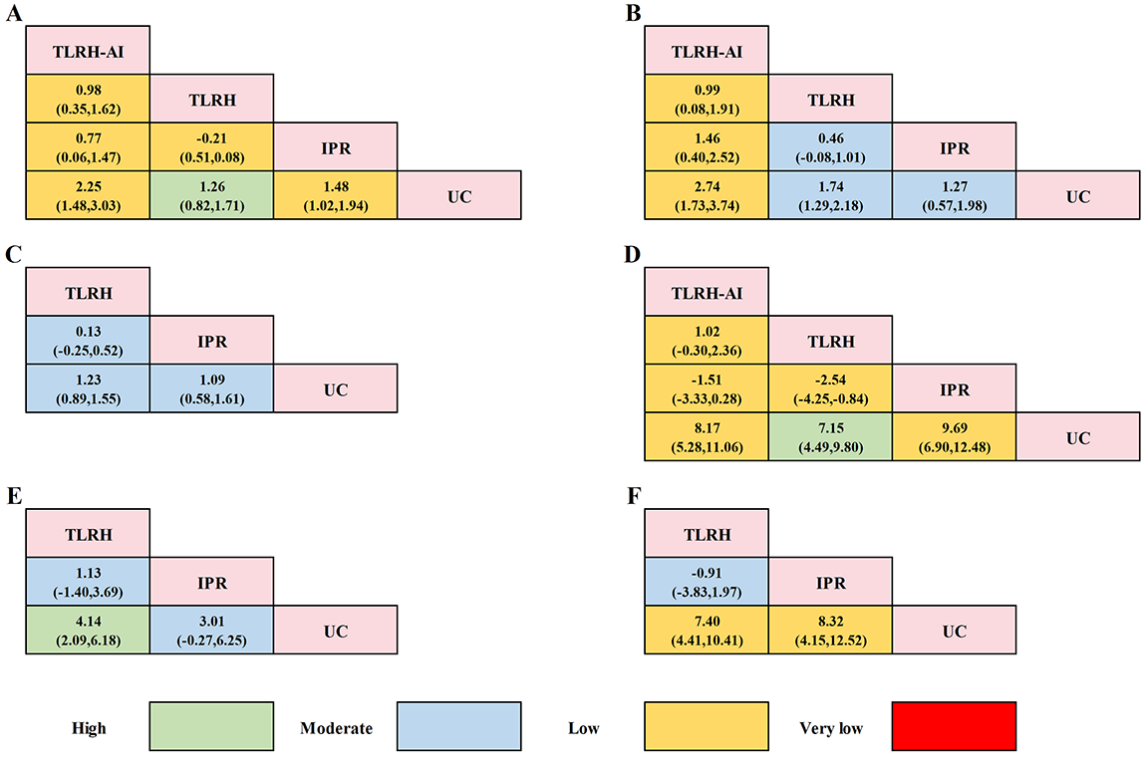
League matrix. (A) Effects of 4 rehabilitation interventions on pain intensity in patients with chronic nonspecific low back pain (CNSLBP). (B) Effects of 4 rehabilitation interventions on pain intensity at 8 weeks after the intervention in CNSLBP. (C) Effects of 3 rehabilitation interventions on pain intensity at 12 weeks after the intervention in CNSLBP. (D) Effects of 4 rehabilitation interventions on disability in CNSLBP. (E) Effects of 3 rehabilitation interventions on disability at 8 weeks after the intervention in CNSLBP. (F) Effects of 3 rehabilitation interventions on disability at 12 weeks after the intervention in CNSLBP. IPR: in-person rehabilitation; TLRH: telerehabilitation; TLRH-AI: telerehabilitation combined with artificial intelligence; UC: usual care.

This study assessed pain intensity using the VAS and the NRS at postintervention time points of 4, 8, and 12 weeks. Six studies [27,28,30,32,34,37] reported pain intensity at 4 weeks postintervention. Comparative network estimates are presented in Figure 3A. Compared with UC, IPR significantly reduced pain intensity (SMD 1.48, 95% CrI 1.02-1.94, 95% PI 0.65-2.31); the estimated PI remains above the null value, favoring IPR. Interpretation should remain cautious because certainty is low.

Nine studies [27,31,32,34,35,37-39,45] reported pain intensity at 8 weeks postintervention. Comparative network estimates are presented in Figure 3B. Compared with UC, telerehabilitation significantly reduced pain intensity (SMD 1.74, 95% CrI 1.29-2.18, 95% PI 1.01-2.47); the estimated PI remains above the null value, suggesting a relatively stable pain benefit of telerehabilitation across comparable settings. With moderate-certainty evidence. Evidence on TLRH-AI was limited and is summarized descriptively only. Although available studies suggested possible short-term pain benefits at 4 and 8 weeks, the certainty of the evidence was low and insufficient to support reliable comparative conclusions.

**Figure 4:**
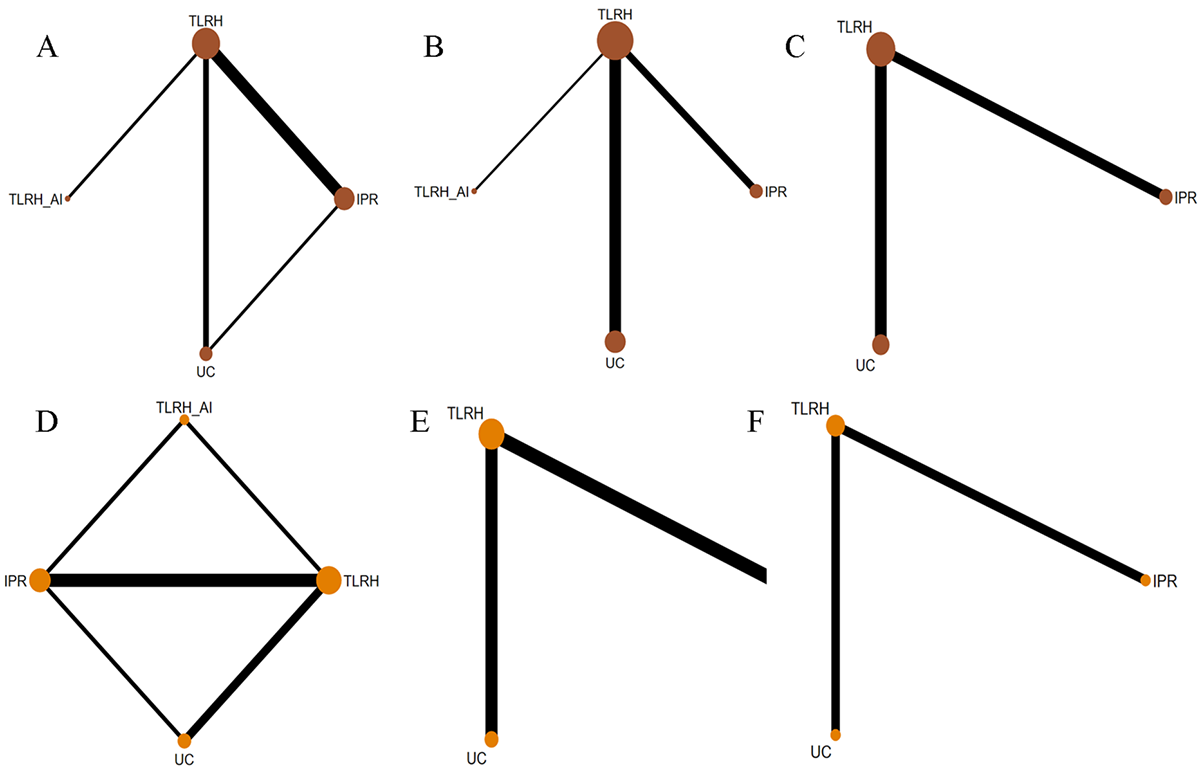
Network diagram. (A) Effects of 4 rehabilitation interventions on pain intensity in patients with chronic nonspecific low back pain (CNSLBP). (B) Effects of 4 rehabilitation interventions on pain intensity at 8 weeks after the intervention in CNSLBP. (C) Effects of 3 rehabilitation interventions on pain intensity at 12 weeks after the intervention in CNSLBP. (D) Effects of 4 rehabilitation interventions on disability in CNSLBP. (E) Effects of 3 rehabilitation interventions on disability at 8 weeks after the intervention in CNSLBP. (F) Effects of 3 rehabilitation interventions on disability at 12 weeks after the intervention in CNSLBP. Telerehabilitation combined with artificial intelligence (TLRH-AI) node based primarily on a single study (n=34); findings are exploratory only. IPR: in-person rehabilitation; TLRH: telerehabilitation; UC: usual care.

Seven studies [31,40-44,46] reported pain intensity at 12 weeks postintervention, and the league matrix (Figure 3C) was used to describe the effects of 3 interventions on pain intensity, with 3 comparisons in total. Comparing UC and telerehabilitation resulted in an SMD of 1.23 (95% CrI 0.89-1.55, 95% PI 0.64-1.82); the estimated PI remains above the null value, supporting longer-term pain relief with telerehabilitation in comparable settings. IPR (SMD 1.09, 95% CrI 0.58-1.61, 95% PI 0.37-1.81) significantly reduced pain intensity, with statistically significant differences; the estimated PI favors IPR, although the interval is wider, indicating possible between-setting variability. The certainty of the evidence was moderate for both. The SUCRA ranking plot showed that telerehabilitation (SUCRA 87.2%) was the most effective. The network diagram is shown in Figures 4A-C, and the SUCRA ranking plot is shown in Figure S2 in Multimedia Appendix 1.

#### Disability ODI

The assessment time points were 4, 8, and 12 weeks postintervention. Six studies [27-29,32,34,36] reported disability at 4 weeks postintervention. A league matrix (Figure 3D) was used to describe the effects of 4 interventions on disability, with a total of 6 comparisons. Compared with UC, IPR (mean difference [MD] 9.69, 95% CrI 6.90-12.48, 95% PI 2.29-17.09) significantly reduced disability, with statistically significant differences. PI favors IPR, but the range is wide, and certainty is low; the magnitude of benefit may vary across settings. However, the certainty of the evidence was low.

Evidence on TLRH-AI at 4 weeks was limited and is therefore presented descriptively only. Available studies suggested possible short-term benefits for disability improvement; however, the certainty of the evidence was low, and the available data were insufficient to support a reliable comparative synthesis. The SUCRA ranking plot showed that IPR (SUCRA 98.2%) was the most effective.

Six studies [31,32,34,36,39,45] reported disability at 8 weeks postintervention. The league matrix (Figure 3E) was used to describe the effects of 3 interventions on disability, with 3 comparisons in total. Compared with UC, telerehabilitation (MD 4.14, 95% CrI 2.09-6.18, 95% PI –1.37 to 9.65) significantly reduced disability, with a statistically significant difference. Average effect favors telerehabilitation, but the estimated PI crosses the null value, indicating that functional benefit may be less stable in future settings. The certainty of the evidence was high for telerehabilitation and moderate for the other comparisons. The SUCRA ranking plot showed that telerehabilitation (SUCRA 90.4%) was the most effective.

Four studies [30,40,41,43] reported disability at 12 weeks postintervention. The league matrix (Figure 3F) was used to describe the effects of 3 interventions on disability, with 3 comparisons in total. Compared with UC, IPR (MD 8.32, 95% CrI 4.15-12.52, 95% PI 0.33-16.31) significantly reduced disability, with statistically significant differences. PI favors IPR, but the wide interval suggests substantial between-setting variability. However, the certainty of the evidence was low. The SUCRA ranking plot showed that IPR (SUCRA 86.7%) was the most effective. The network diagram is shown in Figures 4D-F, and the SUCRA ranking plot is shown in Figure S2 in Multimedia Appendix 1.

#### Disability RMDQ

**Figure 5:**
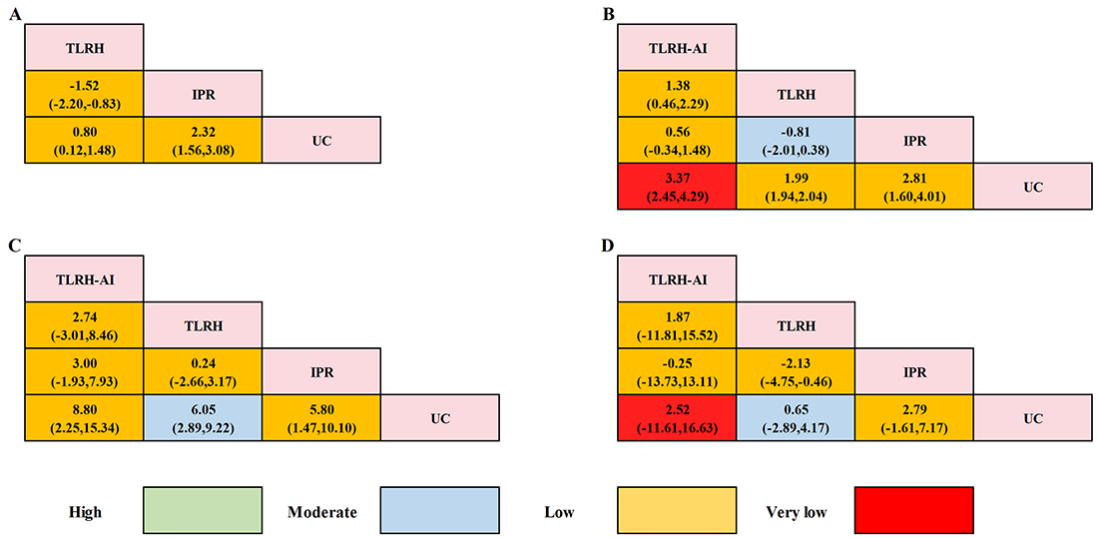
League matrix. (A) Effects of 3 rehabilitation interventions on kinesiophobia in patients with chronic nonspecific low back pain (CNSLBP). (B) Effects of 4 rehabilitation interventions on Roland-Morris Disability Questionnaire (RMDQ) scores in CNSLBP. (C) Effects of 4 rehabilitation interventions on the physical component summary (PCS) of health-related quality of life in CNSLBP. (D) Effects of 4 rehabilitation interventions on the mental component summary (MCS) of health-related quality of life in CNSLBP. Telerehabilitation combined with artificial intelligence (TLRH-AI) node based primarily on a single study (n=34); findings are exploratory only. IPR: in-person rehabilitation; TLRH: telerehabilitation; UC: usual care.

Seven studies [27,29,35,37,38,43,46] assessed disability using the RMDQ. The league matrix (Figure 5B) was used to describe the effects of 4 interventions on disability, with a total of 6 comparisons. Compared with UC, IPR (MD 2.81, 95% CrI 1.60-4.01, 95% PI 0.49-5.13) significantly reduced disability, with statistically significant differences. PI favors IPR, but certainty is low, and the magnitude of effect may vary.

**Figure 6:**
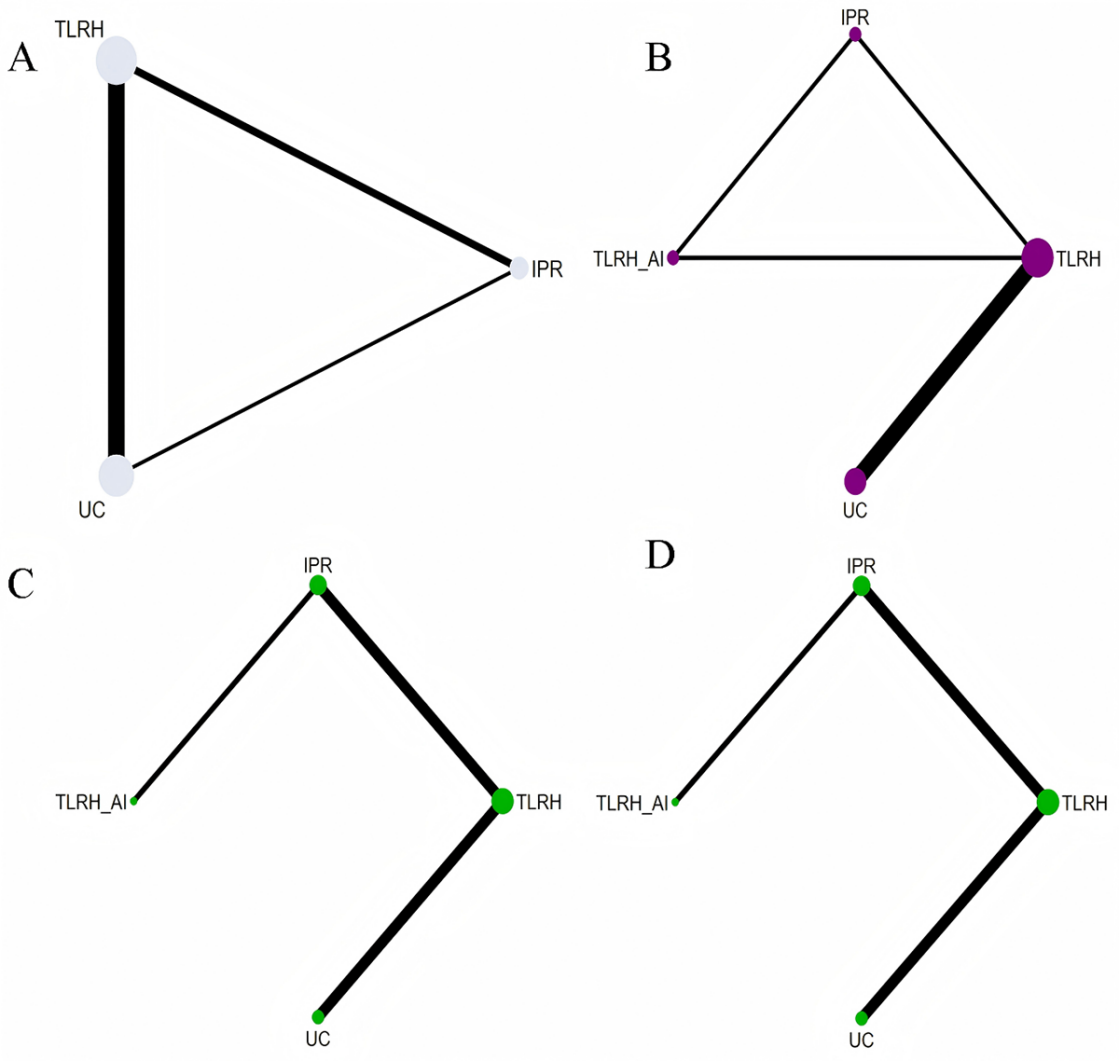
Network diagram. (A) Effects of 3 rehabilitation interventions on kinesiophobia in patients with chronic nonspecific low back pain (CNSLBP). (B) Effects of 4 rehabilitation interventions on Roland-Morris Disability Questionnaire (RMDQ) scores in CNSLBP. (C) Effects of 4 rehabilitation interventions on the physical component summary of health-related quality of life in CNSLBP. (D) Effects of 4 rehabilitation interventions on the mental component summary of health-related quality of life in CNSLBP. Telerehabilitation combined with artificial intelligence (TLRH-AI) node based primarily on a single study (n=34); findings are exploratory only. IPR: in-person rehabilitation; TLRH: telerehabilitation; UC: usual care.

Evidence on TLRH-AI was limited and is therefore presented descriptively only. Available studies suggested possible benefits for disability improvement; however, the certainty of the evidence was low, and the available data were insufficient to support a reliable comparative synthesis. The SUCRA ranking plot showed that IPR (SUCRA 67.3%) was among the more effective interventions. The network diagram is shown in Figure 6B, and the SUCRA ranking plot is shown in Figure S2 in Multimedia Appendix 1.

#### Kinesiophobia Assessed Using the TSK

Six studies [28,33,34,38,39,43] assessed kinesiophobia using the TSK. The league matrix (Figure 5A) was used to describe the effects of 3 interventions on kinesiophobia, with a total of 3 comparisons. Compared with UC, IPR (MD 2.32, 95% CrI 1.56-3.08, 95% PI –0.70 to 5.34) significantly reduced kinesiophobia, with a statistically significant difference. Average effect favors IPR, but the estimated PI crosses the null value; effects on kinesiophobia may not be stable across future settings. However, the certainty of the evidence was low. The SUCRA ranking plot showed that IPR (SUCRA 99%) was the most effective. The network diagram is shown in Figure 6A, and the SUCRA ranking plot is shown in Figure S2 in Multimedia Appendix 1.

#### Health-Related Quality of Life

The SF-12 was used to measure health-related quality of life, including the PCS and MCS. The PCS was included in 5 studies [29,30,35,37,40]. The league matrix (Figure 5C) was used to describe the effects of 4 interventions on quality of life, with a total of 6 comparisons. Compared with UC, telerehabilitation significantly improved PCS (MD 6.05, 95% CrI 2.89-9.22, 95% PI 0.36-11.74). PI favors telerehabilitation, suggesting possible improvement in physical quality of life, although the predictive range remains wide. The certainty of the evidence was moderate. Evidence on TLRH-AI for PCS was limited and is therefore presented descriptively only. Available studies suggested possible benefits for the physical component of health-related quality of life; however, the certainty of the evidence was very low, and the available data were insufficient to support a reliable comparative synthesis. The MCS was included in 5 studies [29,30,35,37,41]. The league matrix (Figure 5D) was used to describe the effects of 4 interventions on quality of life, with a total of 6 comparisons. Compared with UC, IPR (MD 2.79, 95% CrI −1.61 to 7.17, 95% PI −4.57 to 10.15) improved quality of life, but the difference was not statistically significant. Both the average effect estimate and the estimated PI were compatible with no effect; no stable benefit in mental quality of life can be inferred. The certainty of the evidence was low. Evidence on TLRH-AI for MCS was also limited and is presented descriptively only. Available studies suggested possible benefits; however, the certainty of the evidence was low, and no reliable comparative conclusions could be drawn. The network diagrams are shown in Figures 6C-D, and the SUCRA ranking plot is shown in Figure S2 in Multimedia Appendix 1.

#### Publication Bias and Test for Inconsistency

Both visual inspection of the funnel plot and statistical evaluation using the Egger test indicated no significant evidence of publication bias (Egger test *P*>.05; Figure S3 in Multimedia Appendix 1). The symmetrical distribution of effect sizes in the funnel plot further supported this conclusion. Furthermore, statistical tests for inconsistency were not significant (all *P*>.05).

### Certainty of Evidence

This study systematically assessed the certainty of the evidence between different comparison groups using the GRADE framework. In addition, the league matrix tables visually highlight the certainty of the effect estimates using different colors. As shown in all league matrix tables, the certainty of the evidence for all outcomes was rated from very low to high according to GRADE.

Available studies suggested possible benefits of TLRH-AI for reducing pain intensity; however, the certainty of the evidence was low, and these findings were insufficient to support reliable comparative conclusions. In contrast, the certainty of evidence for TLRH at 4, 8, and 12 weeks was rated as high or moderate. IPR demonstrated a strong statistical effect in reducing the primary outcome of disability, but the certainty of the evidence was rated as low. To improve the transparency of the methodology, Table 2 provides a detailed explanation of the reasons for evidence downgrading.

**Table 2:** Specific grounds for downgrading the quality of evidence in the GRADE (Grading of Recommendations Assessment, Development, and Evaluation) framework. Risk of bias was explicitly considered for all GRADE ratings, and additional downgrading was applied only when bias was judged likely to materially affect the GRADE reliability of the corresponding network estimate.

Outcome time point and treatment comparison			Direct estimate					Indirect estimate					Network estimate		
			MD^a^ (95% CrI^b^)		Certainty		MD (95% CrI)			Certainty		MD (95% CrI)			Certainty
Pain intensity in the 4-week intervention															
	TLRH-AI^c^ vs TLRH^d^	0.99(−0.49 to 2.4)		Low		—^e^			Very low		0.9881 (0.3516 to 1.6214)			Low	
	TLRH-AI vs IPR^f^	—		—		—			Low		0.7731 (0.0672 to 1.4757)			Low	
	TLRH-AI vs UC^g^	—		—		—			Low		2.2584 (1.4836 to 3.0302)			Low	
	TLRH vs IPR	−0.090 (−0.82 to 0.72)		Low		—			Low		−0.2166 (−0.5198 to 0.0882)			Low	
	TLRH vs UC	1.1 (0.033 to 2.2)		High		—			Low		1.2687 (0.8226 to 1.713)			High	
	IPR vs UC	1.7(0.26 to 3.2)		Low		0.91 (−0.92 to 2.7)			Low		1.4867 (1.0247 to 1.9458)			Low	
Pain intensity in the 8-week intervention															
	TLRH-AI vs TLRH	1.0 (−0.77 to 2.8)		Low		—			Very low		0.9997 (0.0871 to 1.913)			Low	
	TLRH-AI vs IPR	—		—		—			Low		1.4609 (0.4013 to 2.5277)			Low	
	TLRH-AI vs UC	—		—		—			Low		2.7428 (1.7315 to 3.7491)			Low	
	TLRH vs IPR	0.45 (−0.62 to 1.5)		Moderate		—			Very low		0.4628 (−0.0799 to 1.0014)			Moderate	
	TLRH vs UC	1.7 (0.89 to 2.5)		Moderate		—			Very low		1.7407 (1.2983 to 2.1828)			Moderate	
	IPR vs UC	—		—		—			Moderate		1.2785 (0.5766 to 1.9803)			Moderate	
Pain intensity in the 12-week intervention															
	TLRH vs IPR	0.12 (−1.9 to 2.1)		Moderate		—			Very low		0.1319 (−0.2564 to 0.5265)			Moderate	
	TLRH vs UC	2.0 (0.31 to 3.8)		Moderate		—			Very low		1.2282 (0.8994 to 1.5562)			Moderate	
	IPR vs UC	—		—		—			Moderate		1.0964 (0.5845 to 1.606)			Moderate	
ODI^h^ in the 4-week intervention															
	TLRH-AI vs TLRH	1.4 (−12.0 to 15.0)		Low		−1.1 (−15.0 to 13.0)			Low		1.029 (−0.3055 to 2.3607)			Low	
	TLRH-AI vs IPR	−2.7 (−17.0 to 11.0)		Low		−0.14 (−14.0 to 13.0)			Low		−1.5174 (−3.3336 to 0.2856)			Low	
	TLRH-AI vs UC	—		—		—			Low		8.1763 (5.289 to 11.0632)			Low	
	TLRH vs IPR	−0.47 (−8.9 to 7.9)		Low		−3.8 (−16.0 to 8.6)			Low		−2.5454 (−4.25 to −0.8434)			Low	
	TLRH vs UC	4.8 (−6.0 to 15.0)		Moderate		—			Low		7.1503 (4.4979 to 9.8057)			Moderate	
	IPR vs UC	13.0 (−1.8 to 27.0)		Low		4.2 (−9.9 to 18.0)			Low		9.6929 (6.9022 to 12.4809)			Low	
ODI in the 8-week intervention															
	TLRH vs IPR	1.1 (−2.6 to 4.7)		Moderate		—			Very low		1.1372 (−1.4099 to 3.691)			Moderate	
	TLRH vs UC	4.3 (1.1 to 7.9)		High		—			Very low		4.1416 (2.0924 to 6.1848)			High	
	IPR vs UC	—		—		—			Moderate		3.0058 (−0.2711 to 6.2554)			Moderate	
ODI in the 12-week intervention															
	TLRH vs IPR	−1.4 (−18.0 to 15.0)		Moderate		—			Very low		−0.9189 (−3.8351 to 1.9752)			Moderate	
	TLRH vs UC	11.0 (−5.1 to 28.0)		Low		—			Very low		7.4084 (4.4119 to 10.4138)			Low	
	IPR vs UC	—		—		—			Low		8.3259 (4.1524 to 12.5208)			Low	
Roland-Morris Disability Questionnaire															
	TLRH-AI vs TLRH	1.3 (−1.2 to 3.8)		Low		1.7 (−1.5 to 4.8)			Low		1.3828 (0.4642 to 2.2996)			Low	
	TLRH-AI vs IPR	0.67 (−1.9 to 3.2)		Low		0.27 (−2.9 to 3.4)			Low		0.5669 (−0.3441 to 1.483)			Low	
	TLRH-AI vs UC	—		—		—			Low		3.3777 (2.4592 to 4.2959)			Low	
	TLRH vs IPR	–1.4 (–4.7 to 1.9)		Low		−0.55 (−3.6 to 2.5)			Low		−0.8152 (−2.0152 to 0.3882)			Low	
	TLRH vs UC	1.4 (–0.024 to 2.7)		Moderate		—			Very low		1.9944 (1.9409 to 2.0476)			Moderate	
	IPR vs UC	—		—		—			Low		2.809 (1.605 to 4.0121)			Low	
Tampa Scale for Kinesiophobia															
	TLRH vs IPR	0.13 (−6.4 to 6.8)		Low		—			Low		–1.5213 (–2.2039 to –0.8387)			Low	
	TLRH vs UC	2.7 (–1.5 to 7.2)		Low		—			Low		0.8021 (0.1209 to 1.4863)			Low	
	IPR vs UC	2.3 (–6.9 to 11.0)		Low		2.4 (–7.1 to 12.0)			Low		2.3243 (1.5673 to 3.0826)			Low	
SF-12^i^ (physical component summary)															
	TLRH-AI vs TLRH	—		—		—			Low		2.7457 (–3.0104 to 8.4688)			Low	
	TLRH-AI vs IPR	3.0 (−17.0 to 23.0)		Low		—			Very low		3.004 (–1.9306 to 7.9341)			Low	
	TLRH-AI vs UC	—		—		—			Very low		8.8025 (2.2586 to 15.3445)			Very low	
	TLRH vs IPR	–0.36 (−15.0 to 14.0)		Moderate		—			Very low		0.249 (–2.6605 to 3.1789)			Moderate	
	TLRH vs UC	9.1 (–4.0 to 25.0)		Low		—			Very low		6.0539 (2.8955 to 9.2264)			Low	
	IPR vs UC	—		—		—			Low		5.8096 (1.4726 to 10.1017)			Low	
SF-12 (mental component summary)															
	TLRH-AI vs TLRH	—		—		—			Low		1.8723 (–11.8187 to 15.5252)			Low	
	TLRH-AI vs IPR	–0.23 (−15.0 to 14.0)		Low		—			Very low		–0.2539 (–13.7345 to 13.1109)			Low	
	TLRH-AI vs UC	—		—		—			Very low		2.5279 (–11.6121 to 16.6353)			Very low	
	TLRH vs IPR	–2.3 (–7.2 to 2.4)		Low		—			Very low		–2.1348 (–4.7555 to 0.4623)			Low	
	TLRH vs UC	0.60 (–4.9 to 6.0)		Moderate		—			Very low		0.6559 (–2.893 to 4.178)			Moderate	
	IPR vs UC	—		—		—			Low		2.7973 (–1.6177 to 7.1722)			Low	

^a^MD: mean difference.

^b^CrI: credible interval.

^c^TLRH: telerehabilitation.

^d^TLRH-AI: telerehabilitation combined with artificial intelligence.

^e^Not applicable.

^f^IPR: in-person rehabilitation.

^g^UC: usual care.

^h^ODI: Oswestry Disability Index.

^i^SF-12: 12-item Short-Form Health Survey.

### Consistency Assessment

We performed node-splitting consistency tests for comparisons in the network that had both direct and indirect evidence. For the 4-week pain intensity outcome, only UC vs IPR could be evaluated, with a direct effect MD of −1.720 (95% CrI −2.239 to −1.204) and an indirect effect MD of −0.983 (95% CrI −2.032 to 0.061), with a difference of approximately 0.74 between the two. The inconsistency test showed no significant difference (*P*=.22).

For the 4-week ODI outcome, evaluable comparisons included telerehabilitation vs TLRH-AI, IPR vs TLRH-AI, IPR vs TLRH, and UC vs IPR. Node-splitting tests for all comparisons did not reach statistical significance (*P*=.19, *P*=.19, *P*=.17, and *P*=.05, respectively). Among them, the direct effect for UC vs IPR was −12.63 (95% CrI −15.93 to −9.345) and the indirect effect was −3.715 (95% CrI −12.16 to 4.665), with a difference of approximately 8.92, representing a potentially important inconsistency, even though its statistical test did not reach significance.

For the remaining comparisons, differences between direct and indirect estimates were approximately 2.42 to 2.74. For the TSK outcome, only UC vs IPR could be evaluated, with a direct effect of −2.269 (95% CrI −3.052 to −1.481) and an indirect effect of −0.662 (95% CrI −3.726 to 2.388), with a difference of approximately 1.61. The inconsistency test showed no significant difference (*P*=.32). For the RMDQ outcome, evaluable comparisons included telerehabilitation vs TLRH-AI, IPR vs TLRH-AI, and IPR vs TLRH, with *P*=.60, *P*=.61, and *P*=.60, respectively, showing no statistically significant inconsistency. Differences between direct and indirect estimates were approximately 0.74, 0.72, and 0.73, respectively, suggesting generally good consistency within this outcome. Overall, node-splitting tests for all evaluable comparisons did not reveal statistically significant inconsistency, suggesting acceptable overall network consistency. However, in some comparisons, particularly UC vs IPR for the 4-week ODI outcome, a potentially important deviation between direct and indirect estimates was observed. Therefore, these network estimates should be interpreted cautiously, especially considering the limited statistical power, the quantity of available evidence, and the width of the CrIs.

## Discussion

### Overview

This Bayesian NMA compared the relative effects of TLRH-AI, telerehabilitation, IPR, and UC in patients with CNSLBP. The main findings were that supervised exercise-based interventions were generally associated with better outcomes than UC. Telerehabilitation showed the most favorable effect on pain reduction at 12 weeks and improved the physical component of health-related quality of life, whereas IPR appeared to provide greater benefits for disability and kinesiophobia. Findings related to TLRH-AI were exploratory only and, because of the low certainty of evidence, were insufficient to support reliable comparative conclusions. Overall, these findings support an individualized rehabilitation strategy according to patients’ pain, functional, and psychological recovery needs.

Our findings showed that, compared with UC, all supervised exercise-based interventions were associated with significant improvements in pain and disability [4-6,47]. This result is consistent with previous evidence supporting exercise-based management as a core component of CNSLBP care, not merely a simple alternative or an inferior option. Importantly, telerehabilitation appeared to achieve outcomes comparable to those of conventional outpatient rehabilitation in pain relief and functional improvement, suggesting that remote delivery of structured exercise programs may represent a feasible and effective alternative to IRP in selected populations. Previous studies have similarly reported no significant differences between app-based remote exercise training and traditional outpatient exercise therapy in terms of pain intensity and ODI improvement, further supporting the clinical utility of remotely supervised rehabilitation [48].

The relatively sustained 12-week benefit of telerehabilitation observed in this study may reflect mechanisms that are particularly relevant to chronic pain self-management, including repeated home-based practice, lower access barriers, and better integration of exercise into daily routines. This interpretation is also supported by recent clinical studies of exercise-based telerehabilitation in chronic low back pain, which have reported meaningful improvements in pain and related outcomes after structured remote programs [33]. By contrast, the short-term benefit observed with IPR may reflect the immediate therapeutic advantages of face-to-face supervision. The short-term pain-relieving effect of IPR may partly arise from its ability to reduce patients’ uncertainty and concerns about safety during the early phase of rehabilitation. In IPR treatment settings, therapists can provide immediate reassurance, clarify pain-related concerns, correct maladaptive movement patterns in real time, and build a stronger therapeutic alliance [49]. Therefore, the early benefits of IPR may reflect not only the exercise intervention itself, but also the added value of direct supervision, reassurance, and psychologically supportive care.

TLRH-AI performs in reducing pain intensity and improving health-related quality of life. However, this finding should be considered hypothesis-generating rather than conclusive, and may also reflect sparse-data effects or a winner’s curse. This advantage may be related to the ability of AI-enhanced systems to provide personalized feedback, real-time monitoring, and adaptive exercise progression. From a digital health perspective, these features are particularly relevant because they address some of the major limitations of conventional telerehabilitation, such as insufficient interactivity, delayed feedback, and limited monitoring of exercise quality. In this context, AI may enhance both the precision of exercise execution and patient adherence, thereby contributing to superior short-term symptom control. This interpretation is consistent with the increasing emphasis on personalization in digital rehabilitation, where AI-driven platforms are expected to improve intervention responsiveness through motion recognition, performance tracking, and tailored progression adjustment [49,50].

The findings for disability require cautious interpretation because they varied according to both the follow-up time point and the instrument used. On the ODI, IPR showed the greatest benefit at 4 and 12 weeks, whereas telerehabilitation ranked highest at 8 weeks, suggesting that the relative functional effects of different rehabilitation modalities may change over time. On the RMDQ, however, IPR showed the most consistent advantage. This pattern may reflect not only genuine differences in treatment effects, but also differences in what the 2 instruments capture, as the ODI covers a broader set of domains, including pain intensity, physical functioning, sleep, and social functioning [51], whereas the RMDQ was designed to capture the impact of back pain on everyday functioning and physical activities [52]. Accordingly, although the overall direction of evidence suggests that IPR may have an advantage for disability improvement, the inconsistency between ODI and RMDQ means that this conclusion should not be overstated. Rather than supporting a single, unequivocal treatment hierarchy for disability, these results suggest a more nuanced interpretation in which the comparative benefit may depend on both the stage of recovery and the outcome measure selected. This cautious interpretation is also supported by prior methodological work showing that the ODI and RMDQ cannot be used interchangeably and may not measure exactly the same construct [53]. Prior studies have likewise suggested that although remote supervision can alleviate pain and improve function, IRP may be more effective in restoring physical function and reducing kinesiophobia in patients with chronic low back pain [10,28,54]. Therefore, while digital rehabilitation may enhance accessibility and convenience, in-person care continues to play an essential role in addressing the behavioral and psychological dimensions of recovery. Interestingly, when disability was assessed using the RMDQ, available studies of TLRH-AI suggested possible benefits for disability improvement. The apparent superiority of TLRH-AI over IPR on RMDQ may partly reflect baseline differences, with TLRH-AI trials enrolling participants with milder disability (mean RMDQ ~9.2) compared to IPR trials (mean ~11.8 for IPR). This differential severity at baseline may explain why different instruments favor different interventions. These findings may indicate that TLRH-AI has the potential to support functional gains related to everyday movement performance, possibly through immediate, individualized guidance delivered in the home setting. Such support may help patients integrate therapeutic exercise more effectively into daily routines and maintain higher-quality practice outside clinical environments. However, because the evidence was limited and of low certainty, these findings should be regarded as exploratory only and not as proof of superior effectiveness.

Another notable finding is that the telerehabilitation group maintained favorable effects at 12 weeks after intervention, suggesting that structured remote exercise programs may have relatively good stability over time. Even without AI support, well-designed home-based rehabilitation protocols may help patients sustain exercise habits and reinforce core muscle function, thereby contributing to ongoing pain relief and functional improvement [55]. This observation has practical implications, as it suggests that standard telerehabilitation may still represent a valuable option when AI-supported systems are unavailable or when health care resources are limited.

The rehabilitation modalities may influence different dimensions of recovery rather than exerting a uniform effect across all outcomes. The apparent advantage of IPR for kinesiophobia may suggest that fear of movement is particularly sensitive to interventions that rebuild movement confidence through repeated therapist-guided exposure and behavioral recalibration [56,57]. In this sense, the benefit of IPR may lie not only in symptom reduction, but also in helping patients reinterpret movement as safe and manageable. By contrast, the clearer benefit of telerehabilitation for the physical component of health-related quality of life may indicate that remote rehabilitation is especially well suited to supporting physical self-management in daily life. Home-based telerehabilitation programs can provide structured exercise plans, flexible scheduling, and ongoing remote guidance, which may facilitate the transfer of exercise behaviors into routine activities and reinforce patients’ perceptions of physical capability outside the clinic [11,58]. This distinction may help explain why IPR appeared more favorable for kinesiophobia, whereas telerehabilitation showed a clearer signal for physical health status. At the same time, the nonsignificant trend toward improvement in the mental component with IPR suggests that face-to-face rehabilitation may still have psychological value, as therapeutic alliance and patients’ experiences of interactional support are regarded as meaningful aspects of low back pain care and have been linked to better outcomes in musculoskeletal rehabilitation [59,60]. Overall, these findings may reflect not a simple hierarchy of treatment superiority, but a domain-specific pattern in which different rehabilitation strategies are more responsive to different aspects of recovery.

Taken together, these findings suggest that telerehabilitation and IPR should not be viewed as competing treatment modalities, but rather as complementary components within a flexible and individualized rehabilitation framework for CNSLBP [61]. Telerehabilitation may be particularly valuable for improving access to structured exercise and maintaining rehabilitation in patients who face barriers to clinic attendance, such as mobility limitations, geographic constraints, or limited health care resources. In contrast, IPR may offer added value when closer supervision, movement retraining, functional restoration, or greater psychological support is required, especially for patients with persistent mobility impairment or fear-avoidant behaviors [3]. Within this framework, the choice between telerehabilitation and IPR should be guided not by a rigid hierarchy, but by the patient’s clinical needs, recovery stage, and practical circumstances [62,63].

Beyond comparative effectiveness, these findings also have implications for the implementation of digital rehabilitation in real-world care. Although AI-enhanced telerehabilitation appears promising, its effectiveness should be interpreted alongside issues of accessibility, digital literacy, patient engagement, and health equity. The implementation of telerehabilitation should also address long-term sustainability, the need for ongoing technical support, and integration with existing electronic health record systems [64]. These factors may influence not only adoption and scalability, but also workflow burden and care coordination in routine clinical practice [65,66].

Future telerehabilitation research should adopt more structured and reproducible approaches to reporting ethical and equity-related dimensions and should prioritize stratified analyses to clarify how race, income, age, education, and other intersecting factors influence access to, engagement with, and outcomes of digital rehabilitation interventions. Such efforts are essential to ensure that telerehabilitation is not only clinically effective but also equitable, scalable, and sustainable across diverse patient populations.

### Limitations

This study has several limitations. First, a major limitation is substantial heterogeneity in intervention protocols (duration 4-12 weeks, varying session frequency, and diverse technologies), which may violate the transitivity assumption fundamental to NMA. This means observed effect estimates may reflect specific protocol characteristics as much as intervention modality, limiting clinical exchangeability across studies. Ideally, sensitivity analyses would explore this issue, but the sparse network structure made such analyses infeasible without fragmenting evidence into unstable estimates. Therefore, findings should be interpreted as indicative trends rather than definitive comparative effectiveness rankings.

Second, assessing publication bias in NMA is challenging, particularly due to sparse evidence and stratification by time points. As a result, we did not conduct specific bias analyses for NMA, such as funnel plot correction, and the results of traditional funnel plots and the Egger test should be interpreted with caution. While funnel plot analysis can provide preliminary indications of bias, its statistical power is limited in networks with a small number of studies, and potential bias cannot be completely ruled out.

Furthermore, the SUCRA rankings reflect the relative trends in the effectiveness of different rehabilitation strategies, rather than deterministic rankings of effectiveness. Since there is currently no widely accepted method for constructing CIs for SUCRA values, they may be misleading in some cases, particularly when the evidence base is weak. As such, we present SUCRA as descriptive supplementary information, rather than a conclusive measure, and have emphasized its interpretative limitations in the discussion.

There are also issues regarding the choice of measurement tools. The ODI scale focuses more on physical function limitations, while the RMDQ scale is more sensitive to limitations in daily activities. The discrepancies in the results of these 2 scales suggest that they reflect outcomes in different ways. Regarding potential influencing factors, such as age, pain duration, and baseline severity, we acknowledge their clinical significance. However, due to our inability to obtain individual participant data, we were unable to conduct reliable formal subgroup analyses or NMA based on these factors.

Regarding missing data, we used complete outcome data from the original trial reports for analysis. Since only aggregated data were available and we could not verify the assumptions necessary for effective imputation, multiple imputation methods were not applicable. To ensure the reliability of the analysis, we assessed the convergence of the MCMC chains through visual inspection of the prespecified number of iterations and sampling behavior. This method aligns with standard practices for Bayesian NMA using *gemtc* and JAGS. However, detailed convergence diagnostic metrics, such as R-hat values, were not formally extracted, and this methodological limitation should be considered.

As for cost-effectiveness analysis, although this study included various rehabilitation interventions, most studies did not systematically report cost or cost-effectiveness data. The existing descriptive information is insufficient to support formal economic evaluations. We call for future research to prioritize conducting cost-effectiveness analysis alongside clinical trials, using a mixed-effects implementation research design to more accurately assess the economic benefits of interventions.

### Conclusion

This review adds to the field by, for the first time, using Bayesian NMA to compare telerehabilitation, TLRH-AI, IPR, and UC for CNSLBP. Telerehabilitation may be particularly advantageous for patients with stable symptoms who require sustained exercise guidance, flexible scheduling, improved accessibility, and long-term self-management support. Its potential strengths lie in extending rehabilitation beyond hospital-based services, reducing travel and resource barriers, and providing scalable, low-cost intervention delivery, especially when access to in-person services is limited. In contrast, IPR may be more suitable for patients with greater functional impairment, poor movement control, higher kinesiophobia, lower adherence, or a need for direct physical assessment and real-time correction. Its advantages are mainly reflected in therapist-supervised functional recovery, individualized movement adjustment, safety monitoring, and psychological reassurance.

Therefore, telerehabilitation and IPR should not be viewed simply as competing approaches, but rather as complementary components of individualized rehabilitation pathways. Telerehabilitation may serve as a scalable strategy for longer-term pain relief, physical function maintenance, and continuous care, whereas IPR may remain essential for patients requiring intensive supervision, functional correction, or psychological support. TLRH-AI remains exploratory and should not guide routine clinical decision-making until supported by adequately powered trials. Future research should determine whether AI-assisted feedback, motion recognition, and adaptive exercise prescription can improve adherence, safety, equity, workflow integration, and long-term outcomes in real-world CNSLBP rehabilitation.

## Data Availability

The datasets generated or analyzed during this study are available from the corresponding author on reasonable request.

## Appendix

Multimedia Appendix 1 Supplementary tables and figures.

Multimedia Appendix 2 PRISMA checklist.

## Abbreviations

AI: artificial intelligence
CNSLBP: chronic nonspecific low back pain
CrI: credible interval
GRADE: Grading of Recommendations Assessment, Development, and Evaluation
IPR: in-person rehabilitation
MCMC: Markov Chain Monte Carlo
MCS: mental component summary
MD: mean difference
MeSH: Medical Subject Headings
NMA: network meta-analysis
NRS: Numerical Rating Scale
ODI: Oswestry Disability Index
PCS: physical component summary
PI: prediction interval
PRISMA: Preferred Reporting Items for Systematic Reviews and Meta-Analyses
PRISMA-NMA: Preferred Reporting Items for Systematic Reviews and Meta-Analyses extension for Network Meta-Analyses
PRISMA-S: Preferred Reporting Items for Systematic Reviews and Meta-Analyses – Search extension
RCT: randomized controlled trial
RMDQ: Roland-Morris Disability Questionnaire
RoB: risk of bias
SF-12: 12-item Short-Form Health Survey
SMD: standardized mean difference
SUCRA: surface under the cumulative ranking curve
TLRH-AI: telerehabilitation combined with artificial intelligence
TSK: Tampa Scale for Kinesiophobia
UC: usual care
VAS: Visual Analogue Scale
