# Supplementary material for: Comparative Effectiveness of AI-Assisted Telerehabilitation, Telerehabilitation, In-Person Care, and Usual Care for Chronic Nonspecific Low Back Pain: Bayesian Network Meta-Analysis
Source: J Med Internet Res. 2026 Jul 3;28:e85410. doi: 10.2196/85410 (PMC13379692; doi:10.2196/85410)

**Supplementary Material**

**Article Title:Comparative Effectiveness of AI-Assisted Telerehabilitation, Telerehabilitation, In-Person Care and Usual Care for Chronic Nonspecific Low Back Pain: A Bayesian Network Meta-analysis**

**Contents**

**[Supplementary Table 1 PRISMA 2020 checklist 2](#_Toc30128)**

**[Supplementary Table 2 PRISMA-S checklist 5](#_Toc27380)**

**[Supplementary Table 3 PRISMA-NMA Checklist 10](#_Toc16529)**

**[Supplementary Table 4 Search strategy, Search date: April 30, 2026 14](#_Toc12270)**

**[Supplementary Table 5 Inclusion and Eligibility criteria. 21](#_Toc8170)**

**[Supplementary Table 6 Specific meaning of certainty in effect estimates. 23](#_Toc10667)**

**[SupplementaryTable7 Study Numbers and Total Sample Size in Each Network Comparison 24](#_Toc893)**

**[Supplementary Table 8](#_Toc17460) [Assessment of transitivity across intervention nodes 25](#_Toc15672)**

**[Supplementary Table 9 95% prediction intervals for key network estimates 26](#_Toc28582)**

**[Supplementary Figure 1 Risk of Bias Graph. 28](#_Toc21274)**

**[Supplementary Figure 2 SUCRA 29](#_Toc32499)**

**[Supplementary Figure 3 Funnel Plots for Assessment of Publication Bias. 33](#_Toc25980)**

**[Supplementary Figure 4 Test for inconsistency 38](#_Toc28049)**

# **Supplementary Table 1** PRISMA 2020 checklist


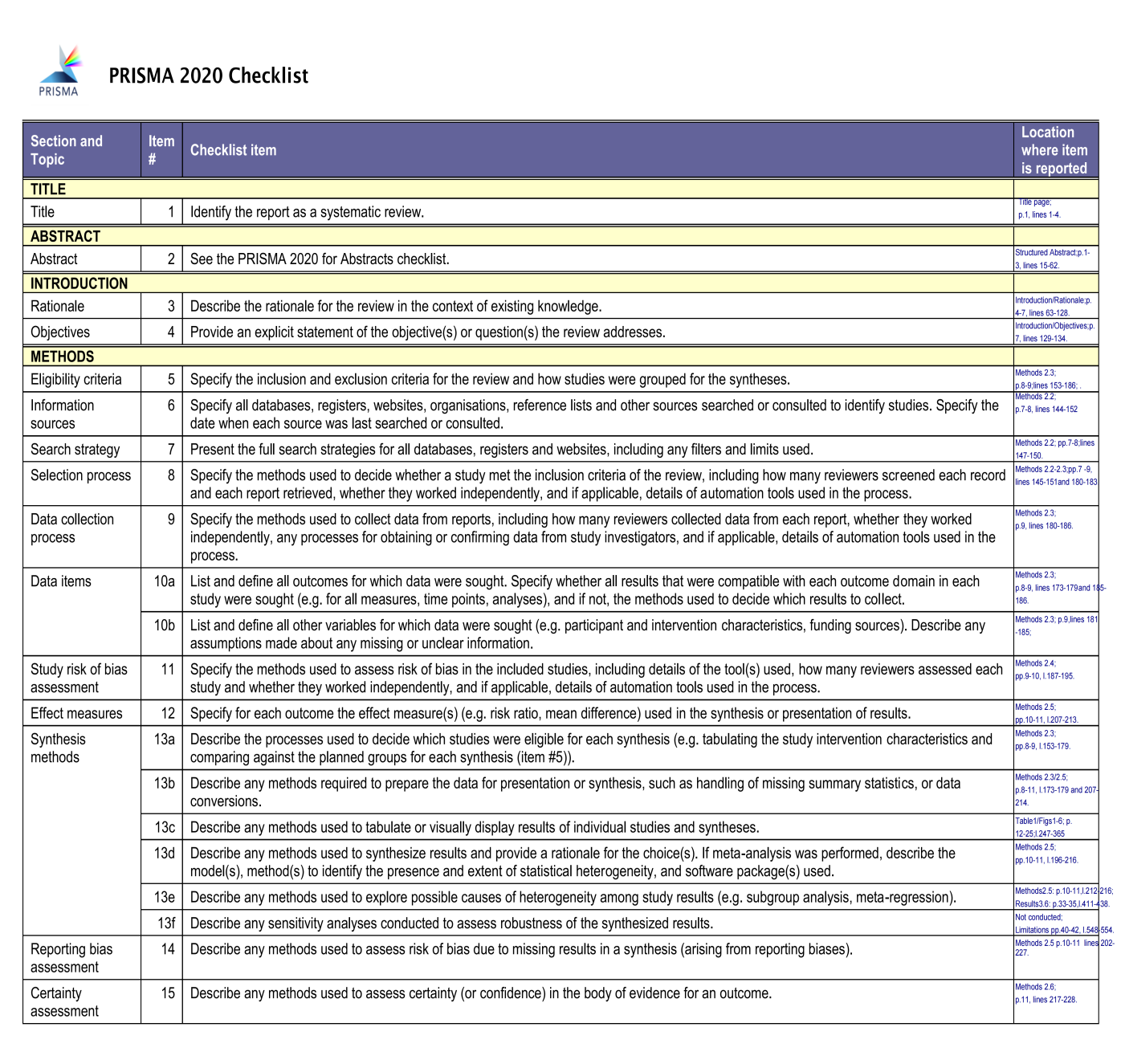


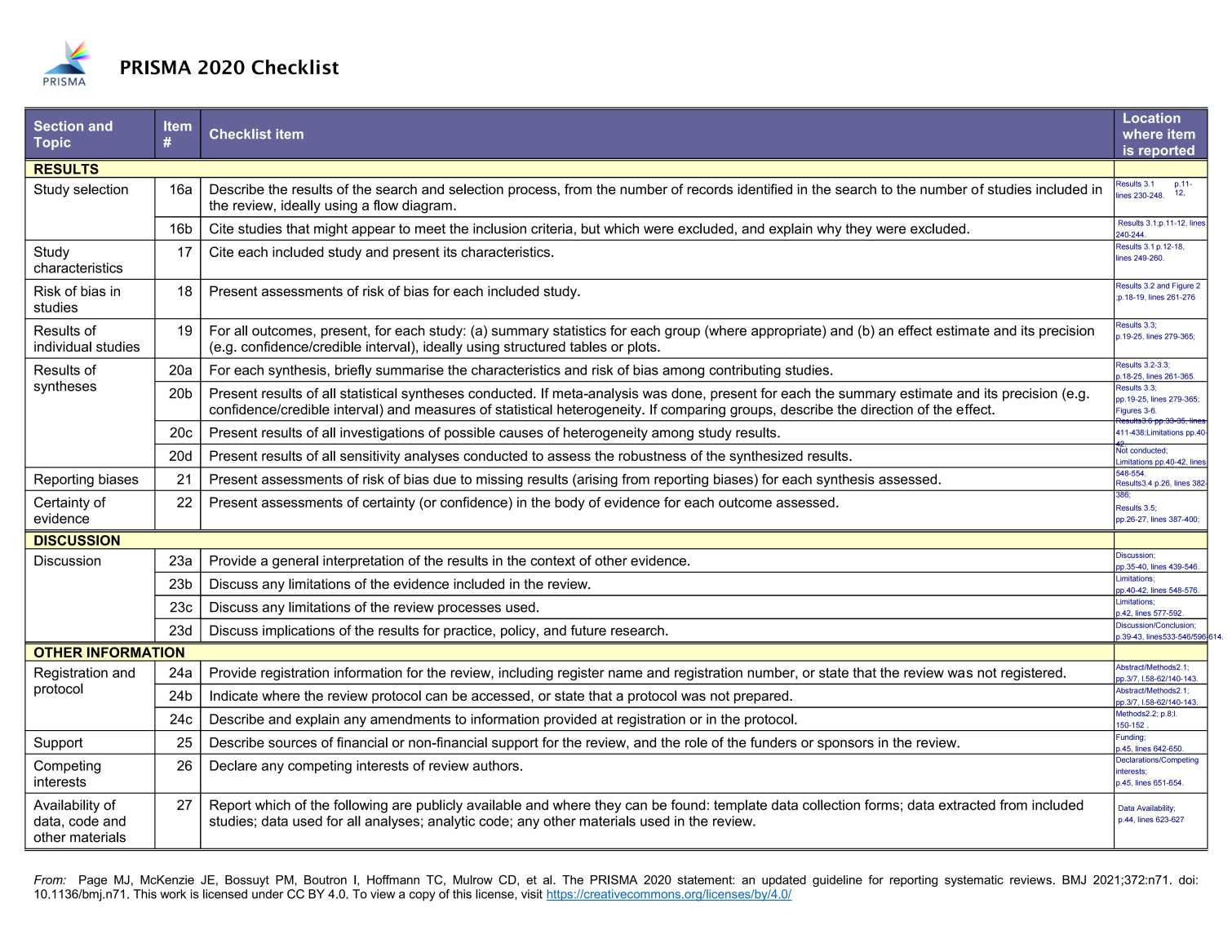


PRIMSA Abstract Checklist

| **Topic** | **No.** | **Item** | **Reported?** |
| --- | --- | --- | --- |
| **TITLE** |  |  |  |
| **Title** | 1 | Identify the report as a systematic review. | Yes |
| **BACKGROUND** |  |  |  |
| **Objectives** | 2 | Provide an explicit statement of the main objective(s) or question(s) the review addresses. | Yes |
| **METHODS** |  |  |  |
| **Eligibility criteria** | 3 | Specify the inclusion and exclusion criteria for the review. | Yes |
| **Information sources** | 4 | Specify the information sources (e.g. databases, registers) used to identify studies and the date when each was last searched. | Yes |
| **Risk of bias** | 5 | Specify the methods used to assess risk of bias in the included studies. | Yes |
| **Synthesis of results** | 6 | Specify the methods used to present and synthesize results. | Yes |
| **RESULTS** |  |  |  |
| **Included studies** | 7 | Give the total number of included studies and participants and summarise relevant characteristics of studies. | Yes |
| **Synthesis of results** | 8 | Present results for main outcomes, preferably indicating the number of included studies and participants for each. If meta-analysis was done, report the summary estimate and confidence/credible interval. If comparing groups, indicate the direction of the effect (i.e. which group is favoured). | Yes |
| **DISCUSSION** |  |  |  |
| **Limitations of evidence** | 9 | Provide a brief summary of the limitations of the evidence included in the review (e.g. study risk of bias, inconsistency and imprecision). | Yes |
| **Interpretation** | 10 | Provide a general interpretation of the results and important implications. | Yes |
| **OTHER** |  |  |  |
| **Funding** | 11 | Specify the primary source of funding for the review. | Yes |
| **Registration** | 12 | Provide the register name and registration number. | Yes |

# **Supplementary Table 2** PRISMA-S checklist


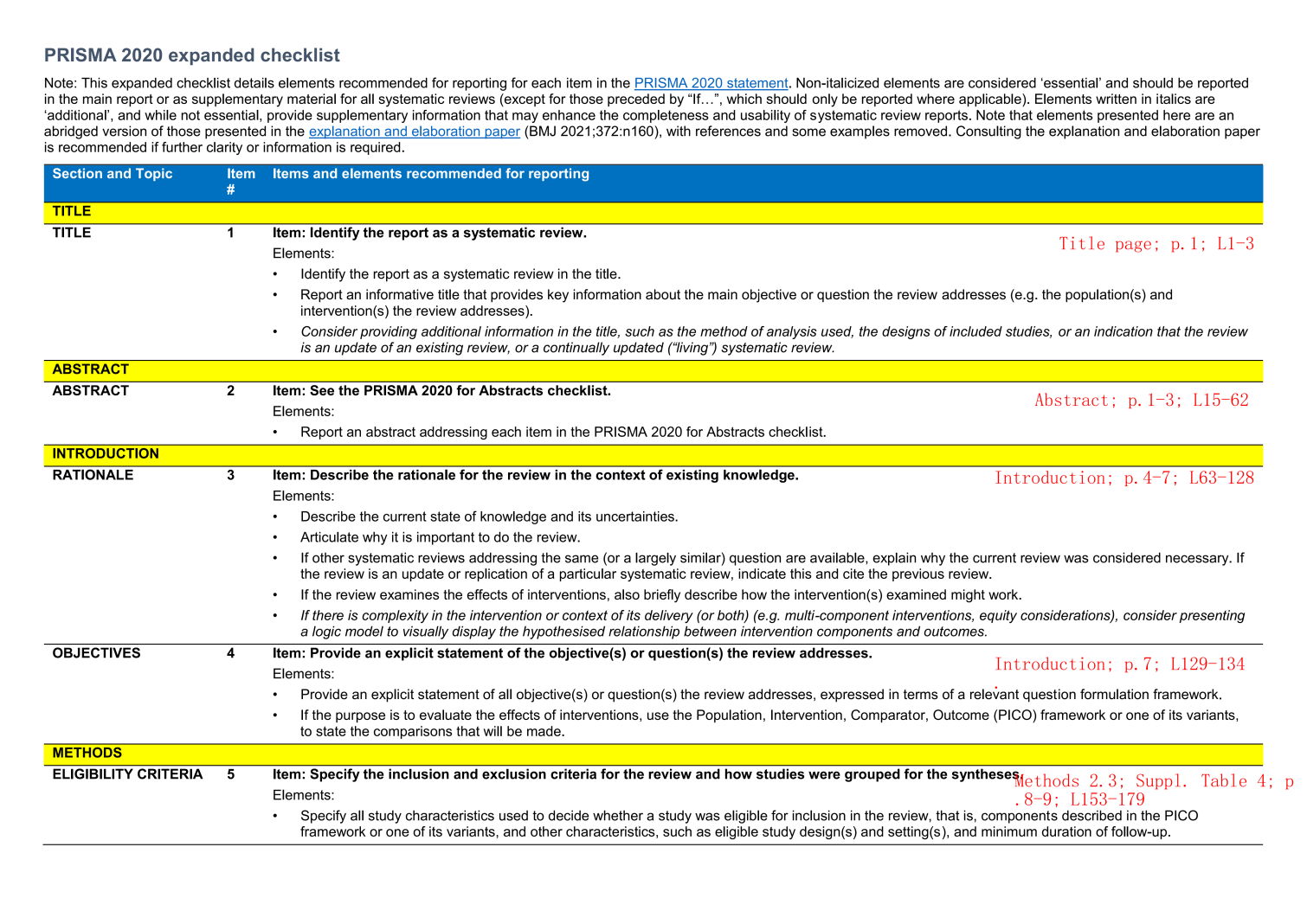


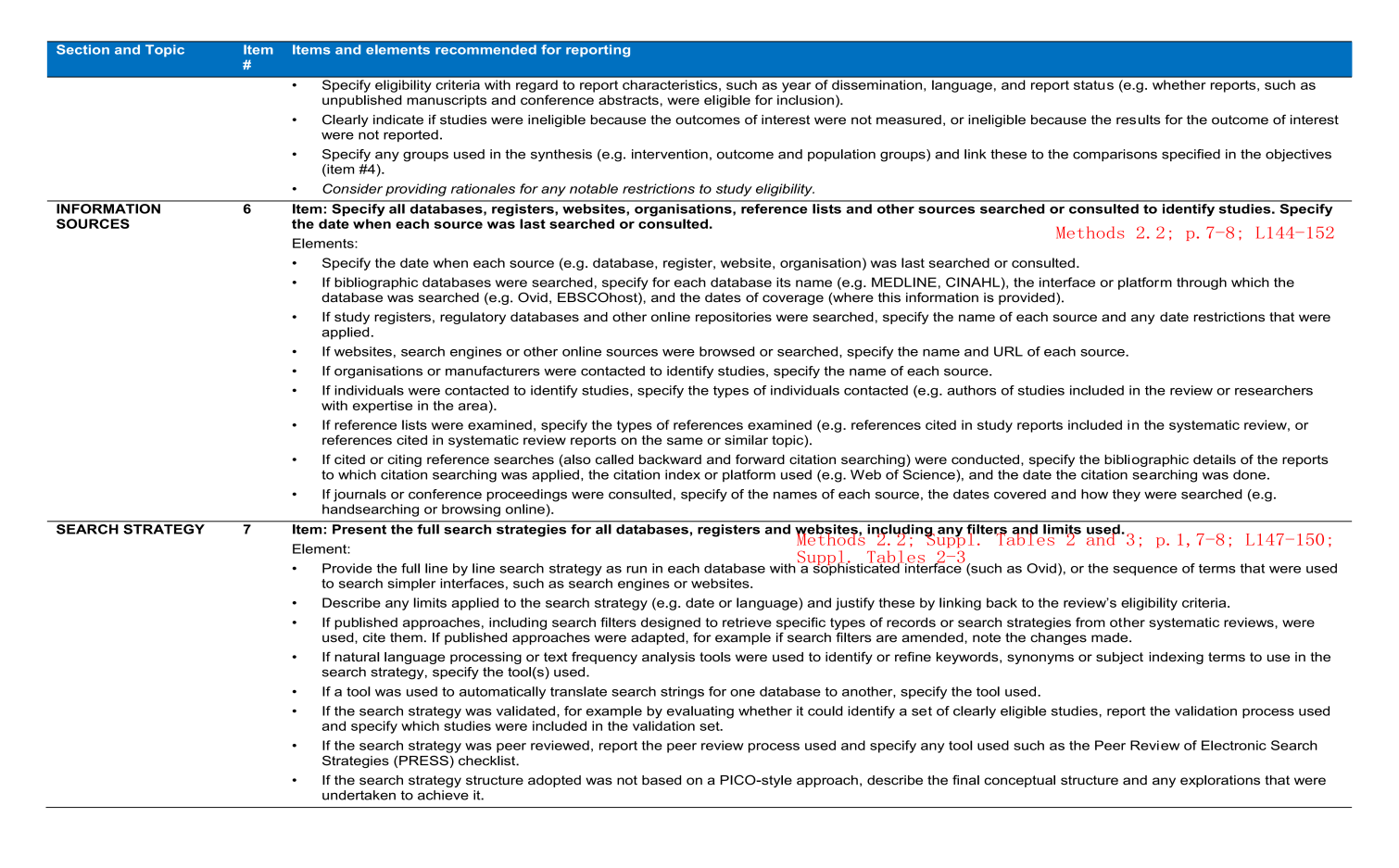


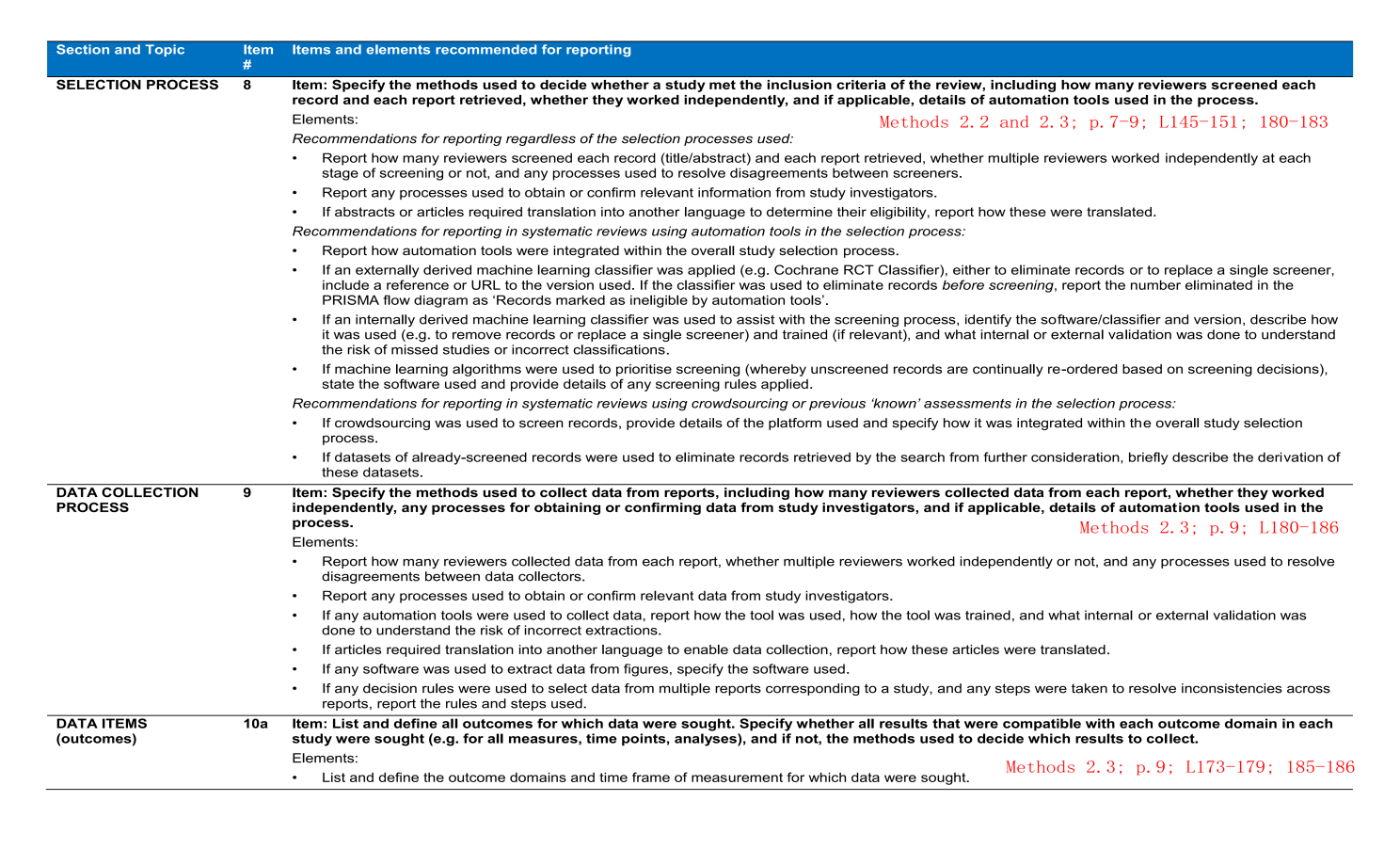


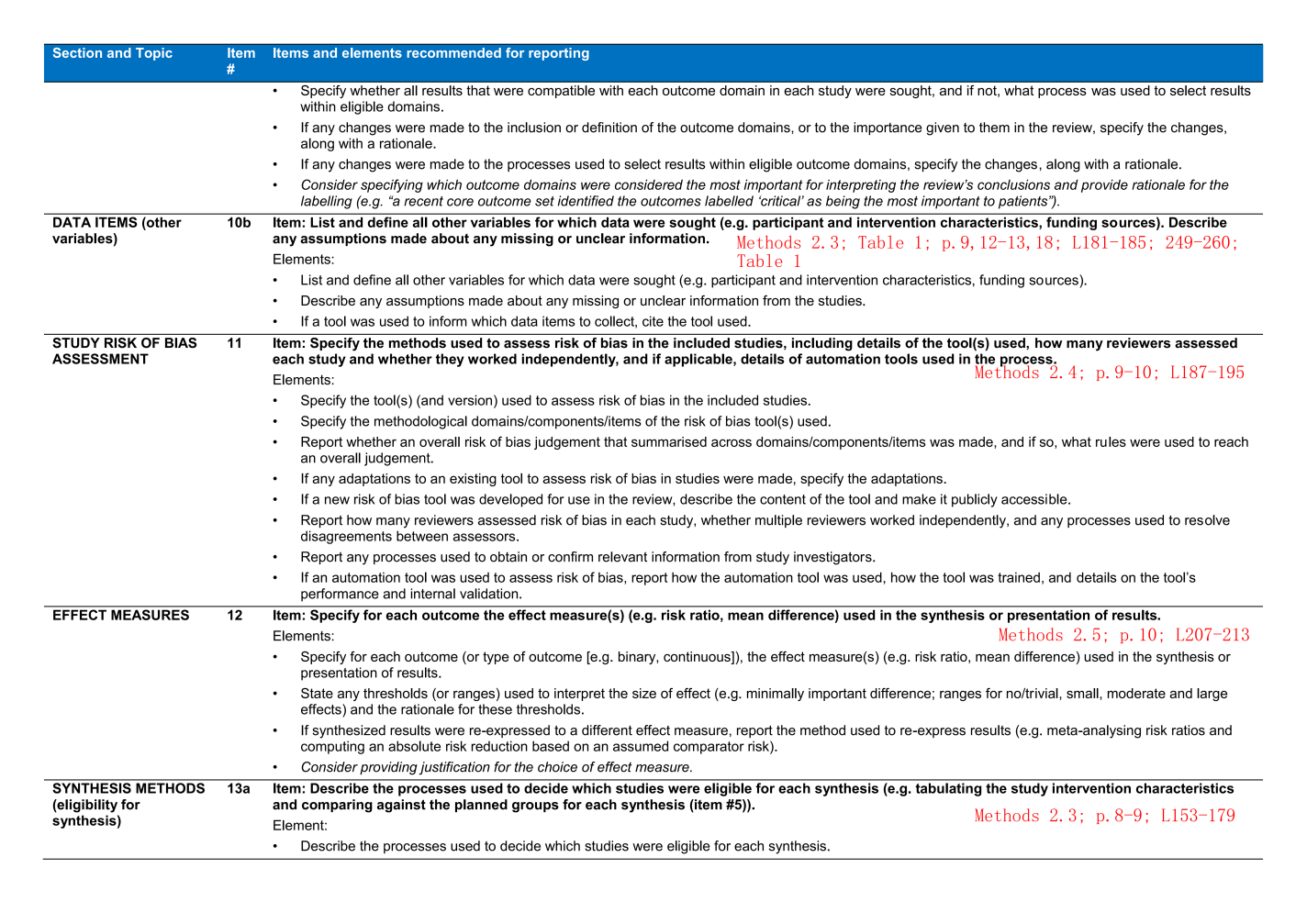


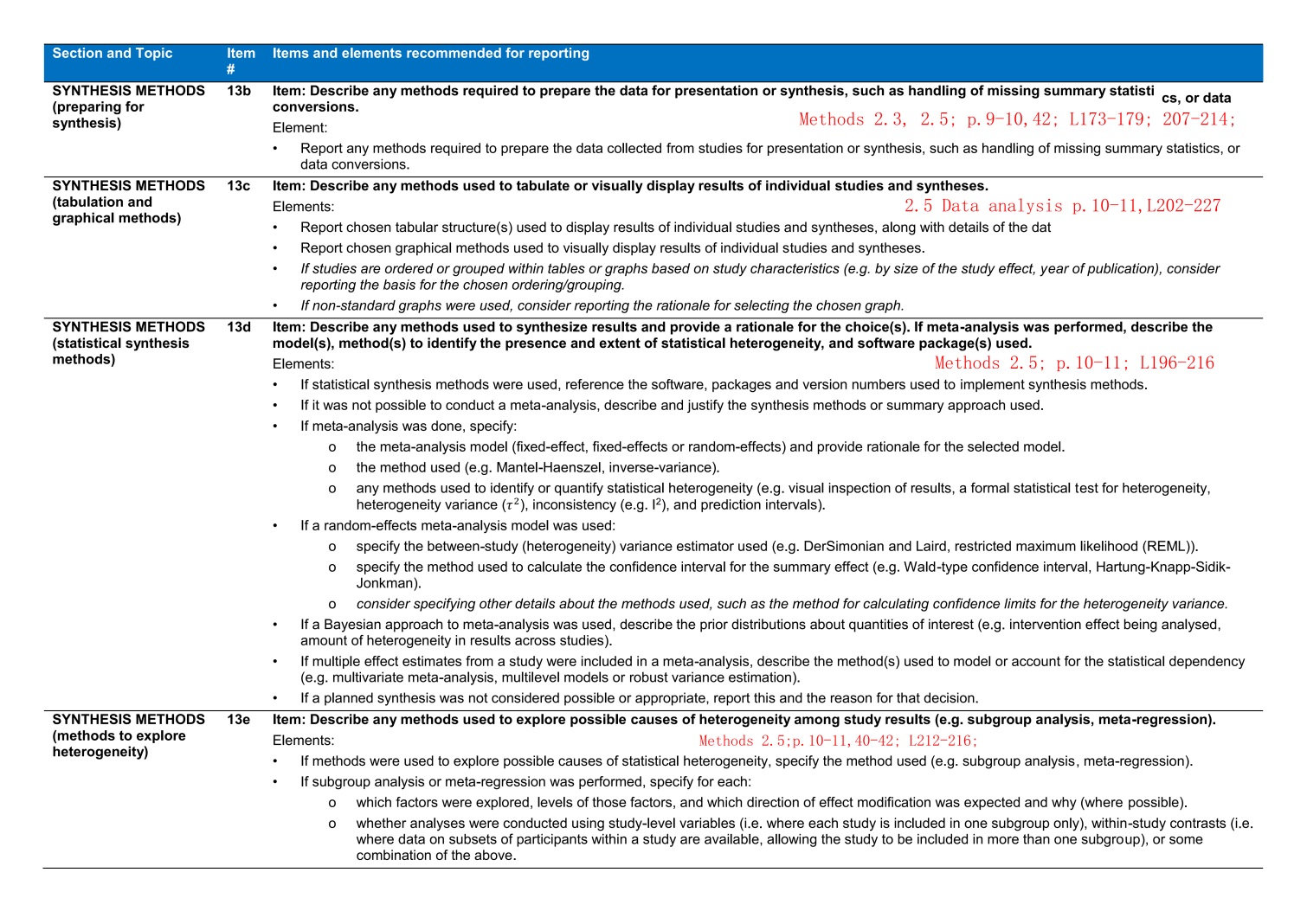


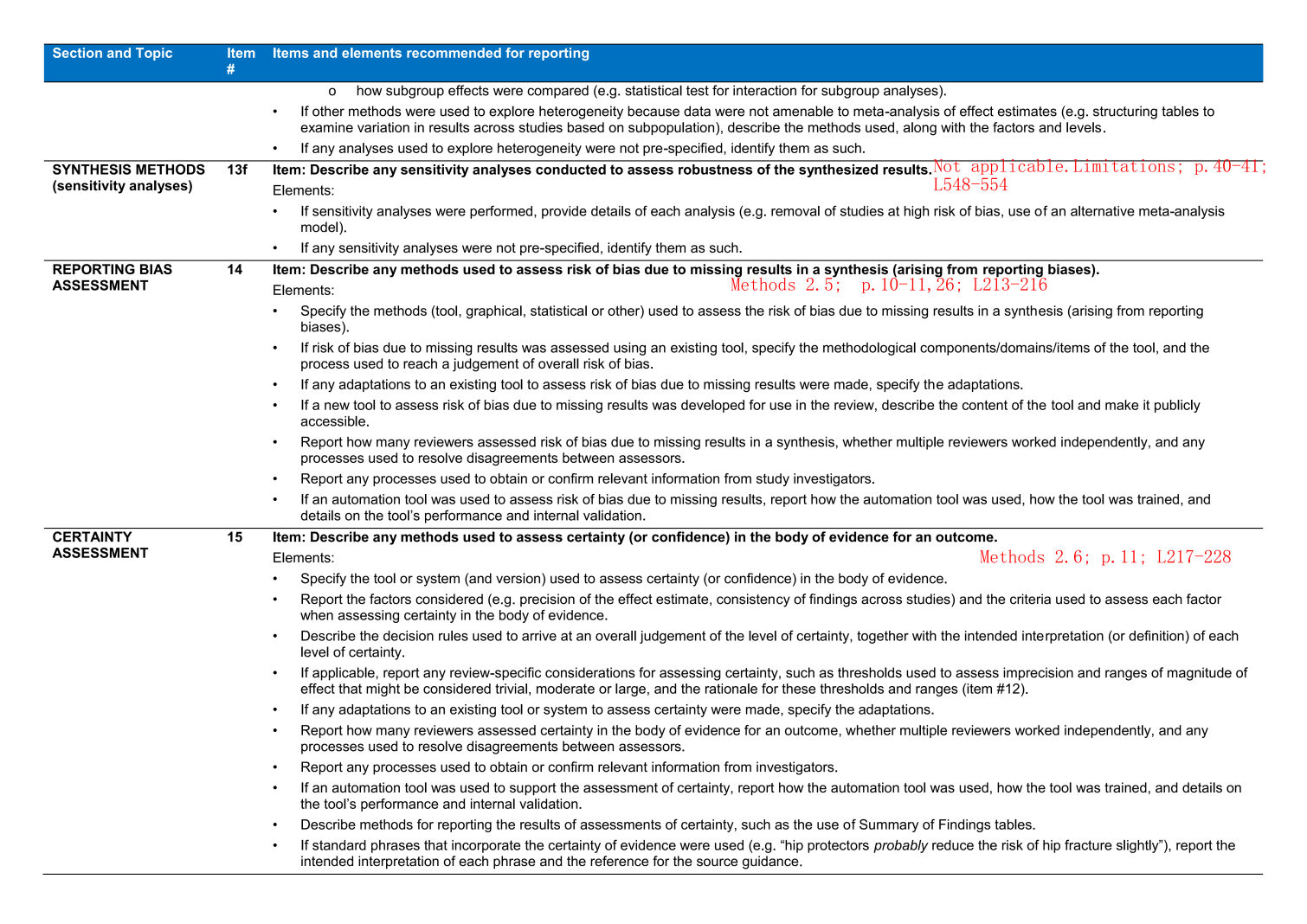

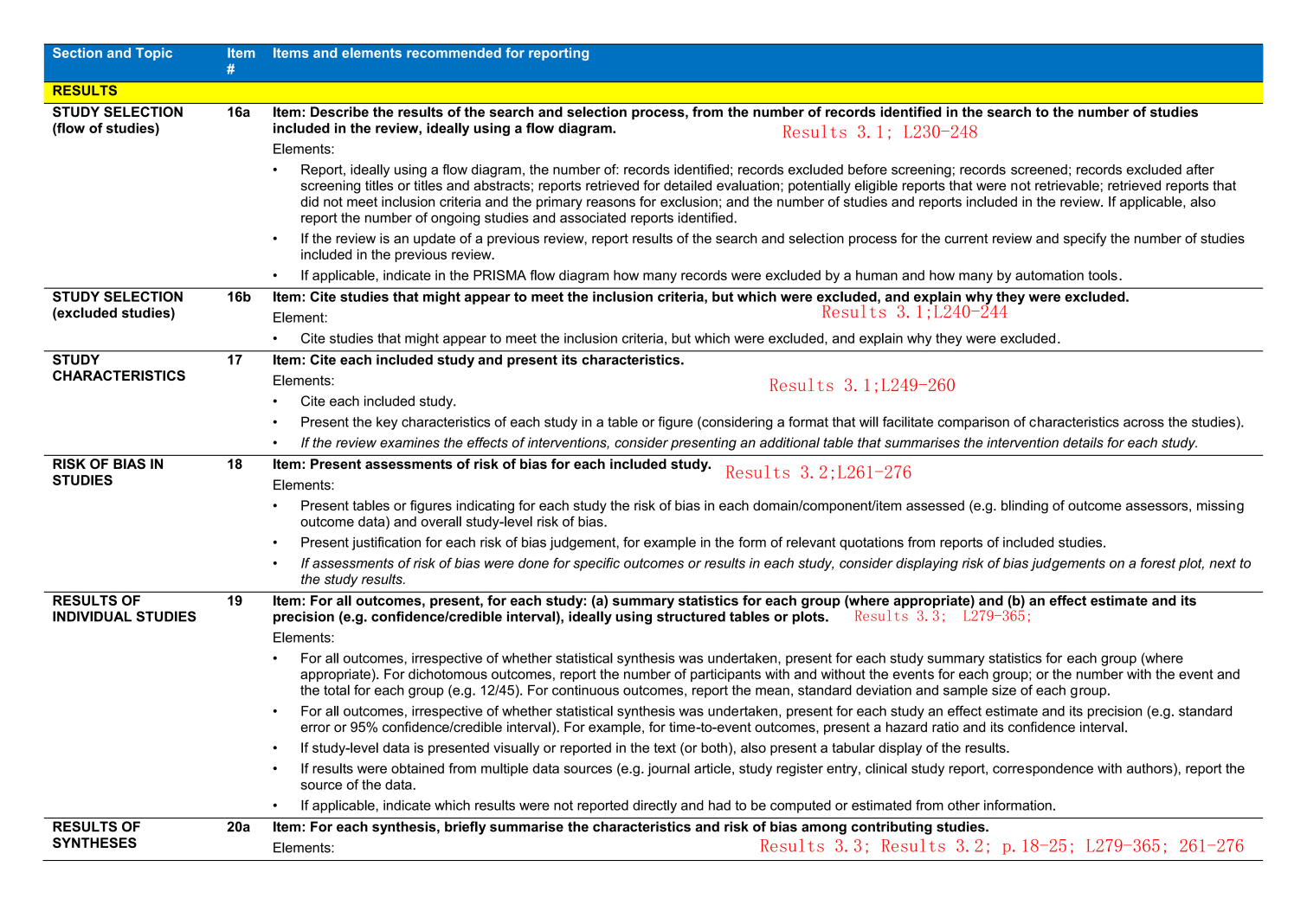


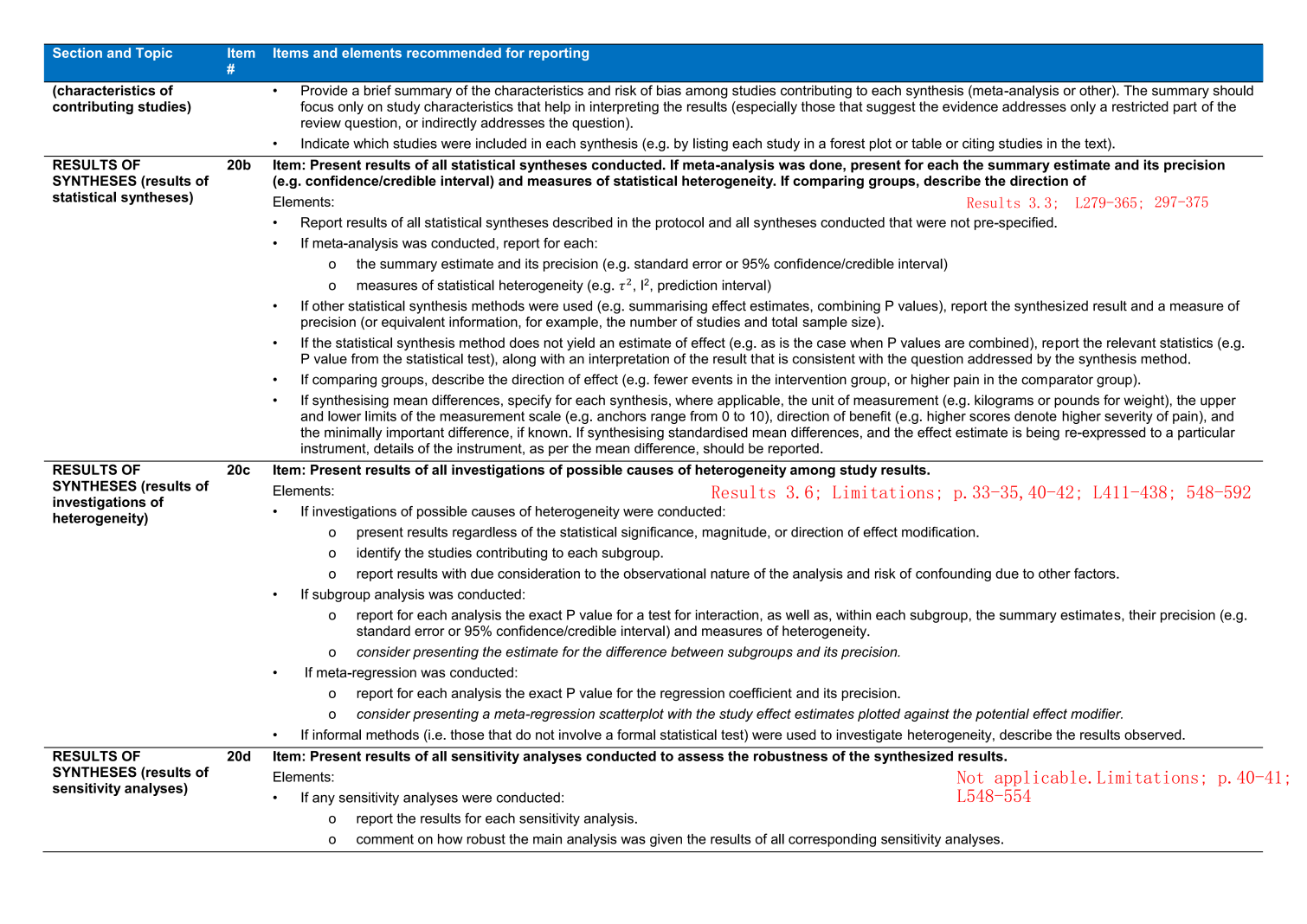


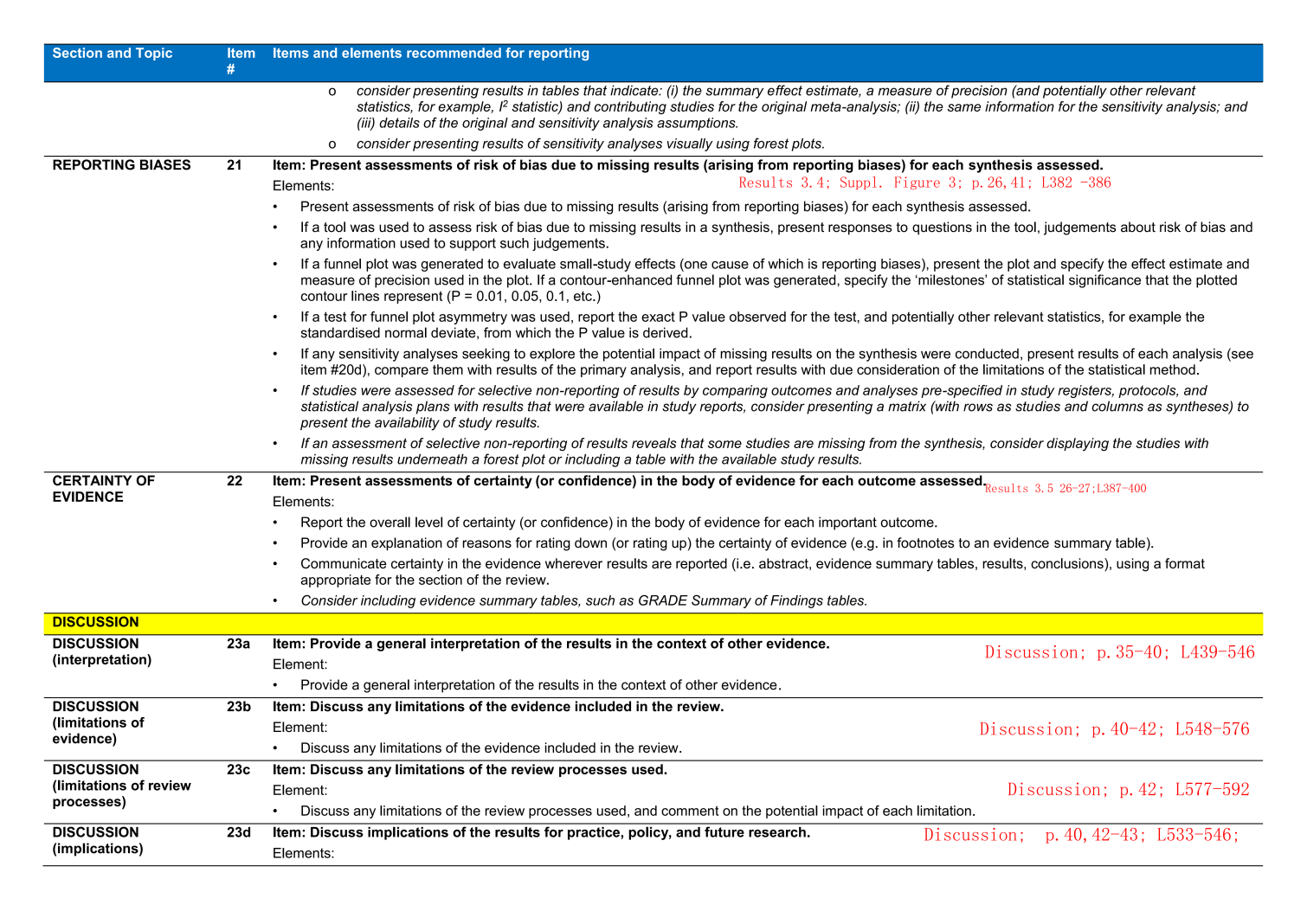

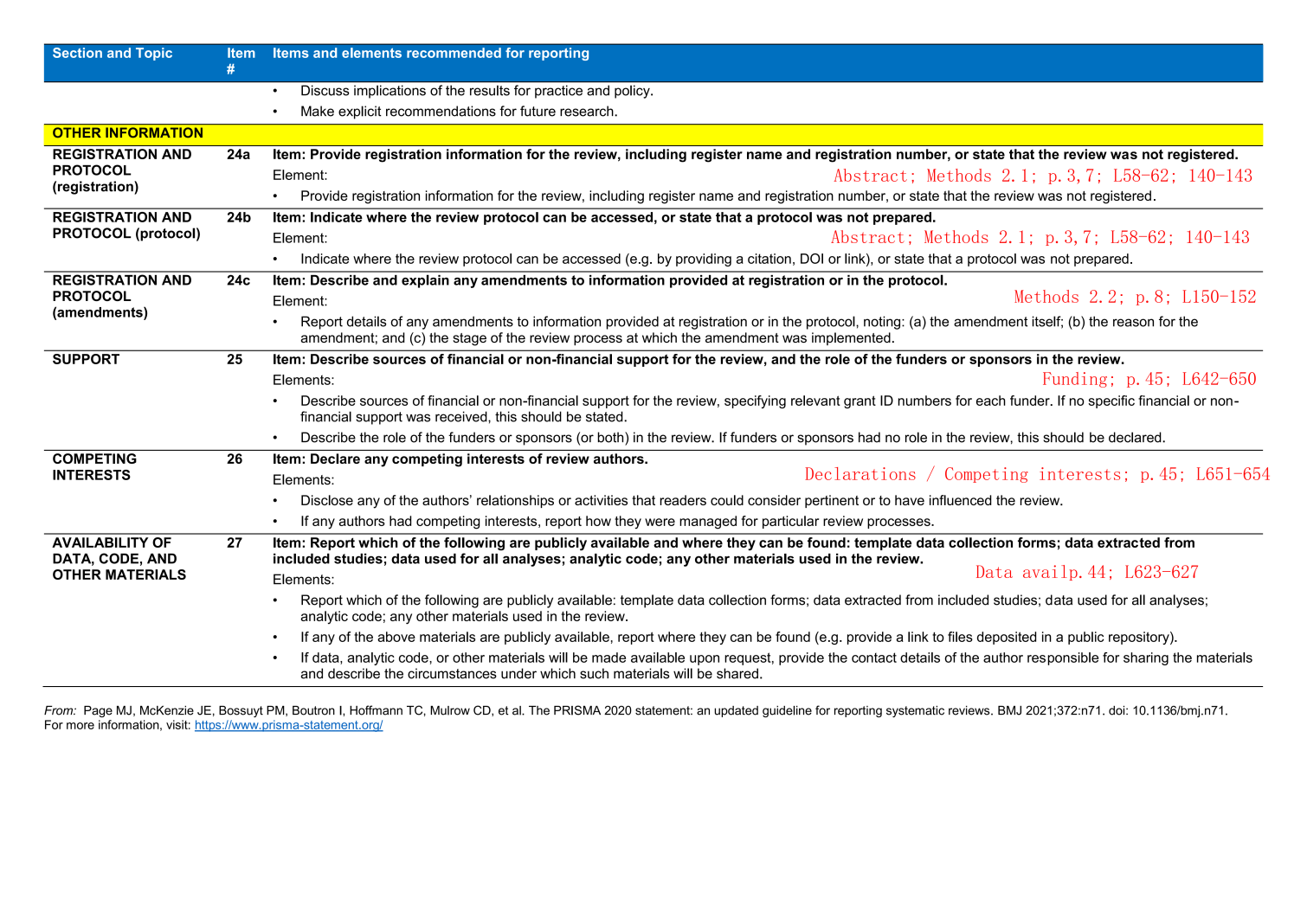


# Supplementary Table 3 PRISMA-NMA Checklist

**
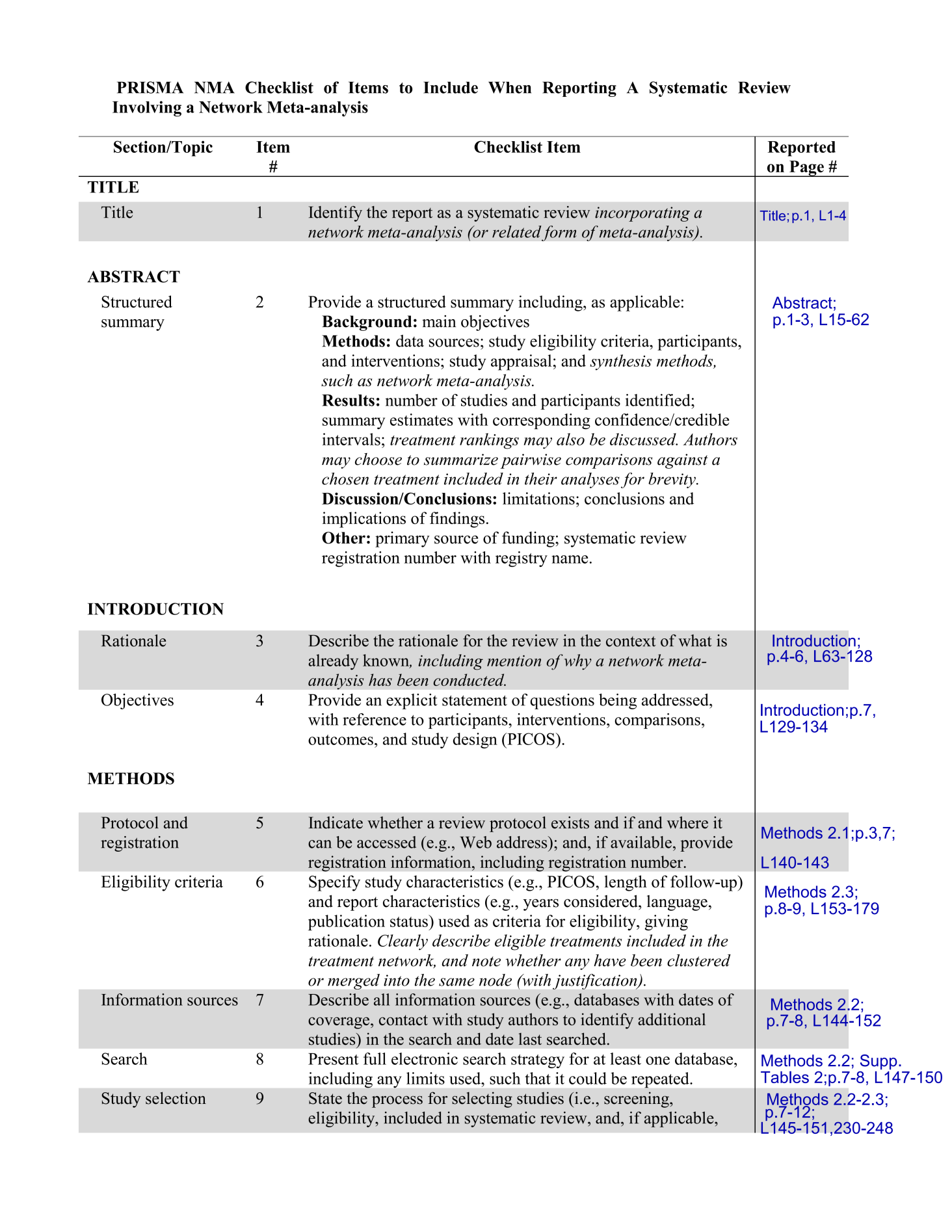
**


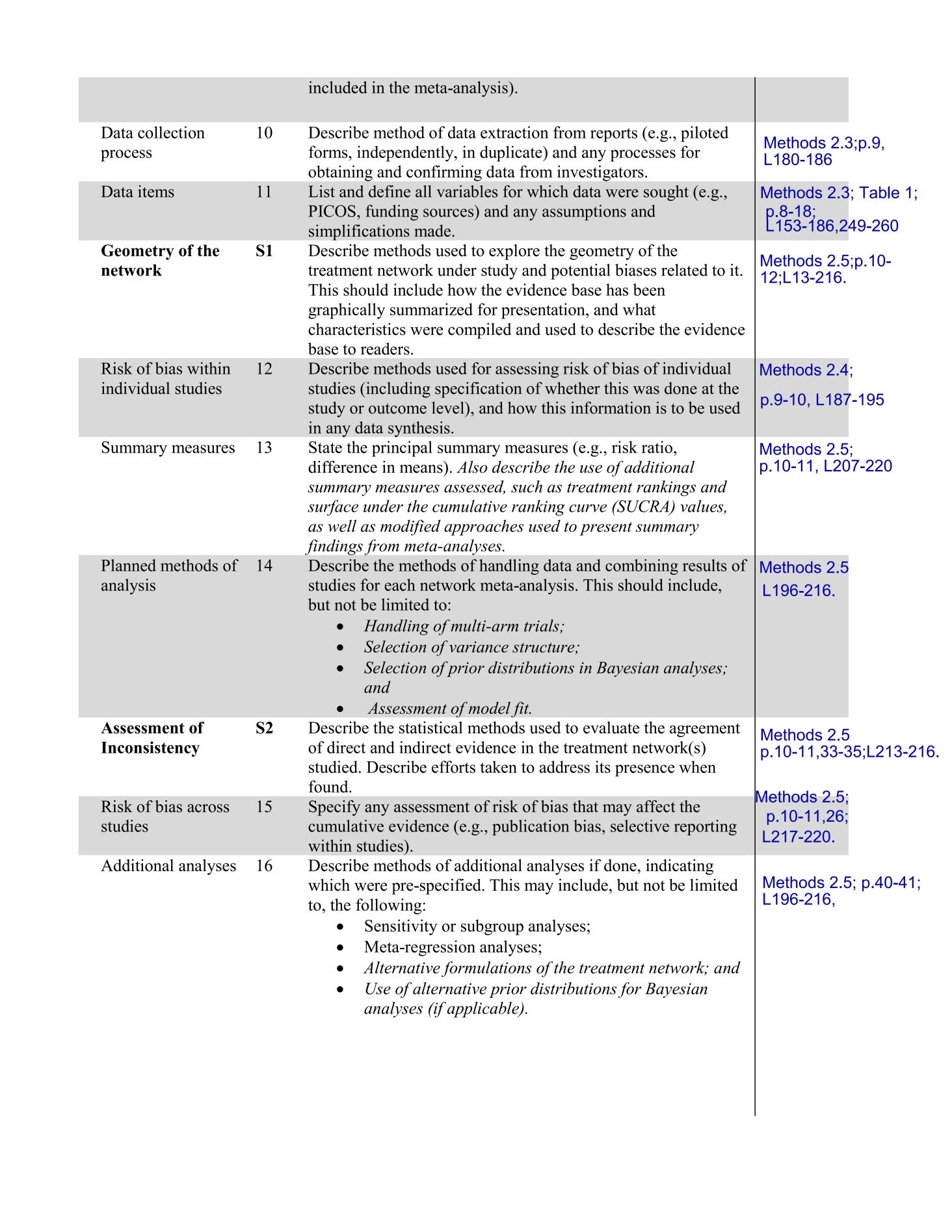

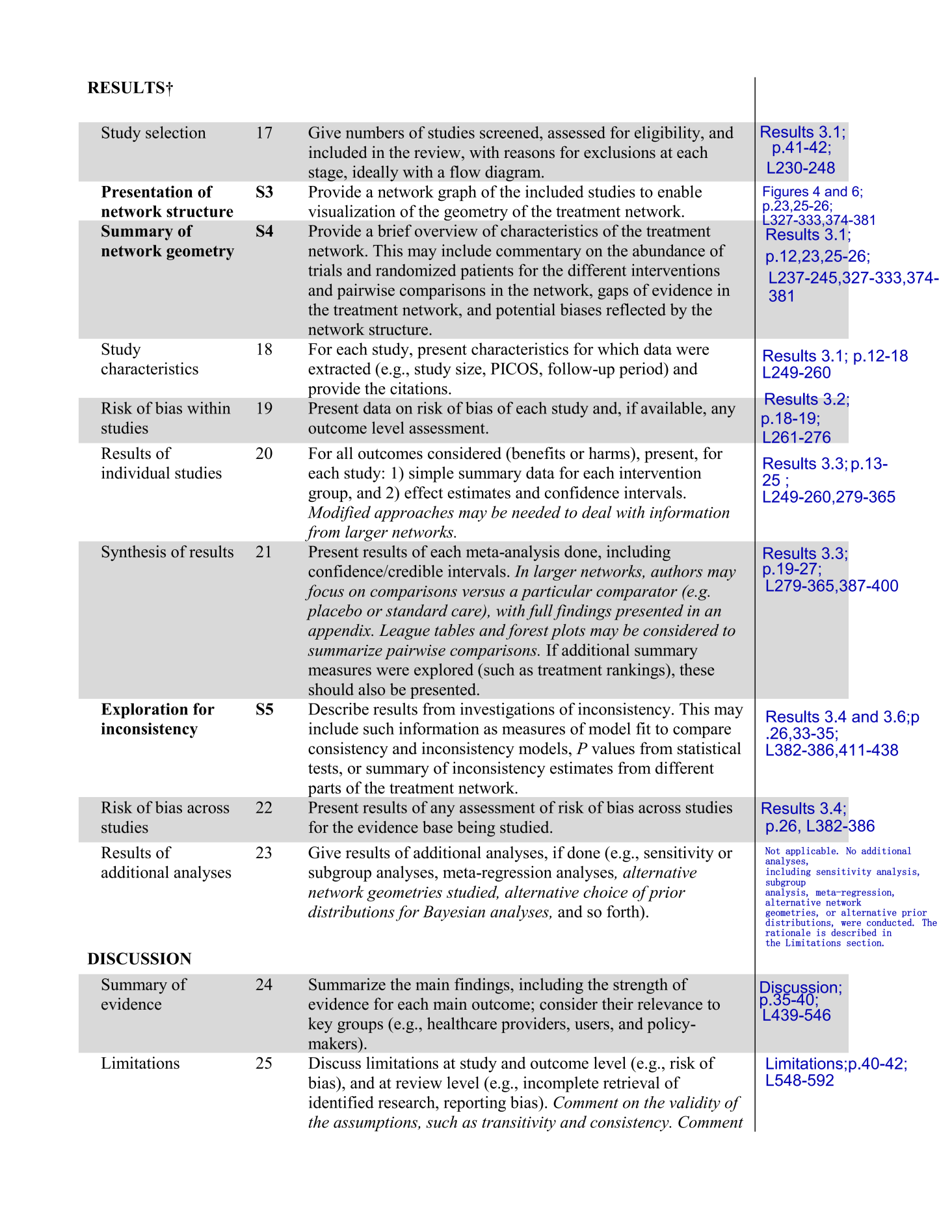

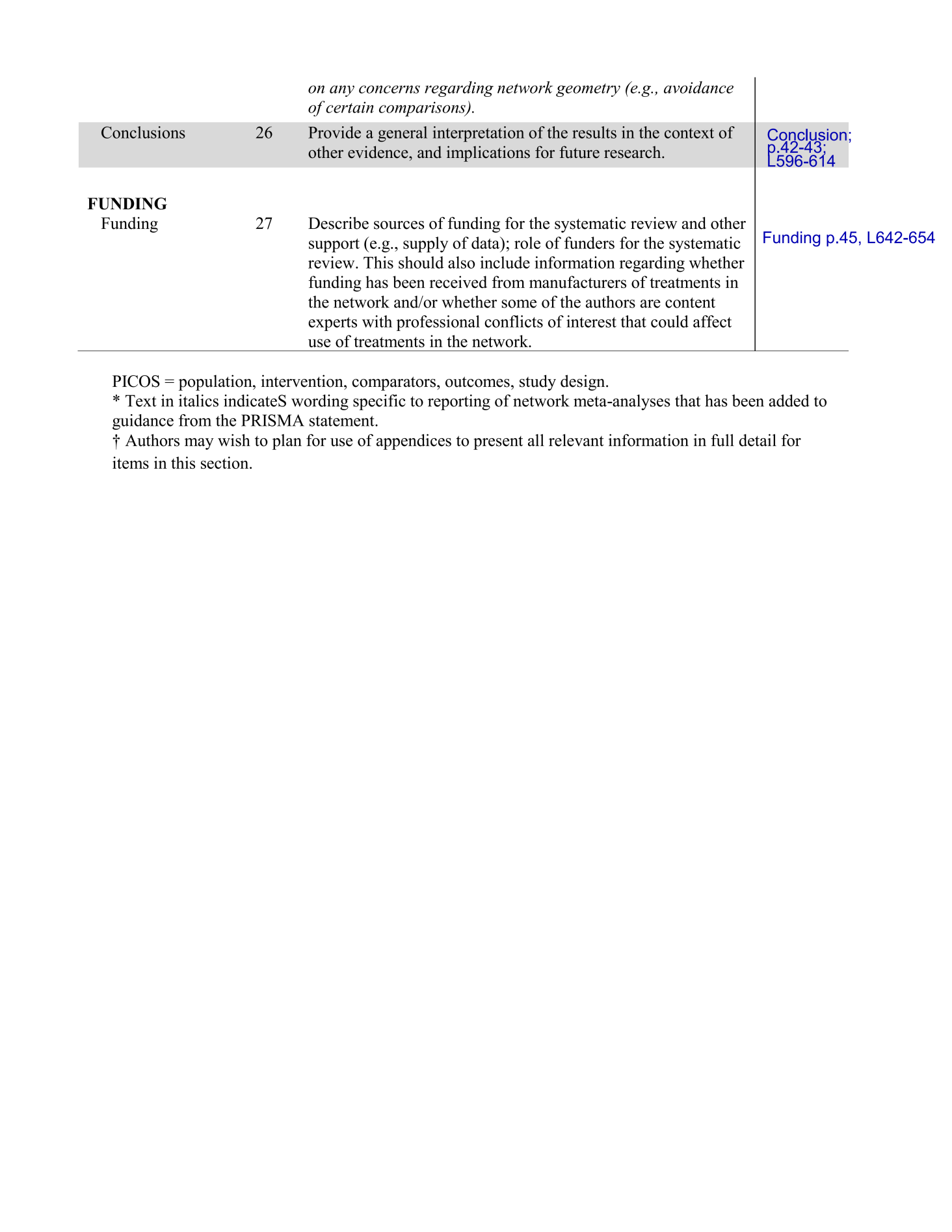


# Supplementary Table 4 Search strategy Search date: April 30, 2026

| **Database** | **Index and keyword terms Search date: April 30, 2026** | **Results** |
| --- | --- | --- |
| **PubMed** | #1 ("Telemedicine"[Mesh] OR "Telerehabilitation"[Mesh] OR "Remote Consultation"[Mesh] OR "Internet-Based Intervention"[Mesh] OR "Mobile Applications"[Mesh] OR "Cell Phone"[Mesh] OR "Smartphone"[Mesh] OR "Videoconferencing"[Mesh] OR "Wearable Electronic Devices"[Mesh] OR "Artificial Intelligence"[Mesh] OR "Machine Learning"[Mesh] OR telemedicine[tiab] OR telemedic*[tiab] OR telerehab*[tiab] OR tele-rehab*[tiab] OR telehealth[tiab] OR tele-health[tiab] OR ehealth[tiab] OR e-health[tiab] OR mhealth[tiab] OR m-health[tiab] OR "mobile health"[tiab] OR "digital health"[tiab] OR "digital medicine"[tiab] OR "digital intervention*"[tiab] OR "digital therap*"[tiab] OR "digital therapeutic*"[tiab] OR "virtual care"[tiab] OR "virtual rehabilitation"[tiab] OR "digital rehabilitation"[tiab] OR "remote rehabilitation"[tiab] OR "online rehabilitation"[tiab] OR "internet-based rehabilitation"[tiab] OR "internet based rehabilitation"[tiab] OR "web-based rehabilitation"[tiab] OR "web based rehabilitation"[tiab] OR "video-based rehabilitation"[tiab] OR "video based rehabilitation"[tiab] OR "internet-delivered"[tiab] OR "internet delivered"[tiab] OR "digitally delivered"[tiab] OR "online physiotherapy"[tiab] OR "online physical therapy"[tiab] OR "remote physiotherapy"[tiab] OR "remote physical therapy"[tiab] OR "virtual physiotherapy"[tiab] OR "virtual physical therapy"[tiab] OR telephysiotherapy[tiab] OR tele-physiotherapy[tiab] OR telecare[tiab] OR "remote care"[tiab] OR teleconsult*[tiab] OR videoconsult*[tiab] OR videoconferenc*[tiab] OR smartphone*[tiab] OR "smart phone*"[tiab] OR "mobile app*"[tiab] OR "mobile application*"[tiab] OR "mobile phone*"[tiab] OR "cell phone*"[tiab] OR cellphone*[tiab] OR app-based[tiab] OR "app based"[tiab] OR "web app*"[tiab] OR "internet app*"[tiab] OR "digital platform*"[tiab] OR "online platform*"[tiab] OR "web platform*"[tiab] OR "computer-based"[tiab] OR "computer based"[tiab] OR "video exercise*"[tiab] OR "exercise video*"[tiab] OR "home-based exercise*"[tiab] OR "home based exercise*"[tiab] OR exergam*[tiab] OR "Wii Fit"[tiab] OR selfBACK[tiab] OR "self back"[tiab] OR SupportBack[tiab] OR "Support Back"[tiab] OR PhysioAnalyst[tiab] OR "Dr AI"[tiab] OR "PAT-Back"[tiab] OR "PAT Back"[tiab] OR "machine learning"[tiab] OR "artificial intelligence"[tiab] OR AI-assisted[tiab] OR "AI assisted"[tiab] OR "automated feedback"[tiab] OR "motion recognition"[tiab] OR "posture recognition"[tiab] OR "movement recognition"[tiab] OR "real-time feedback"[tiab] OR "real time feedback"[tiab] OR "wearable sensor*"[tiab])  #2 ("Low Back Pain"[Mesh] OR "Back Pain"[Mesh] OR "low back pain"[tiab] OR "lower back pain"[tiab] OR "low-back pain"[tiab] OR "back pain"[tiab] OR backache*[tiab] OR lumbago[tiab] OR dorsalgia[tiab] OR "lumbar pain"[tiab] OR "lumbar spine pain"[tiab] OR "mechanical low back pain"[tiab] OR "nonspecific low back pain"[tiab] OR "non-specific low back pain"[tiab] OR "non specific low back pain"[tiab] OR "chronic low back pain"[tiab] OR "chronic nonspecific low back pain"[tiab] OR "chronic non-specific low back pain"[tiab] OR "persistent low back pain"[tiab] OR "recurrent low back pain"[tiab] OR "spinal pain"[tiab])  #3 (randomized controlled trial[pt] OR controlled clinical trial[pt] OR clinical trial[pt] OR "Randomized Controlled Trials as Topic"[Mesh] OR "Random Allocation"[Mesh] OR "Clinical Trials as Topic"[Mesh] OR randomized[tiab] OR randomised[tiab] OR randomly[tiab] OR randomization[tiab] OR randomisation[tiab] OR RCT[tiab] OR trial[tiab] OR "controlled trial"[tiab] OR "clinical trial"[tiab] OR "pilot trial"[tiab] OR "feasibility trial"[tiab] OR "pragmatic trial"[tiab])  #4 (animals[mh] NOT humans[mh])  #5 #1 AND #2 AND #3 NOT #4 | #1 669670  #2 92036  #3 2451576  #4 5451261  #5 475 |
| **Cochrane** | ID Search Hits |  |
|  | #1 MeSH descriptor: [Telemedicine] explode all trees  #2 MeSH descriptor: [Telerehabilitation] explode all trees  #3 MeSH descriptor: [Remote Consultation] explode all trees  #4 MeSH descriptor: [Mobile Applications] explode all trees  #5 MeSH descriptor: [Cell Phone] explode all trees  #6 MeSH descriptor: [Smartphone] explode all trees  #7 MeSH descriptor: [Videoconferencing] explode all trees  #8 MeSH descriptor: [Wearable Electronic Devices] explode all trees  #9 MeSH descriptor: [Artificial Intelligence] explode all trees  #10 MeSH descriptor: [Machine Learning] explode all trees  #11 (telemedicine OR telemedic* OR telerehab* OR tele-rehab* OR telehealth* OR tele-health OR eHealth OR e-health OR mHealth OR m-health OR "mobile health" OR "digital health" OR "digital medicine" OR (digital NEXT intervention*) OR (digital NEXT therap*) OR (digital NEXT therapeutic*) OR "virtual care" OR "virtual rehabilitation" OR "digital rehabilitation" OR "remote rehabilitation" OR "online rehabilitation" OR "internet-based rehabilitation" OR "internet based rehabilitation" OR "web-based rehabilitation" OR "web based rehabilitation" OR "video-based rehabilitation" OR "video based rehabilitation" OR internet-delivered OR "internet delivered" OR "digitally delivered" OR "online physiotherapy" OR "online physical therapy" OR "remote physiotherapy" OR "remote physical therapy" OR "virtual physiotherapy" OR "virtual physical therapy" OR telephysiotherapy OR tele-physiotherapy OR telecare OR "remote care" OR teleconsult* OR videoconsult* OR videoconferenc* OR smartphone* OR (smart NEXT phone*) OR (mobile NEXT app*) OR (mobile NEXT application*) OR (mobile NEXT phone*) OR (cell NEXT phone*) OR cellphone* OR app-based OR "app based" OR (web NEXT app*) OR (internet NEXT app*) OR (digital NEXT platform*) OR (online NEXT platform*) OR (web NEXT platform*) OR computer-based OR "computer based" OR (video NEXT exercise*) OR (exercise NEXT video*) OR (home-based NEXT exercise*) OR (home NEXT exercise*) OR exergam* OR "Wii Fit" OR selfBACK OR "self back" OR SupportBack OR "Support Back" OR PhysioAnalyst OR "Dr AI" OR "PAT-Back" OR "PAT Back" OR "artificial intelligence" OR "machine learning" OR AI-assisted OR "AI assisted" OR (automated NEXT feedback) OR (motion NEXT recognition) OR (posture NEXT recognition) OR (movement NEXT recognition) OR (real-time NEXT feedback) OR (real NEXT time NEXT feedback) OR (wearable NEXT sensor*)):ti,ab,kw#12 #1 OR #2 OR #3 OR #4 OR #5 OR #6 OR #7 OR #8 OR #9 OR #10 OR #11  #13 MeSH descriptor: [Low Back Pain] explode all trees  #14 MeSH descriptor: [Back Pain] explode all trees  #15 ("low back pain" OR "lower back pain" OR "low-back pain" OR "back pain" OR backache* OR lumbago OR dorsalgia OR "lumbar pain" OR "lumbar spine pain" OR "spinal pain" OR "mechanical low back pain" OR "nonspecific low back pain" OR "non-specific low back pain" OR "non specific low back pain" OR "chronic low back pain" OR "chronic nonspecific low back pain" OR "chronic non-specific low back pain" OR "persistent low back pain" OR "recurrent low back pain" OR ((low OR lower OR lumbar) NEAR/3 (back OR spine OR spinal) NEAR/3 pain)):ti,ab,kw  #16 #13 OR #14 OR #15  #17 MeSH descriptor: [Exercise Therapy] explode all trees  #18 MeSH descriptor: [Physical Therapy Modalities] explode all trees  #19 MeSH descriptor: [Rehabilitation] explode all trees  #20 (exercise* OR rehabilitat* OR physiotherap* OR physical NEXT therap* OR therapeutic NEXT exercise* OR movement NEXT therapy OR functional NEXT rehabilitation OR home NEXT exercise* OR home-based NEXT exercise* OR supervised NEXT exercise* OR guided NEXT exercise* OR exercise NEXT program* OR exercise NEXT training OR exercise NEXT prescription* OR motor NEXT control NEXT exercise* OR core NEXT stability NEXT exercise* OR core NEXT stabilization NEXT exercise* OR stabilization NEXT exercise* OR strengthening NEXT exercise* OR stretching NEXT exercise* OR aerobic NEXT exercise* OR resistance NEXT training OR walking OR Pilates OR yoga OR McKenzie):ti,ab,kw  #21 #17 OR #18 OR #19 OR #20  #22 ("randomized controlled trial" OR "randomised controlled trial" OR "controlled clinical trial" OR randomi?ed OR randomly OR randomi?ation OR RCT OR trial* OR (clinical NEXT trial*) OR (pilot NEXT trial*) OR (feasibility NEXT trial*) OR (pragmatic NEXT trial*)):ti,ab,kw  #23 MeSH descriptor: [Animals] explode all trees  #24 MeSH descriptor: [Humans] explode all trees  #25 #23 NOT #24  #26 #12 AND #16 AND #22 NOT #25  #27 #12 AND #16 AND #21 AND #22 NOT #25 | #1 69  #2 15  #3 96  #4 77  #5 126  #6 76  #7 35  #8 18  #9 35  #10 100  #11 63492  #12 63784  #13 783  #14 788  #15 25223  #16 25946  #17 767  #18 414  #19 631  #20 260436  #21 261165  #22 1660535  #23 2465  #24 3555  #25 12  #26 885  #27 674 |
| EMBASE | #1 ('telemedicine'/exp OR 'telerehabilitation'/exp OR 'telehealth'/exp OR 'ehealth'/exp OR 'mobile health'/exp OR 'mobile application'/exp OR 'smartphone'/exp OR 'videoconferencing'/exp OR 'artificial intelligence'/exp OR 'machine learning'/exp OR 'wearable device'/exp OR (telemedicine OR telemedic* OR telerehab* OR tele-rehab* OR telehealth* OR 'tele-health' OR ehealth* OR 'e-health' OR mhealth* OR 'm-health' OR 'mobile health' OR 'digital health' OR 'digital medicine' OR 'digital intervention*' OR 'digital therap*' OR 'digital therapeutic*' OR 'mobile rehabilitation' OR 'virtual care' OR 'virtual rehabilitation' OR 'digital rehabilitation' OR 'remote rehabilitation' OR 'online rehabilitation' OR 'internet-based rehabilitation' OR 'internet based rehabilitation' OR 'web-based rehabilitation' OR 'web based rehabilitation' OR 'video-based rehabilitation' OR 'video based rehabilitation' OR 'internet-delivered' OR 'internet delivered' OR 'digitally delivered' OR 'online physiotherapy' OR 'online physical therapy' OR 'remote physiotherapy' OR 'remote physical therapy' OR 'virtual physiotherapy' OR 'virtual physical therapy' OR telephysiotherapy OR tele-physiotherapy OR telecare OR 'remote care' OR teleconsult* OR videoconsult* OR videoconferenc* OR smartphone* OR 'smart phone*' OR 'mobile app*' OR 'mobile application*' OR 'mobile phone*' OR 'cell phone*' OR cellphone* OR app-based OR 'app based' OR 'web app*' OR 'internet app*' OR 'digital platform*' OR 'online platform*' OR 'web platform*' OR 'computer-based' OR 'computer based' OR 'video exercise*' OR 'exercise video*' OR 'home-based exercise*' OR 'home based exercise*' OR exergam* OR exergaming OR 'Wii Fit' OR selfBACK OR 'self back' OR SupportBack OR 'Support Back' OR PhysioAnalyst OR 'Dr AI' OR 'PAT-Back' OR 'PAT Back' OR 'artificial intelligence' OR 'machine learning' OR AI-assisted OR 'AI assisted' OR 'automated feedback' OR 'motion recognition' OR 'posture recognition' OR 'movement recognition' OR 'real-time feedback' OR 'real time feedback' OR 'wearable sensor*' OR 'wearable device*'):ti,ab,kw)  #2 ('low back pain'/exp OR ('low back pain' OR 'lower back pain' OR 'low-back pain' OR 'back pain' OR backache* OR lumbago OR dorsalgia OR 'lumbar pain' OR 'lumbar spine pain' OR 'spinal pain' OR 'mechanical low back pain' OR 'nonspecific low back pain' OR 'non-specific low back pain' OR 'non specific low back pain' OR 'chronic low back pain' OR 'chronic nonspecific low back pain' OR 'chronic non-specific low back pain' OR 'chronic non specific low back pain' OR 'persistent low back pain' OR 'recurrent low back pain' OR 'acute low back pain' OR 'radicular pain' OR sciatica OR 'lumbar radiculopathy' OR 'disc herniation' OR 'disk herniation' OR 'lumbar disc disease' OR 'spinal degeneration'):ti,ab,kw)  #3 ('exercise therapy'/exp OR 'physiotherapy'/exp OR 'physical therapy'/exp OR 'rehabilitation'/exp OR (exercis* OR rehabilitat* OR physiotherap* OR 'physical therap*' OR 'therapeutic exercis*' OR 'movement therapy' OR 'functional rehabilitation' OR 'home exercis*' OR 'home-based exercis*' OR 'home based exercis*' OR 'supervised exercis*' OR 'guided exercis*' OR 'exercise program*' OR 'exercise training' OR 'exercise prescription*' OR 'exercise therap*' OR 'motor control exercis*' OR 'core stability exercis*' OR 'core stabilization exercis*' OR 'stabilization exercis*' OR 'strengthening exercis*' OR 'stretching exercis*' OR 'aerobic exercis*' OR 'resistance training' OR walking OR Pilates OR yoga OR McKenzie OR 'physical rehabilitation' OR 'self-management' OR 'patient education'):ti,ab,kw)  #4 ('randomized controlled trial'/exp OR 'controlled clinical trial'/exp OR 'clinical trial'/exp OR 'randomization'/exp OR (random* OR randomised OR randomized OR randomly OR randomisation OR randomization OR rct OR trial* OR 'clinical trial*' OR 'controlled trial*' OR 'randomized trial*' OR 'randomised trial*' OR 'pilot trial*' OR 'feasibility trial*' OR 'pragmatic trial*' OR placebo OR 'comparative study' OR 'parallel group*' OR crossover OR 'cross over' OR 'single blind*' OR 'double blind*' OR assign* OR allocat*):ti,ab,kw)  #5 #1 AND #2 AND #3 AND #4 | #1 1252131  #2 165191  #3 1729628  #4 5841960  #5 849 |
| Web of Science | TS=((telerehabilitation OR telerehab* OR telemedicine OR telemedic* OR telehealth OR telehealth* OR eHealth OR mHealth OR "mobile health" OR "digital health" OR "digital medicine" OR "digital therapeutics" OR "digital intervention" OR "virtual care" OR "virtual rehabilitation" OR "digital rehabilitation" OR "remote rehabilitation" OR "online rehabilitation" OR "internet based rehabilitation" OR "web based rehabilitation" OR "video based rehabilitation" OR "internet delivered" OR "digitally delivered" OR "online physiotherapy" OR "remote physiotherapy" OR "virtual physiotherapy" OR "online physical therapy" OR "remote physical therapy" OR "virtual physical therapy" OR telephysiotherapy OR telecare OR "remote care" OR teleconsult* OR videoconsult* OR videoconferenc* OR smartphone* OR "smart phone" OR "mobile app" OR "mobile application" OR "mobile phone" OR "cell phone" OR "app based" OR "web app" OR "digital platform" OR "online platform" OR "web platform" OR "computer based" OR "video exercise" OR "exercise video" OR exergam* OR "Wii Fit" OR selfBACK OR SupportBack OR "artificial intelligence" OR "machine learning" OR "AI assisted" OR "automated feedback" OR "motion recognition" OR "posture recognition" OR "movement recognition" OR "real time feedback" OR "wearable sensor" OR "wearable device") AND ("low back pain" OR "lower back pain" OR "back pain" OR backache* OR lumbago OR dorsalgia OR "lumbar pain" OR "lumbar spine pain" OR "spinal pain" OR "mechanical low back pain" OR "nonspecific low back pain" OR "non specific low back pain" OR "non-specific low back pain" OR "chronic low back pain" OR "chronic nonspecific low back pain" OR "chronic non specific low back pain" OR "chronic non-specific low back pain" OR "persistent low back pain" OR "recurrent low back pain" OR sciatica OR "radicular pain" OR "lumbar radiculopathy" OR "disc herniation" OR "disk herniation" OR "lumbar disc disease" OR "spinal degeneration") AND (exercise* OR rehabilitat* OR physiotherap* OR "physical therapy" OR "therapeutic exercise" OR "movement therapy" OR "functional rehabilitation" OR "home exercise" OR "home based exercise" OR "supervised exercise" OR "guided exercise" OR "exercise program" OR "exercise training" OR "exercise prescription" OR "motor control exercise" OR "core stability exercise" OR "core stabilization exercise" OR "stabilization exercise" OR "strengthening exercise" OR "stretching exercise" OR "aerobic exercise" OR "resistance training" OR walking OR Pilates OR yoga OR McKenzie) AND (random* OR randomized OR randomised OR randomly OR randomization OR randomisation OR RCT OR trial* OR "controlled trial" OR "clinical trial" OR "pilot trial" OR "feasibility trial" OR "pragmatic trial")) | All 493 |

# Supplementary Table 5 Inclusion and Eligibility criteria.

| Criteria | Inclusion | Exclusion |
| --- | --- | --- |
| Population | - Patients diagnosed with chronic nonspecific low back pain(CNSLBP). | - Patients with specific causes of LBP(e.g.,spinal fracture,malignancy,infection,cauda equina syndrome)or with acute/subacute pain(duration<3 months). |
| Intervention | - Telerehabilitation must be primarily based on synchronous video-conferencing,asynchronous pre-recorded video exercise programs,or AI-driven interactive exercise platforms that guide patients through physical movement and therapeutic exercises. | - Telephone calls or text messages(mainly used for providing consultation or encouragement); - Virtual reality environments not focused on guided exercise; - Online self-management programs without prescribed exercise components(e.g.,pain education-only platforms,cognitive behavioural therapy apps without exercise); - Web-based interviews or consultations that did not include guided exercise sessions; - Wearable sensors or mobile apps used only for activity tracking or monitoring without providing exercise guidance. |
| Comparison | - The in-person rehabilitation control group consisted of structured,face-to-face,therapist-guided exercise therapy sessions. - The usual care group mainly includes providing educational brochures,leaflets,or general exercise plans with written instructions for family exercises. |  |
| Outcomes | - Pain intensity:Visual Analogue Scale(VAS)or the Numerical Rating Scale(NRS). - Disability/Functional Status:Oswestry Disability Index(ODI)and the Roland-Morris Disability Questionnaire(RMDQ). - Kinesiophobia:Tampa Scale for Kinesiophobia(TSK). | - Incomplete data or unable to extract usable data. - Data not in a usable format for meta-analysis. |
| Type of design | - Randomized controlled trials | - Cohort Study,Case-Control Study,Case Report,Single-Arm Trial,Review. |
| Years of publication | - From inception to September 10,2025 |  |
| Publication type | - Published and unpublished trials | - Protocol - Comment - Letters - Report |
| Language | - English |  |
|  |  |  |

# Supplementary Table 6 Specific meaning of certainty in effect estimates.

| certainty in effect estimates | specific meanings |  |
| --- | --- | --- |
| High certainty | We are very confident that the true effect lies close to that of |  |
|  | the estimate of the effect. |  |
| Moderate certainty  Low certainty  Very low certainty | We are moderately confident in the effect estimate;the true effect is likely to be close to the estimate of the effect,but there is a possibility that it is substantially different.  Our confidence in the effect estimate is limited;the true effect may be substantially different from the estimate of the effect.  We have very little confidence in the effect estimate;the true effect is likely to be substantially different from the estimate of effect. |  |
|  |  |  |

# Supplementary Table 7 Study Numbers and Total Sample Size in Each Network Comparison

| **Comparison** | **Outcome** | **No.of studies** | **Total participants** | **Follow-up** |
| --- | --- | --- | --- | --- |
| TLRH vs IPR | Pain | 3 | 145 | 4 weeks |
| TLRH-AI vs TLRH | Pain | 1 | 34 | 4 weeks |
| TLRH vs UC | Pain | 1 | 44 | 4 weeks |
| IPR vs UC | Pain | 1 | 44 | 4 weeks |
| TLRH vs IPR | Pain | 3 | 172 | 8 weeks |
| TLRH vs UC | Pain | 3 | 188 | 8 weeks |
| TLRH-AI vs TLRH | Pain | 1 | 34 | 8 weeks |
| TLRH vs IPR | Pain | 3 | 359 | 12 weeks |
| TLRH vs UC | Pain | 1 | 177 | 12 weeks |
| TLRH vs IPR | ODI | 3 | 145 | 4 weeks |
| TLRH-AI vs TLRH | ODI | 1 | 34 | 4 weeks |
| TLRH vs UC | ODI | 1 | 44 | 4 weeks |
| IPR vs UC | ODI | 1 | 44 | 4 weeks |
| TLRH vs IPR | ODI | 3 | 172 | 8 weeks |
| TLRH vs UC | ODI | 1 | 50 | 8 weeks |
| TLRH vs IPR | ODI | 2 | 275 | 12 weeks |
| TLRH vs UC | ODI | 1 | 177 | 12 weeks |
| TLRH-AI vs TLRH | RMDQ | 2 | 34 |  |
| TLRH vs UC | RMDQ | 2 | 138 |  |
| TLRH vs IPR | RMDQ | 2 | 94 |  |
| TLRH vs IPR | TSK | 2 | 112 |  |
| TLRH vs UC | TSK | 3 | 154 |  |
| IPR vs UC | TSK | 1 | 44 |  |
|  |  |  |  |  |

# Supplementary Table 8

# Assessment of transitivity across intervention nodes

| Intervention node | No. of studies | Mean age, years (range) | Baseline pain/disability | Intervention duration | Technology types |
| --- | --- | --- | --- | --- | --- |
| TLRH-AI | 2 | 33.2 (29.3-37.1) | Pain: 1 moderate ODI: 1 mild, 1 moderate RMDQ: 1 mild | 4 weeks | AI-assisted guidance APP |
| TLRH | 19 | 43.0 (40.4–47.3) | Pain: 14 moderate, 2 high ODI: 2 mild, 6 moderate RMDQ: 3 mild, 1moderate | 4-12 weeks | Video platform, mobile APP |
| IPR | 8 | 45.4 (43.6–50.0) | Pain: 6 moderate ODI: 5 moderate RMDQ: 1 moderate | 4-12 weeks | Face-to-face supervised rehab |
| UC | 11 | 46.2 (41.7–60.3) | Pain: 6 moderate, 2 high ODI: 1 mild, 2 moderate RMDQ: 2 mild | 4-12 weeks | Not applicable |

# Supplementary Table 9. 95% prediction intervals for key network estimates

| **Outcome** | **Time point** | **Comparison** | **τ used** | **95% PrI** | **GRADE certainty** |
| --- | --- | --- | --- | --- | --- |
| **Pain intensity** | 4 weeks | IPR vs UC | 0.3542 | 0.65 to 2.31 | Low |
| **Pain intensity** | 8 weeks | TLRH vs UC | 0.2937 | 1.01 to 2.47 | Moderate |
| **Pain intensity** | 12 weeks | TLRH vs UC | 0.2489 | 0.64 to 1.82 | Moderate |
| **Pain intensity** | 12 weeks | IPR vs UC | 0.2561 | 0.37 to 1.81 | Moderate |
| **ODI-based disability** | 4 weeks | IPR vs UC | 3.4988 | 2.29 to 17.09 | Low |
| **ODI-based disability** | 8 weeks | TLRH vs UC | 2.6113 | -1.37 to 9.65 | High |
| **ODI-based disability** | 12 weeks | IPR vs UC | 3.4710 | 0.33 to 16.31 | Low |
| **RMDQ-based disability** | Overall | IPR vs UC | 1.0128 | 0.49 to 5.13 | Low |
| **Kinesiophobia / TSK** | Overall | IPR vs UC | 1.4912 | -0.70 to 5.34 | Low |
| **SF-12 PCS** | Overall | TLRH vs UC | 2.4137 | 0.36 to 11.74 | Moderate |
| **SF-12 MCS** | Overall | IPR vs UC | 3.0129 | -4.57 to 10.15 | Low |
| **Pain intensity** | 4 weeks | TLRH-AI vs UC | 0.3634 | 1.21 to 3.31 | Low |
| **Pain intensity** | 8 weeks | TLRH-AI vs UC | 0.2438 | 1.62 to 3.86 | Low |
| **ODI-based disability** | 4 weeks | TLRH-AI vs UC | 3.6317 | 0.50 to 15.86 | Low |
| **RMDQ-based disability** | Overall | TLRH-AI vs UC | 1.2110 | 0.83 to 5.93 | Low |
| **SF-12 PCS** | Overall | TLRH-AI vs UC | 2.3527 | 0.80 to 16.80 | Very low |
| **SF-12 MCS** | Overall | TLRH-AI vs UC | 2.7665 | -12.60 to 17.66 | Very low |

**Abbreviations:** GRADE, Grading of Recommendations Assessment, Development and Evaluation; IPR, in-person rehabilitation; MCS, mental component summary; ODI, Oswestry Disability Index; PCS, physical component summary; PrI, prediction interval; RMDQ, Roland-Morris Disability Questionnaire; SF-12, 12-item Short Form Health Survey; TLRH, telerehabilitation; TLRH-AI, telerehabilitation combined with artificial intelligence; TSK, Tampa Scale for Kinesiophobia; UC, usual care.

# **Supplementary Figure 1** Risk of Bias Graph.


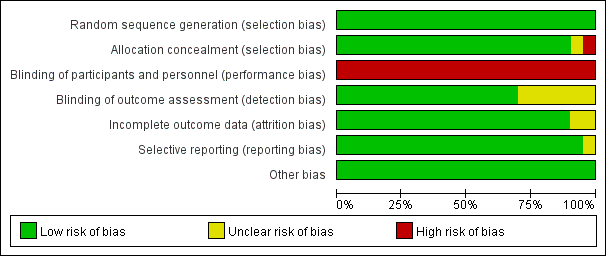


# **Supplementary Figure 2** SUCRA

A

B

C

D

E

F

G

H

I

J

A:Pain intensity in the 4weeks;B:Pain intensity in the 8weeks;C:Pain intensity in the 12weeks;

D:ODI in the 4weeks;E:ODI in the 8weeks;F:ODI in the 12 weeks;G:TSK;H:RMDQ;I:SF-12(PCS);J:SF-12(MCS).

# **Supplementary Figure 3** Funnel Plots for Assessment of Publication Bias.


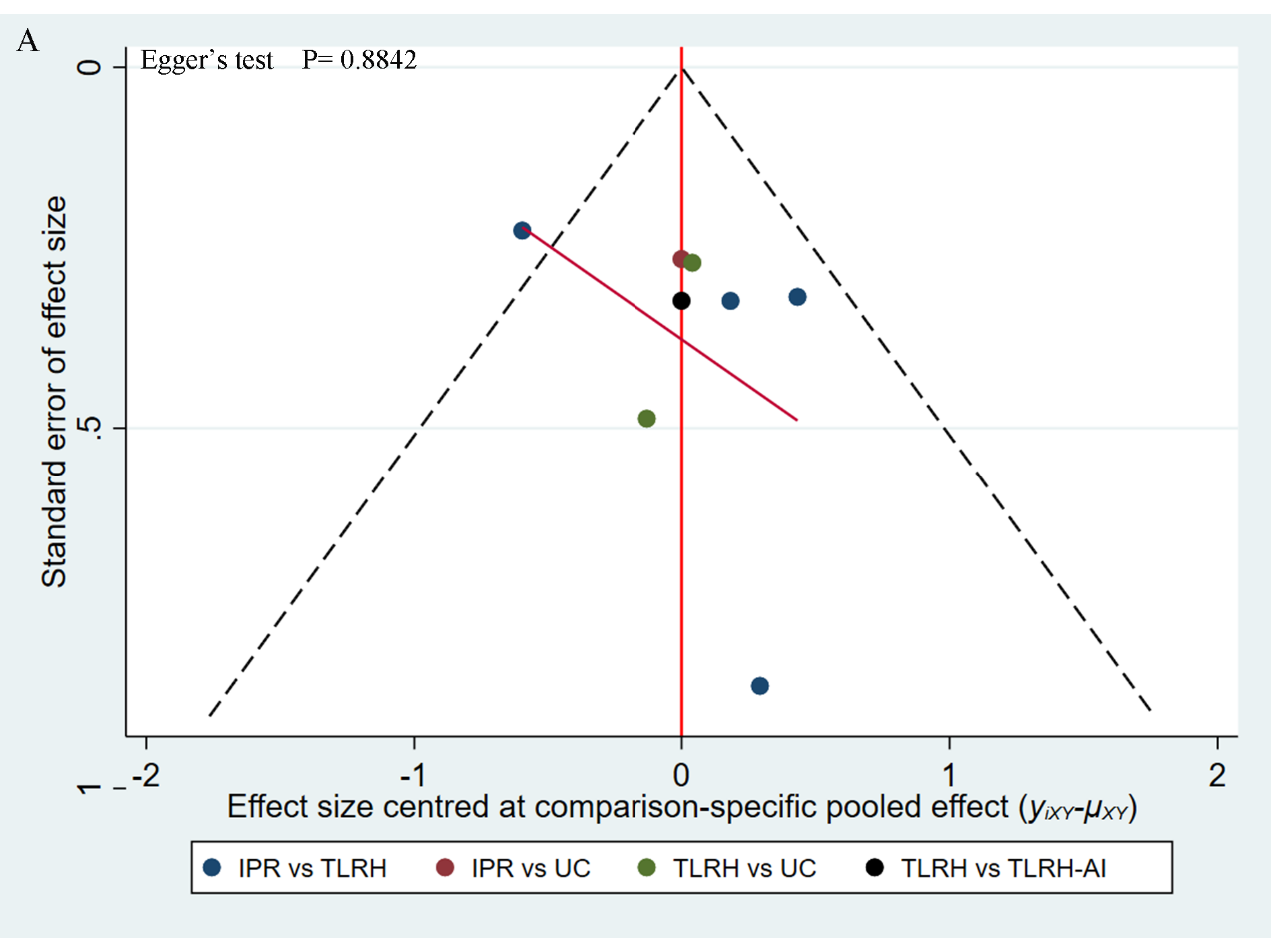


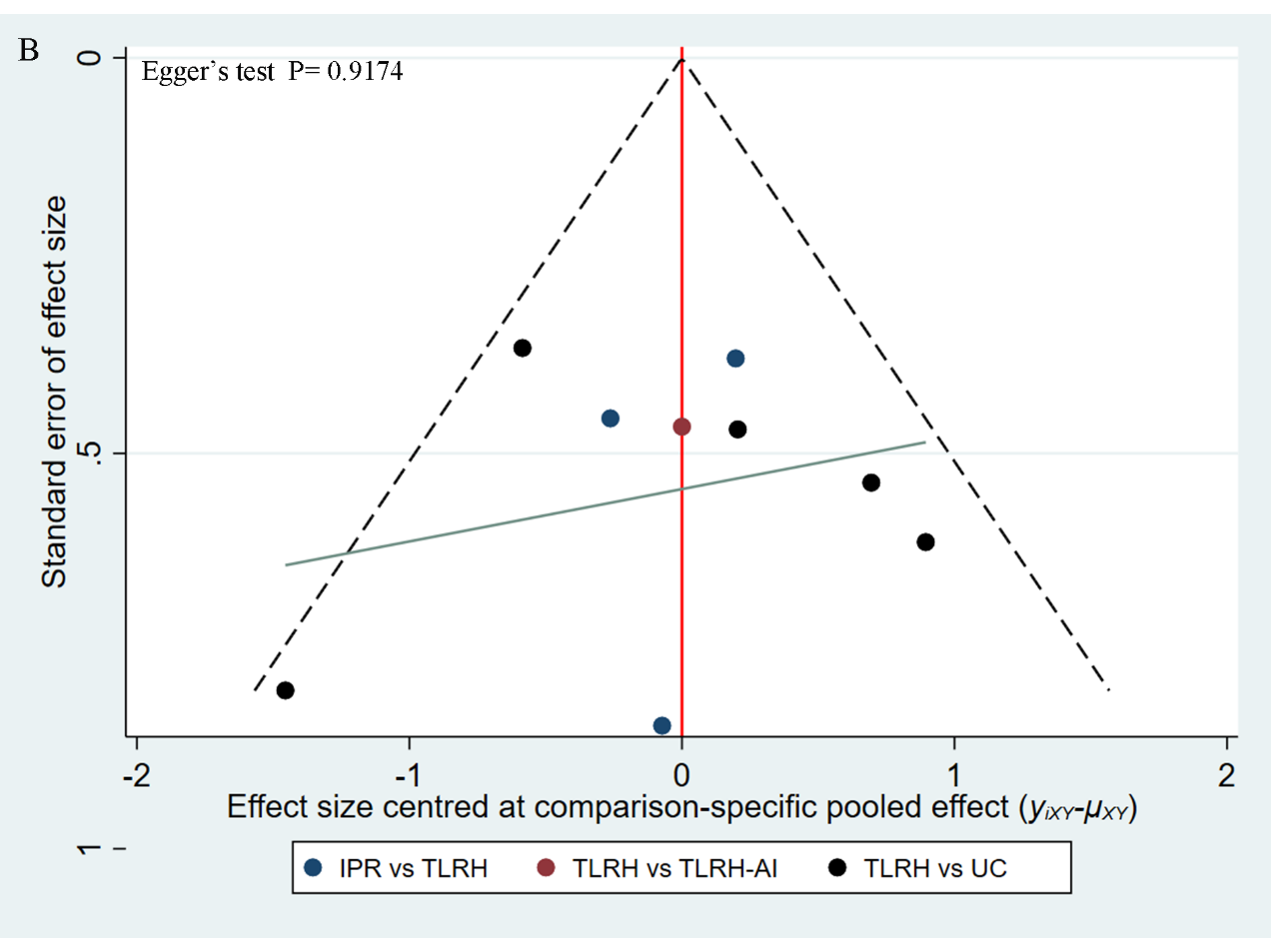


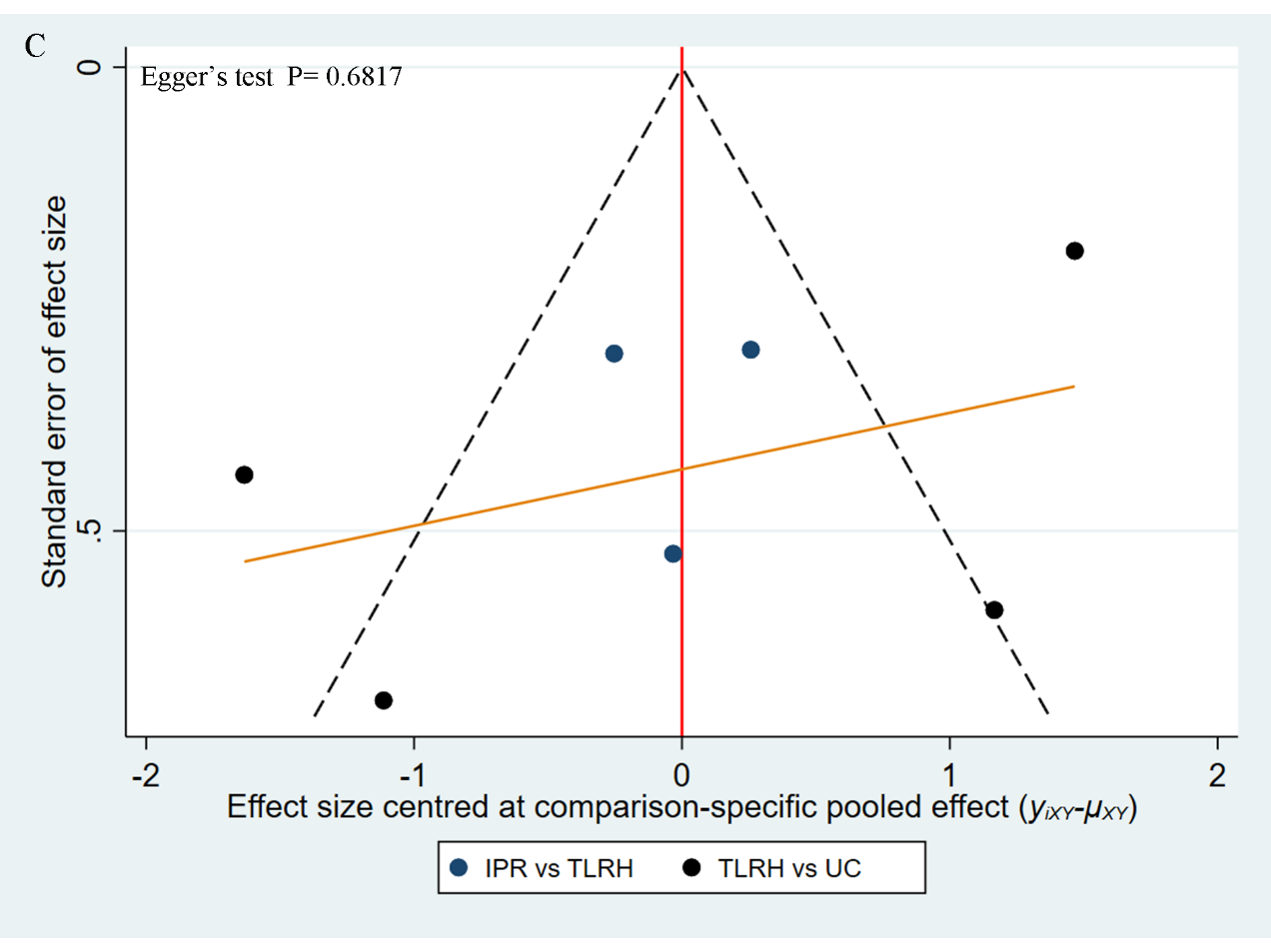


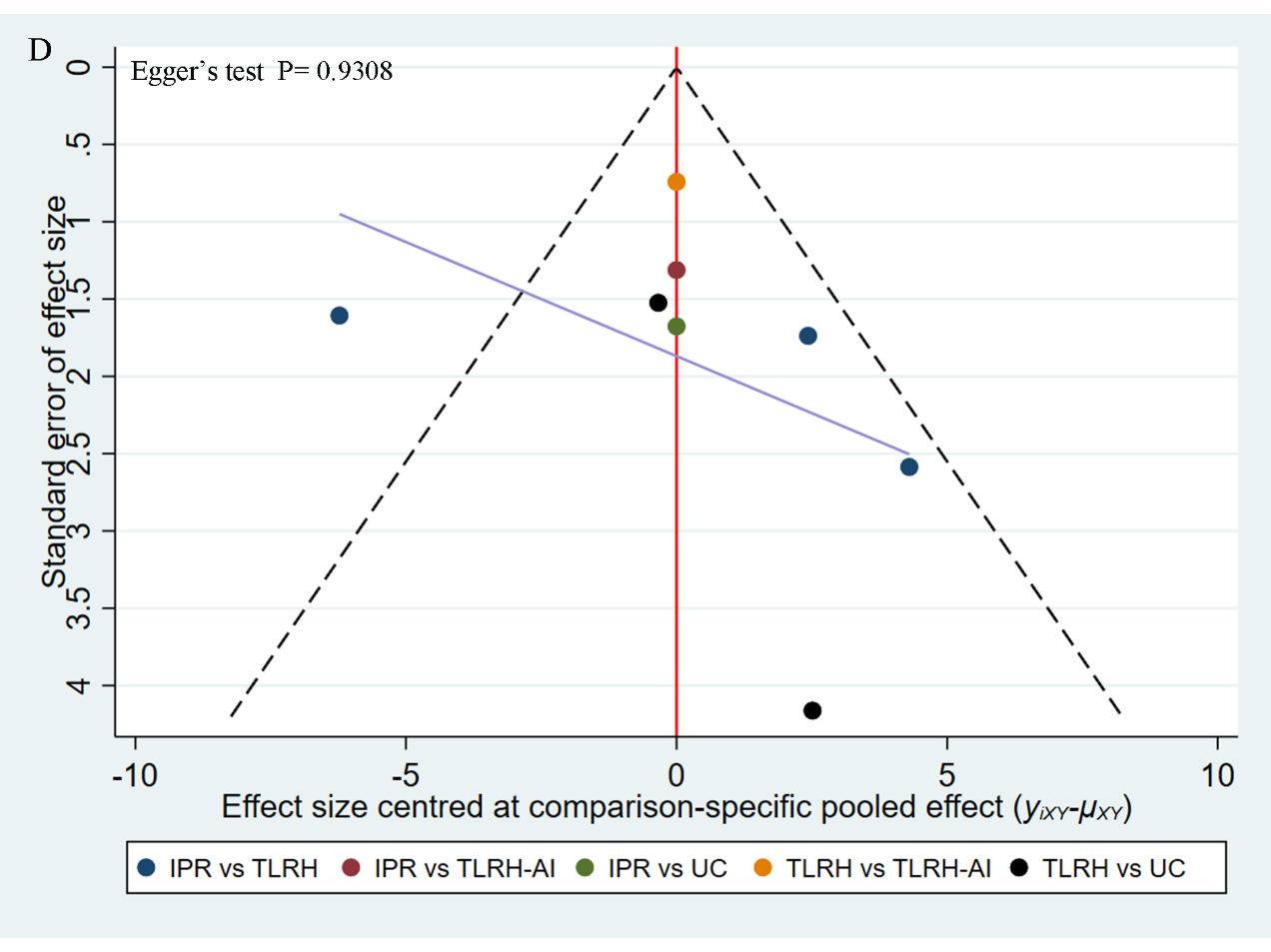


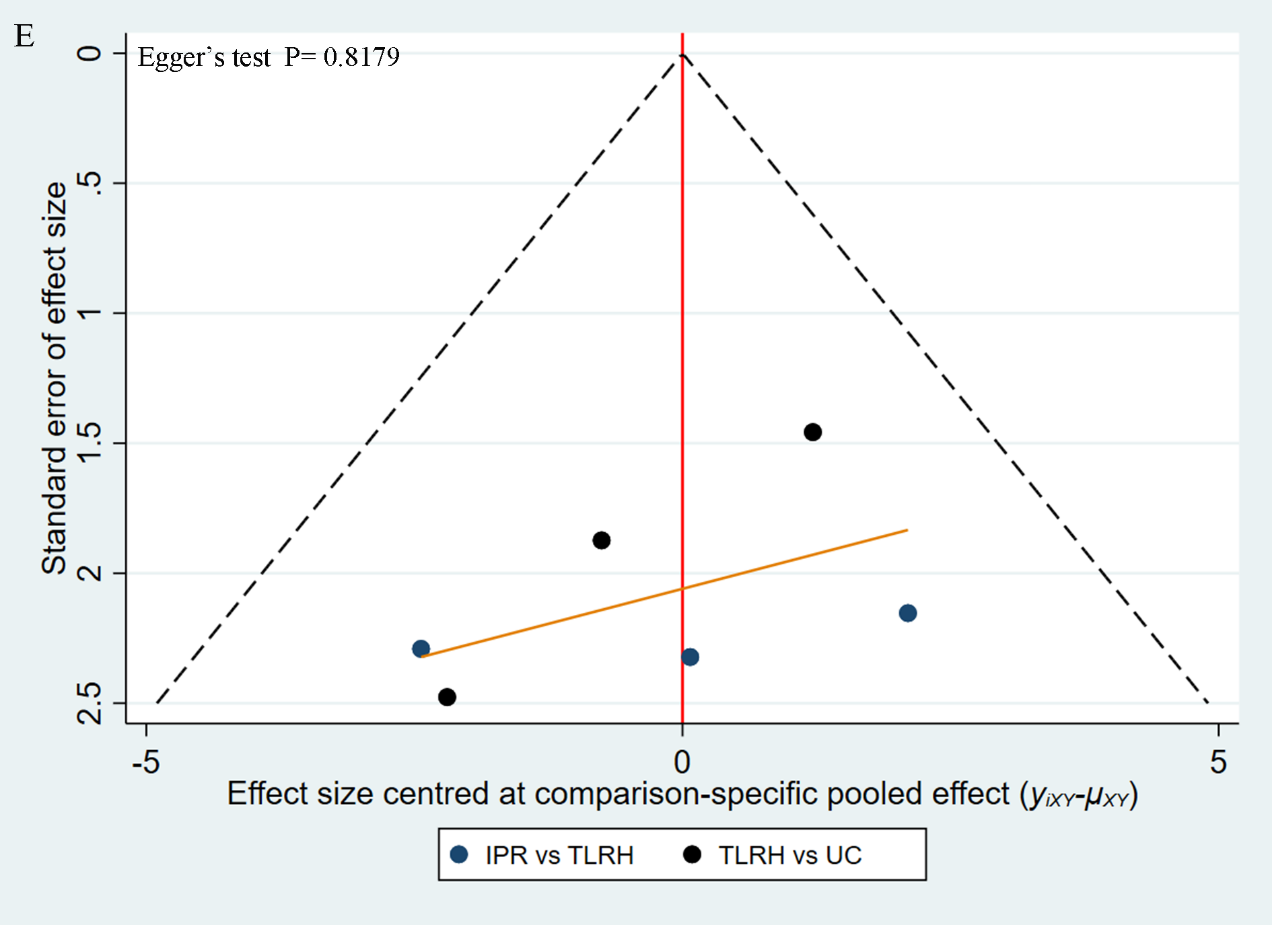


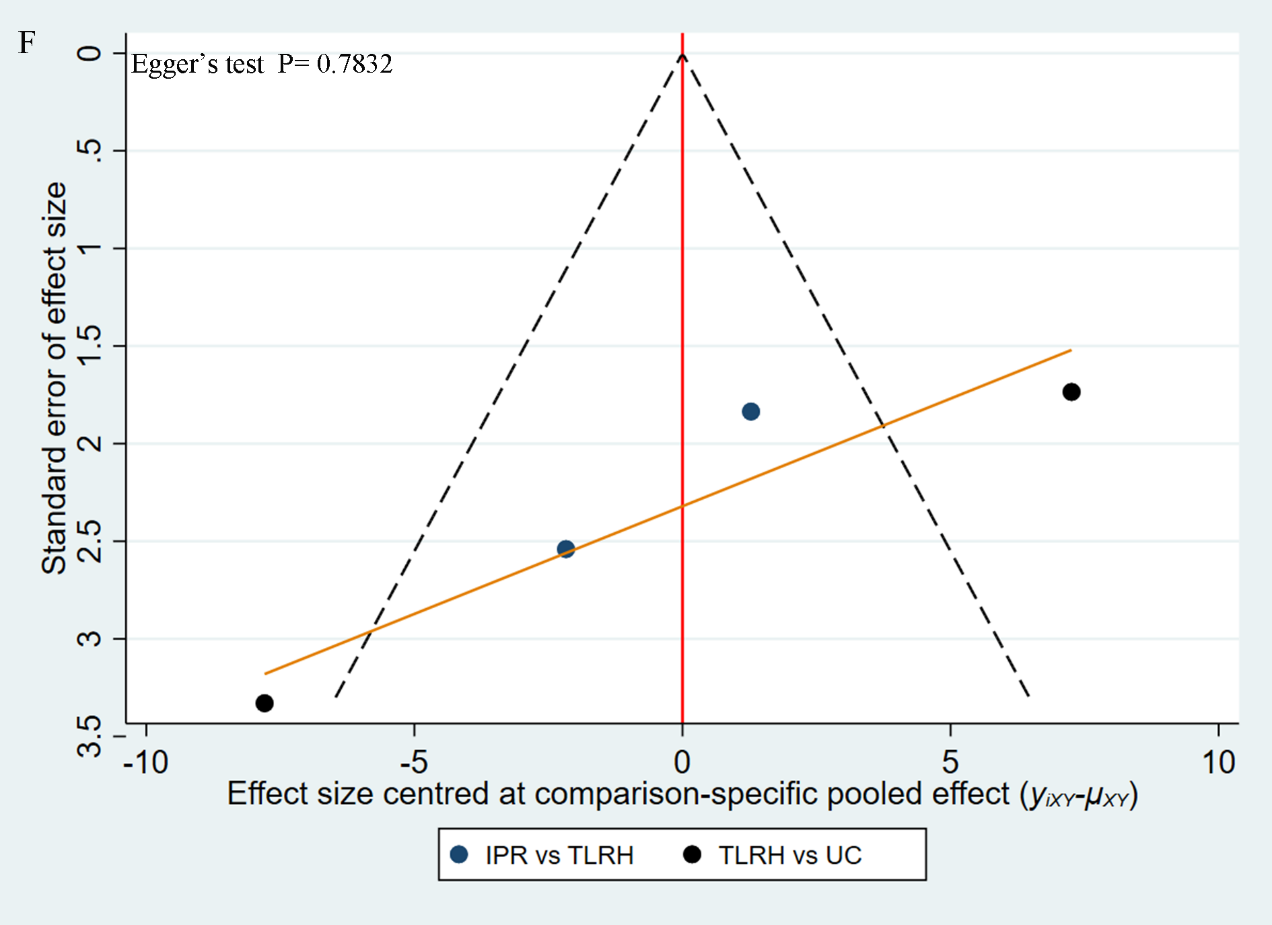


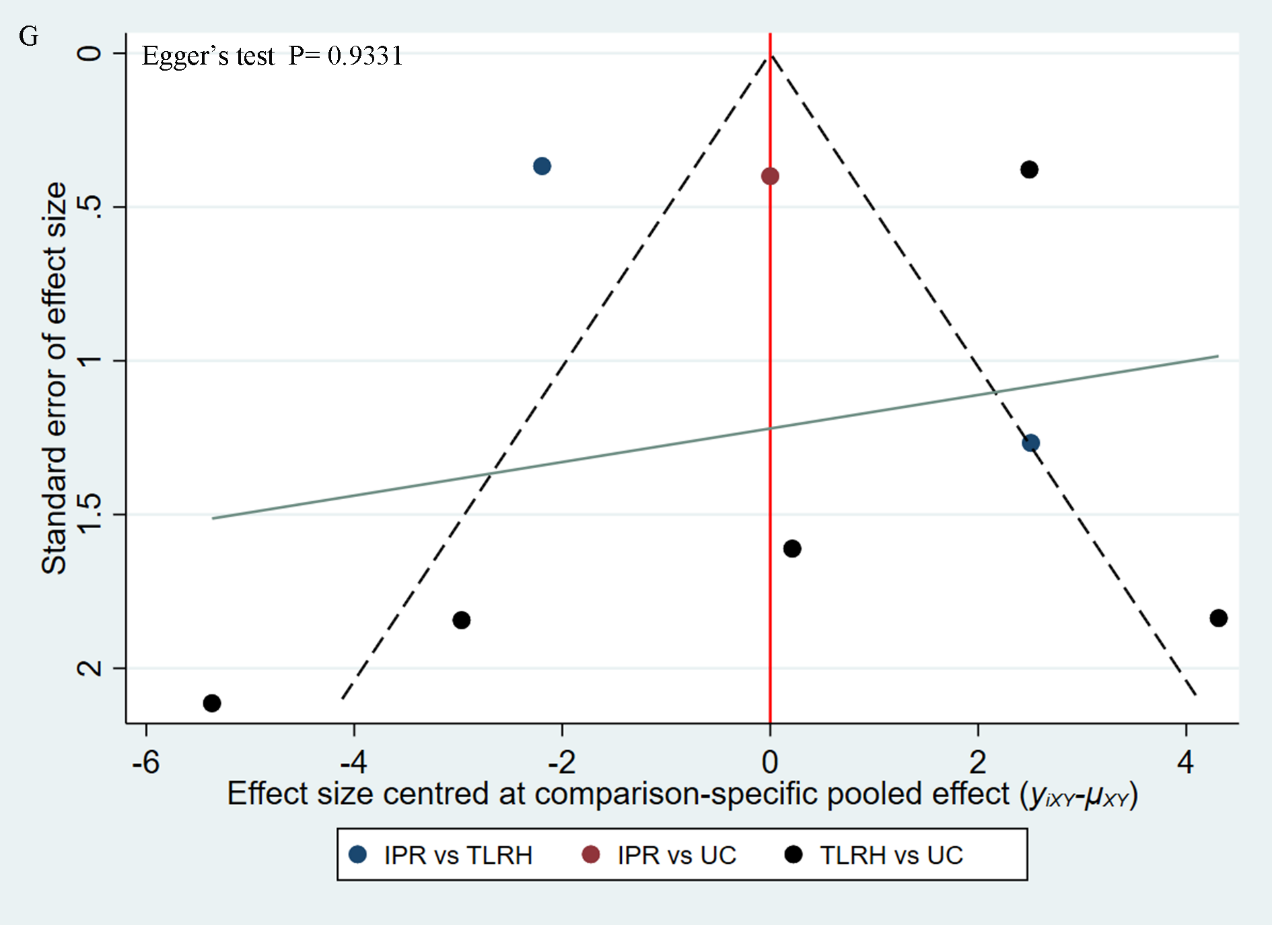


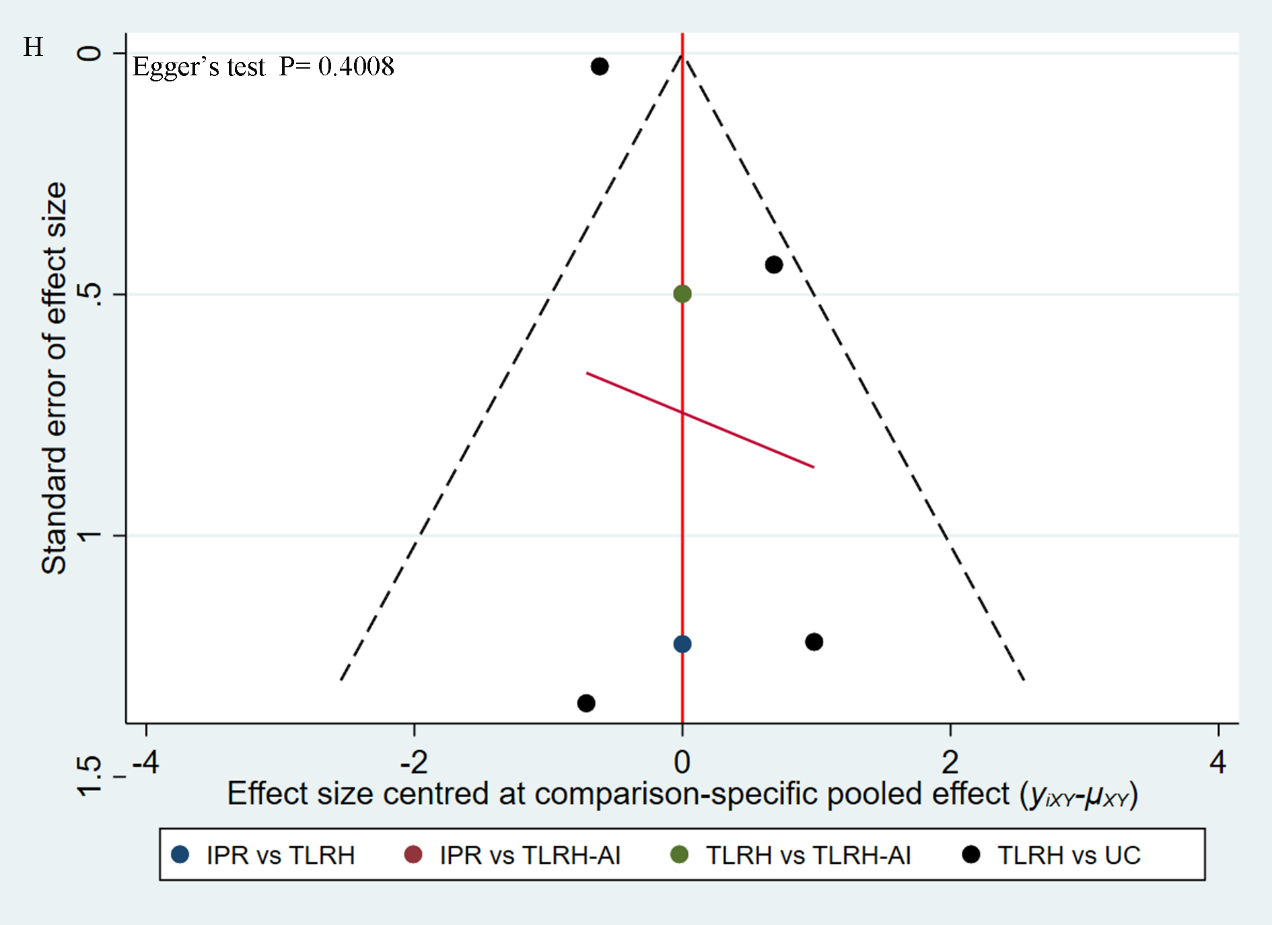


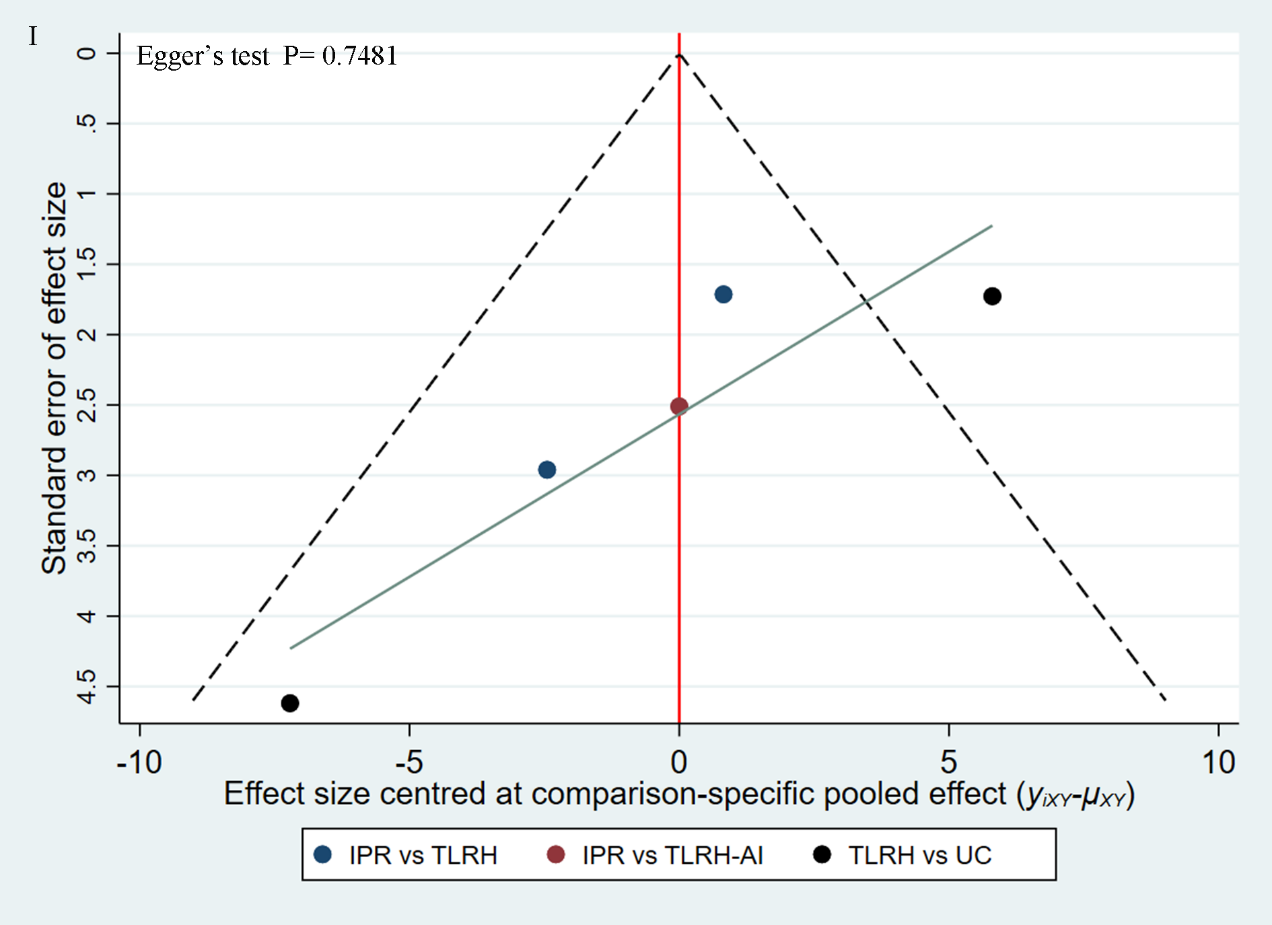


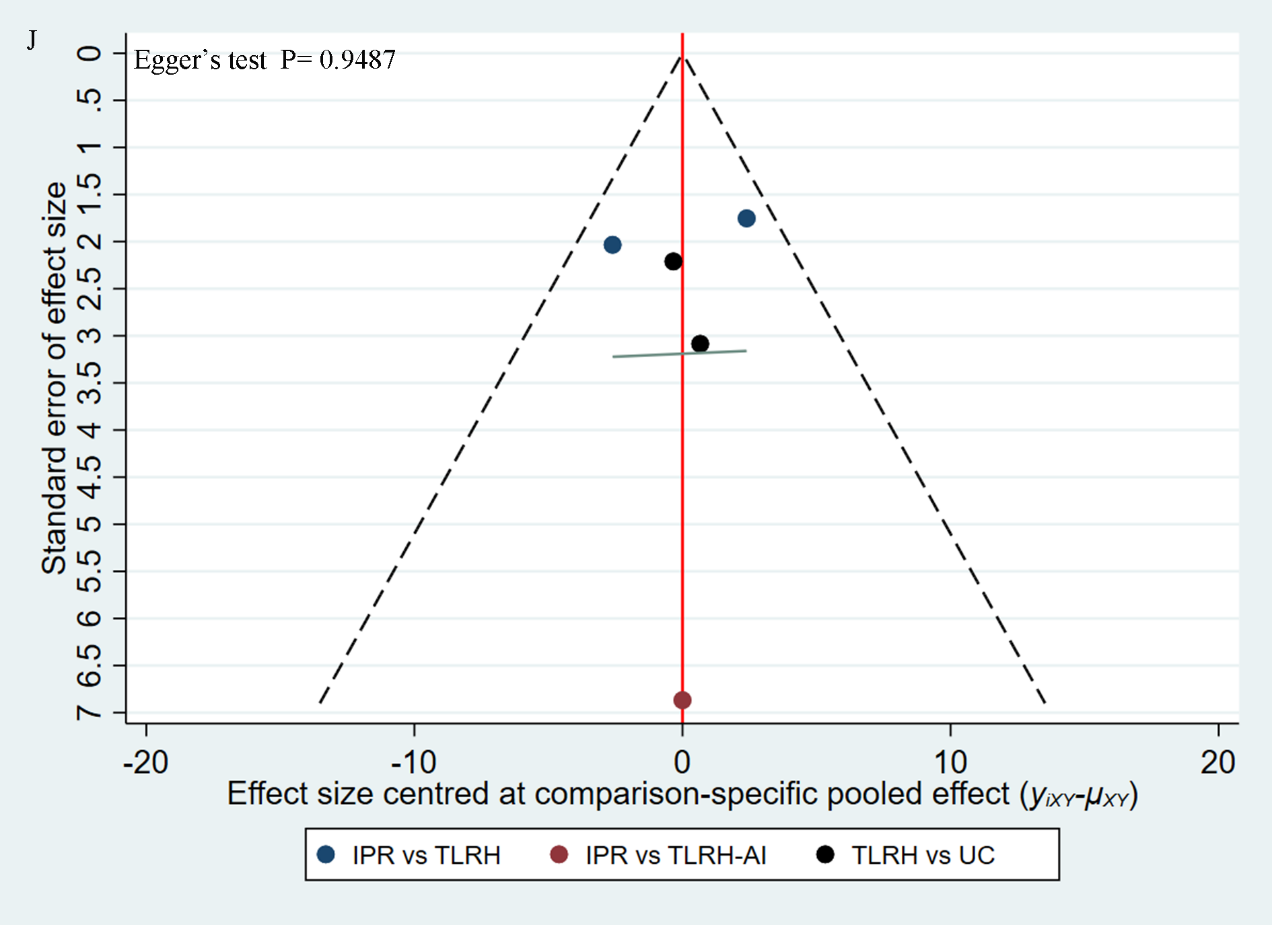


A:Pain intensity in the 4weeks;B:Pain intensity in the 8weeks;C:Pain intensity in the 12weeks;

D:ODI in the 4weeks;E:ODI in the 8weeks;F:ODI in the 12 weeks;G:TSK;H:RMDQ;I:SF-12(PCS);J:SF-12(MCS).

# **Supplementary Figure 4** Test for inconsistency

**A(Pain intensity in the 4weeks)**


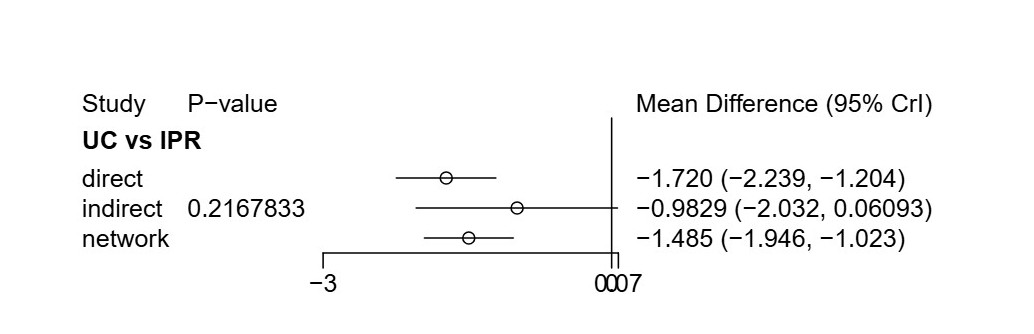


**B(ODI in the 4weeks)**


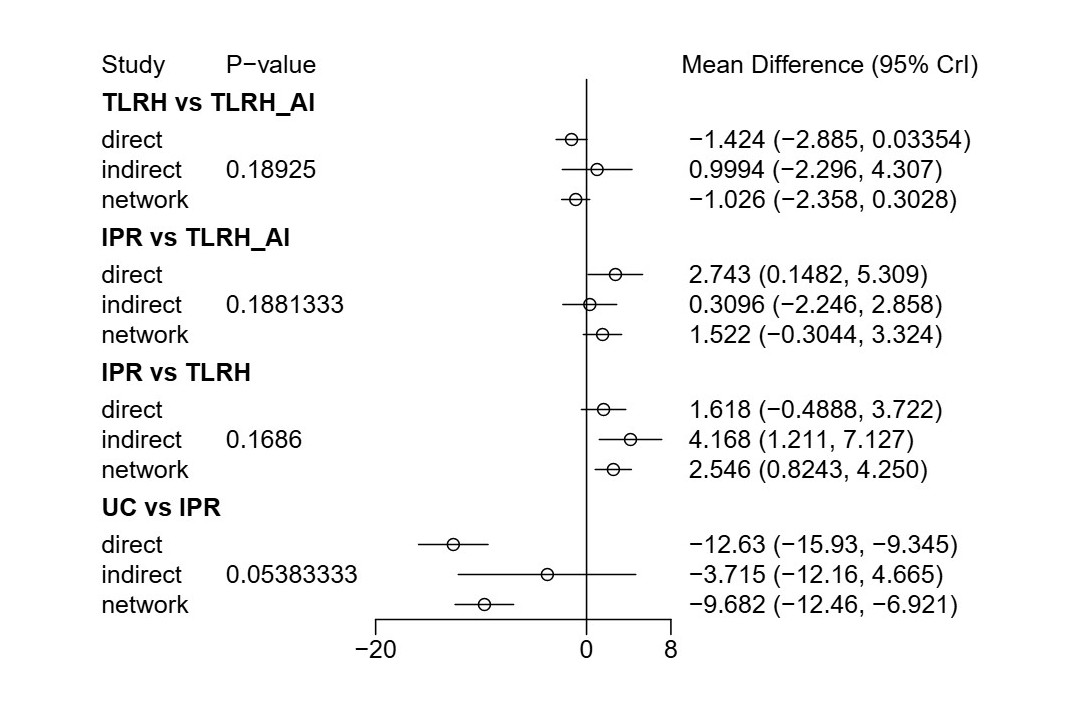


**C(TSK)**


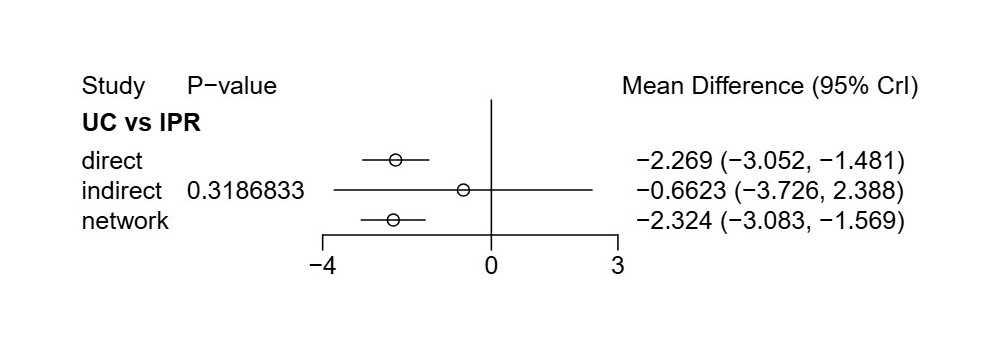


**D(RMDQ)**


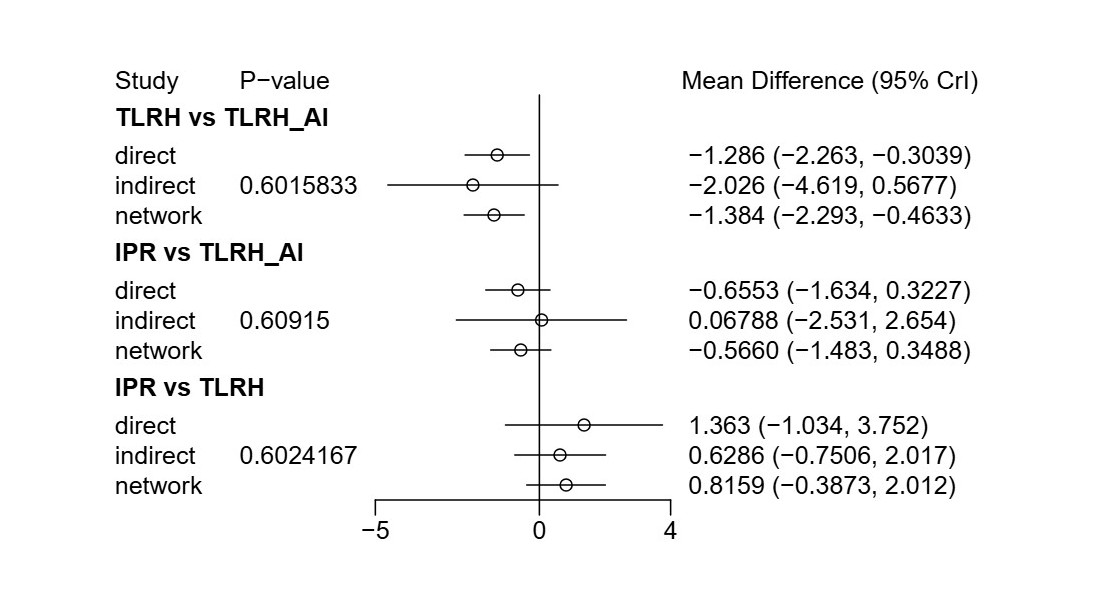

Supplement: Multimedia Appendix 1 [file jmir_v28i1e85410_app1.docx]
